# Edible interfaces based on chitosan, cellulose, and hemicellulose: from food preservation to bioactive delivery and post-consumption relevance

**DOI:** 10.3389/fnut.2026.1877948

**Published:** 2026-08-18

**Authors:** Bing Zhang

**Affiliations:** School of Food and Biological Engineering, Xuzhou University of Technology, Xuzhou, Jiangsu, China

**Keywords:** active packaging, bioactive delivery, cellulose, chitosan, edible films, hemicellulose, intelligent interfaces

## Abstract

The increasing demand for sustainable, functional, and safe food packaging has driven the development of edible polysaccharide-based films and coatings. Cellulose, hemicellulose, and chitosan serve as versatile platforms for constructing multifunctional interfaces that integrate preservation, bioactive delivery, and post-consumption functionality. This review systematically examines strategies to enhance mechanical strength, barrier performance, water resistance, and controlled release through nanoreinforcement, polymer blending, plasticization, crosslinking, and processing optimization. Active preservation functions, including antimicrobial, antifungal, and antioxidant activities, are contextualized with real food applications such as fruits, vegetables, meat, dairy, and lipid-rich products. Special attention is given to bioactive loading and controlled release of phenolics, flavonoids, essential oils, probiotics, and prebiotic xylooligosaccharides, while critically distinguishing formulation-level evidence, such as release in food simulants or simulated gastrointestinal fluids, from unvalidated claims of gut microbiota modulation or nutritional benefit. Food matrix-specific validation illustrates how interface design must match the dominant spoilage pathways of target foods. The post-consumption fate and microbiota-oriented relevance of edible films are discussed as emerging possibilities, with emphasis on the current lack of direct colonic fermentation, animal, or human intervention evidence for many reported systems. Furthermore, advances in intelligent, responsive, and self-healing edible interfaces are explored, along with safety, edibility, sensory acceptability, regulatory considerations, and industrial scalability. Finally, design guidelines for next-generation edible food interfaces are proposed, integrating material selection, polymer engineering, active cargo design, and nutrition-oriented validation. This comprehensive framework provides a roadmap for developing edible interfaces that can improve food quality and sustainability while supporting future investigation of their nutrition-related and post-consumption relevance.

## 1 Introduction

The contemporary food system faces dual challenges: maintaining the quality and safety of perishable foods while reducing reliance on synthetic, petroleum-based packaging materials. Fresh fruits and vegetables are particularly vulnerable after harvest because they remain metabolically active and continue to undergo respiration, transpiration, moisture loss, enzymatic reactions, tissue softening, ripening-related changes, and microbial spoilage during storage (1, 2). These processes are further intensified in fresh-cut or minimally processed products because peeling, cutting, slicing, and bruising disrupt protective tissues, increase exposed surface area, accelerate respiration and ethylene-related responses, promote enzymatic browning and texture deterioration, and provide more favorable conditions for microbial growth (3, 4). Animal-derived products, including meat and fish, are sensitive due to high water activity and nutrient content, which facilitate microbial growth, lipid oxidation, protein degradation, and development of off-flavors (5, 6). Conventional preservation strategies such as refrigeration, controlled or modified atmospheres, chemical treatments, thermal and non-thermal technologies, and biopreservation can extend shelf life. However, their effectiveness depends on the food matrix, processing intensity, cost, and consumer acceptance (7, 8). At the same time, the extensive use of synthetic, non-biodegradable packaging has raised environmental concerns, including plastic accumulation and fossil resource depletion (9, 10). These issues have motivated interest in edible, biodegradable, and bio-based materials capable of maintaining food quality while supporting sustainability-oriented practices (11).

Edible films and coatings have emerged as surface-applied systems that can be consumed along with the food. Applied directly to the food surface, they can regulate moisture migration, gas exchange, oxidative reactions, microbial growth, enzymatic browning, and textural changes (12). Polysaccharide-based coatings are particularly advantageous due to their renewable origin, film-forming ability, and biodegradability (13). Moreover, these coatings enable valorization of agricultural residues and food-processing byproducts as natural polymer sources (14). These edible matrices are not passive barriers. Their properties can be tailored through polymer blending, plasticization, cross-linking, and incorporation of proteins, lipids, or functional bioactive compounds, including essential oils, phenolic extracts, and nanoscale additives (15, 16). Active coatings enriched with plant extracts or essential oils have been shown to limit physiological and microbial spoilage in fresh produce, reducing water loss, ripening, tissue softening, mold growth, yeast contamination, and pathogenic bacteria (17). Similarly, polysaccharide-based edible films incorporating bioactive agents improve physicochemical stability, microbial safety, nutritional content, and sensory properties (18).

Cellulose, hemicellulose, and chitosan provide three complementary polysaccharide platforms for edible food interface design. Cellulose offers a structurally robust backbone suitable for film formation, mechanical reinforcement, and barrier enhancement, especially in the form of derivatives or nanocellulose-based materials such as cellulose nanofibers or cellulose nanocrystals (19, 20). Hemicellulose contributes chemical diversity and fermentability, supporting dietary fiber functionality and prebiotic potential while enabling composite films and coatings (21). Chitosan adds cationic and bioactive functionality through protonated amino groups, potential interactions with negatively charged microbial surfaces, and compatibility with active coatings and controlled-release applications. However, chitosan antimicrobial performance should be considered conditional rather than universally intrinsic. Its activity varies with intrinsic polymer properties such as molecular weight, degree of deacetylation, concentration, and pH-dependent protonation, as well as external conditions such as microbial species and the surrounding medium (22, 23). In edible films and coatings, formulation and food-system variables, including ionic strength, food matrix composition, active additives, and release/contact behavior, further influence whether this antimicrobial potential is expressed in real foods (24, 25). Cellulose, hemicellulose, and chitosan provide complementary but non-interchangeable functions in edible film design. Cellulose derivatives and nanocellulose contribute structural reinforcement and barrier modulation (20, 26), hemicellulose adds flexibility and fermentability-related potential (27, 28), and chitosan provides cationic interactions and antimicrobial potential (23, 25). These complementary roles support rational edible interface design for preservation and bioactive delivery, while post-consumption relevance should be evaluated separately and cannot be inferred solely from material functionality. For post-consumption effects, dietary-polysaccharide literature suggests potential interactions with gut microbiota, but direct validation requires digestion, fermentation, and controlled studies (29, 30).

The incorporation of bioactive compounds further enhances functionality. Essential oils, phenolic extracts, and other antioxidants strengthen antimicrobial and antioxidative performance, aligning with consumer demand for minimally processed foods with fewer synthetic additives (31). Chitosan-based films with natural extracts have demonstrated efficacy across fruits, meats, dairy products, and seafood (32). Lipid-based nanoemulsions or nanoliposomes improve incorporation, stability, and controlled release of sensitive bioactives in meat and fish products, overcoming limitations due to hydrophobicity or volatility (33). Despite these advances, many studies focus primarily on technological endpoints such as film thickness, tensile strength, elongation, water vapor permeability (WVP), oxygen barrier, transparency, antimicrobial inhibition, antioxidant activity, and shelf-life extension (34). While informative, these parameters do not fully capture the nutritional and post-consumption relevance of edible matrices. Coatings can protect bioactive compounds during storage, but they also influence release, stability, and bioaccessibility during oral, gastric, and intestinal phases, particularly for polyphenols, essential oils, probiotics, or prebiotics (25). Post-consumption fate remains underexplored. Dietary polysaccharides can modulate gut microbiota and generate short-chain fatty acids such as acetate, propionate, and butyrate via microbial fermentation (35). Hemicellulose fibers such as arabinoxylans and xylans are particularly relevant due to their structural heterogeneity and fermentability (28). Chitosan and derivatives have also shown potential for gut microbiota modulation, depending on molecular weight, degree of deacetylation, dosage, and formulation (36).

In this review, the term edible interface is used to describe an edible, food-contact material layer positioned at the boundary between the food surface and its surrounding environment and designed to perform functions before, during, and potentially after consumption. This concept includes edible films and coatings but is not intended as a simple replacement term for them. An edible film is generally a preformed, stand-alone sheet that can be applied to or wrapped around food, whereas an edible coating is formed directly on the food surface by dipping, spraying, brushing, or casting. Active packaging mainly refers to systems that release or absorb compounds to extend shelf life, such as antimicrobial or antioxidant films. Intelligent packaging refers to systems that monitor or indicate food quality changes, for example through pH-responsive color changes. By contrast, an edible interface emphasizes the functional boundary itself and evaluates how this boundary simultaneously mediates material structure, food surface interactions, mass transfer, bioactive retention and release, microbial or oxidative deterioration, sensory acceptability, safety, and potential gastrointestinal exposure. The novelty of this framework therefore lies not in re-labeling edible films or coatings, but in reorganizing them around an integrated design and validation logic. Many previous reviews have discussed edible films and coatings as barriers, antimicrobial layers, active packaging systems, intelligent indicators, or delivery matrices. However, these functions are often reviewed as separate technological categories. The edible-interface framework proposed here treats them as interdependent functions of the same food-contact boundary. From this perspective, a successful material is not judged only by improved tensile strength, lower water vapor permeability, antimicrobial inhibition, or release percentage. It must also be evaluated according to how polymer chemistry, interpolymer interactions, surface adhesion, food matrix characteristics, respiration or spoilage pathway, active cargo behavior, release environment, sensory constraints, oral exposure, and regulatory feasibility operate together in a specific food system.

This distinction is scientifically important because the same formulation can have different implications depending on the target food and intended function. For fresh fruits and vegetables, the interface must regulate moisture and gas transfer without inducing anaerobic respiration, off-flavor formation, or abnormal ripening. For meat, seafood, dairy, and lipid-rich foods, antimicrobial and antioxidant functions must be interpreted in relation to microbial ecology, lipid oxidation, water activity, storage temperature, and release kinetics. For bioactive-loaded or post-consumption-oriented systems, release in food simulants or simulated gastric fluid should not be interpreted as evidence of nutritional benefit unless digestion, colonic availability, fermentation, microbiota modulation, dose relevance, and safety are validated. Thus, the edible-interface concept provides a critical framework for distinguishing demonstrated preservation effects from plausible delivery mechanisms and still-unvalidated post-consumption outcomes. Accordingly, this review is organized around cellulose, hemicellulose, and chitosan as complementary edible-interface platforms rather than as isolated film-forming polymers. Cellulose and nanocellulose primarily contribute structural reinforcement, transparency, and barrier modulation; hemicellulose contributes compositional diversity, flexibility, and fermentability-related potential; and chitosan provides cationic interactions, antimicrobial potential, and compatibility with active cargoes. By comparing these platforms across polymer structure, engineering strategy, food matrix-specific performance, controlled release, safety, acceptability, and translational feasibility, this review aims to provide a design-oriented and evidence-aware framework for next-generation edible food interfaces.

## 2 Edible coatings as functional food interface

As defined above, edible interfaces are edible food-contact layers that mediate interactions among the food surface, the surrounding environment, incorporated bioactives, and, after ingestion, the gastrointestinal milieu. This section uses the interface concept as an analytical framework rather than as a new label for conventional edible films and coatings. In conventional descriptions, films and coatings are often discussed according to individual functions such as moisture control, oxygen barrier, antimicrobial activity, antioxidant activity, or active compound delivery. In an interface-based view, these functions are considered together because they occur at the same material-food boundary and influence one another. For example, increasing barrier performance may reduce moisture loss but may also alter gas exchange and respiration in fresh produce; incorporating antimicrobial or antioxidant compounds may improve preservation but can also affect release behavior, sensory quality, migration, and oral exposure; and using prebiotic, probiotic, or colorimetric components may expand functionality but also requires stronger evidence for safety, dose relevance, and real food validation. Therefore, edible-interface design should begin with the target food and its dominant deterioration mechanism rather than with the material alone. The polymer matrix, reinforcement strategy, active cargo, release pathway, and validation method should be selected according to whether the main challenge is moisture loss, gas exchange, microbial growth, lipid oxidation, enzymatic browning, texture loss, bioactive instability, or post-consumption exposure. This design logic allows cellulose, hemicellulose, and chitosan-based systems to be evaluated not only as biodegradable films or coatings, but as integrated food-contact platforms whose performance depends on the coordinated relationship between material structure, food matrix, active function, safety, and intended use. This active role is further enhanced when polysaccharides, proteins, or lipid components are integrated, forming composite structures that exhibit synergistic properties in mechanical reinforcement, barrier performance, and bioactive encapsulation (37). Polysaccharide-based coatings are particularly versatile due to their inherent film-forming capability, biodegradability, and compatibility with food-grade ingredients. Cellulose derivatives, hemicellulose fractions, and chitosan can form matrices with tunable thickness, porosity, and surface charge, which directly influence WVP, oxygen transfer, and microbial resistance (38–40). The formation of hydrogen bonding, electrostatic interactions, and physical entanglement between polymer chains not only strengthens the film but also modulates the release kinetics of incorporated bioactive compounds, including essential oils, polyphenols, and probiotics (41, 42). These interactions are crucial for designing edible interfaces that maintain structural integrity under variable storage and handling conditions while providing controlled functionality.

A critical advancement in edible interface research is the incorporation of bioactive agents within the polymeric matrix. These compounds can range from antioxidants and antimicrobials to vitamins and prebiotic oligosaccharides (43). When encapsulated within a carefully engineered matrix, their stability is enhanced, and their release can be directed towards specific phases of food storage, consumption, or gastrointestinal processing (44). For instance, studies have demonstrated that incorporating polyphenolic extracts into chitosan or cellulose-based coatings significantly improves oxidative stability and inhibits microbial growth on fruit surfaces (45, 46). Prebiotic oligosaccharides can be incorporated into polysaccharide-based films, but current evidence should be interpreted mainly as formulation-level release or carrier feasibility rather than proof of colonic delivery or microbiota modulation. For example, XOS-loaded hemicellulose/chitosan/CNF films have demonstrated rapid XOS release under simulated gastric conditions, whereas colonic availability, fermentability, dose adequacy, and microbiota effects require separate validation (47). The concept of edible food interfaces extends this perspective by framing these materials not merely as coatings but as integral components of food systems with multidimensional roles. An interface is considered functional when it simultaneously preserves food quality, modulates the delivery of bioactive compounds, and influences post-consumption processes, including nutrient availability and microbiota interactions. This framing emphasizes the dual pre- and post-consumption relevance of edible coatings, aligning with current trends in functional and sustainable food systems (48–50).

Recent studies have also explored the architectural and compositional tuning of edible films to achieve tailored performance. Multi-layer or composite films, in which polysaccharides are combined with proteins, lipids, or nanoparticles, can provide selective barrier properties and enhanced mechanical robustness without compromising flexibility or transparency (50–52). Such multilayer systems also enable the spatial localization of bioactive compounds, permitting sequential or controlled release and, in some cases, responsiveness to environmental triggers such as pH, temperature, or enzymatic activity. These innovations underscore the transition from simple coating strategies to engineered edible food interfaces that integrate preservation, active functionality, and nutrition-oriented objectives (53).

**Figure 1:**
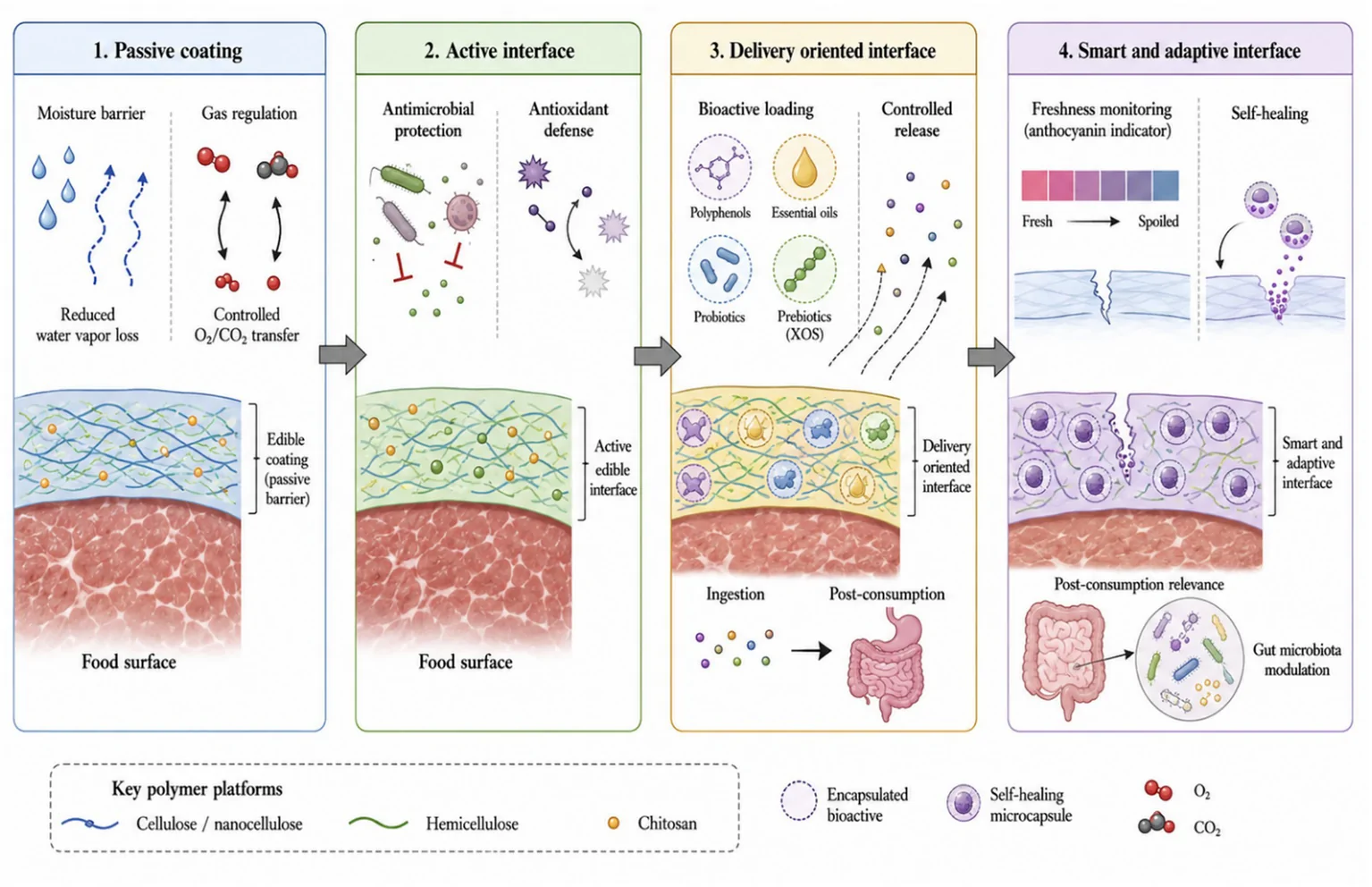
Conceptual evolution from edible coatings to multifunctional food interfaces.

Moreover, understanding the interaction of edible interfaces with the food matrix is fundamental. The interface must accommodate differences in surface energy, roughness, and moisture content while maintaining adherence and integrity over time (54, 55). These material–matrix interactions influence the retention and activity of bioactives and determine the effectiveness of the coating under realistic storage and handling scenarios. In some systems, the incorporation of nanostructured components, such as cellulose nanofibers or chitosan nanoparticles, provides additional reinforcement and can modulate diffusion pathways for water, oxygen, and active compounds (56–59). Overall, this body of research highlights a clear trend: edible films and coatings are increasingly designed as multifunctional food interfaces rather than passive barriers. They integrate structural, barrier, and bioactive delivery functions in a manner that can be tuned according to the specific food matrix, environmental conditions, and intended nutritional outcome. Future edible-interface designs may combine polymer blending, nanocomposites, and controlled-release strategies to improve preservation and to explore post-consumption possibilities. However, microbiota-oriented or health-related functions should remain conditional claims until digestion, colonic fermentation, dose relevance, and biological efficacy are directly validated. This conceptual transition from passive coating layers to multifunctional edible interfaces is illustrated in Figure 1, where preservation, active delivery, environmental responsiveness, and post-consumption relevance are presented as interconnected functions rather than isolated material properties.

## 3 Polymer platforms and structure function design

Polysaccharide-based edible films and coatings rely fundamentally on the structural and functional attributes of their constituent polymers. These biopolymers form matrices that bridge structural integrity with barrier performance and active compound delivery. Edible interfaces must balance mechanical strength with flexibility, moisture and gas barrier properties, and compatibility with bioactive agents to be effective in food applications (60). Polysaccharides also offer inherent biodegradability and non-toxicity, making them attractive alternatives to petroleum-derived packaging materials (61).

Cellulose is the most abundant natural biopolymer on earth, derived from higher plants, algae, bacterial sources, and lignocellulosic residues. Its linear β-1,4-linked glucose structure contributes to high crystallinity and tensile strength (26). Nanocellulose refers to cellulose materials at the nanoscale, including cellulose nanofibrils (CNFs), cellulose nanocrystals (CNCs), and bacterial nanocellulose. These nanoforms exhibit exceptional mechanical performance, increased surface area, and high crystallinity relative to bulk cellulose (62, 63). In edible coatings, nanocellulose functions primarily as a reinforcing filler that enhances mechanical strength and barrier properties. Its high aspect ratio and stiffness enable reduced gas permeability and improved resistance against deformation under stress (64, 65). The addition of CNC or CNF to a polysaccharide film matrix often increases mechanical integrity while maintaining flexibility, with an optimal concentration required to avoid brittleness (66). Cellulose derivatives such as carboxymethyl cellulose (CMC) are widely used in edible film formulations due to their film-forming ability and flexibility. Substituting hydroxyl groups with carboxymethyl groups enhances solubility in aqueous systems and improves compatibility with other polymers like chitosan. Blending cellulose derivatives with additives or other polysaccharides enables modulation of WVP and mechanical resilience, which are critical for edible interfaces applied to high-moisture foods (67–69).

Hemicellulose is a heterogeneous group of branched polysaccharides composed of pentoses and hexoses, often extracted from lignocellulosic biomass. It is less crystalline than cellulose and more easily processed into films due to its amorphous structure (70, 71). Hemicellulose’s diversity in sugar composition allows adjustable moisture affinity and flexibility within edible matrices, making it suitable for composite film systems when blended with other biopolymers (72). However, pure hemicellulose films generally exhibit poor mechanical strength and water resistance on their own. Combining hemicellulose with reinforcing agents, plasticizers, or polymers such as cellulose derivatives or chitosan can improve tensile properties, reduce permeability, and enhance structural coherence (27, 73). The branched structure of xylans and glucomannans contributes to enhanced flexibility and potential fermentability, which are relevant for both film performance and post-consumption interactions in the gut (74).

Chitosan is a cationic polysaccharide obtained by deacetylation of chitin, primarily from crustacean shells. It offers unique functional features including biodegradability, biocompatibility, cationic character, and film-forming capacity. Chitosan is frequently described as an antimicrobial polysaccharide because its protonated amino groups can interact electrostatically with negatively charged microbial cell surfaces, alter membrane permeability, chelate metal ions, and interfere with nutrient transport or cellular integrity (22, 75). However, this activity is conditional rather than universally intrinsic. The antimicrobial effectiveness of chitosan-based films is governed by both polymer-level and formulation-level variables. At the polymer level, molecular weight, degree of deacetylation, concentration, and pH-dependent protonation influence the charge density, solubility, chain mobility, and interaction of chitosan with microbial surfaces (22, 23, 76). At the film and food-interface level, acid type, plasticizer, swelling behavior, release/contact conditions, microbial species, and incorporation of organic acids, essential oils, phenolics, or plant extracts can further modify antimicrobial performance (24, 25, 77). This conditional behavior is especially important in edible food systems because the antimicrobial activity of chitosan is closely linked to pH-dependent protonation of amino groups. Under acidic conditions, protonated amino groups favor electrostatic interactions with negatively charged microbial surfaces, whereas at near-neutral pH, reduced protonation and solubility may weaken these interactions and reduce antimicrobial performance (22, 23). Molecular weight and degree of deacetylation also influence activity by modifying charge density, solubility, chain mobility, film-forming behavior, diffusion, and interaction with microbial surfaces (76, 78).

Food matrix composition further complicates interpretation. For example, proteins in the food matrix can modify the antimicrobial activity of chitosan and chitooligosaccharides through matrix-dependent interactions, while pH, ionic strength, water activity, and film diffusion/contact conditions may further influence antimicrobial performance in real foods (25, 79). Therefore, antimicrobial inhibition observed in agar diffusion, broth culture, or simplified model systems should not be directly generalized to complex foods. In real food matrices, performance must be validated under relevant pH, storage temperature, water activity, surface structure, microbial load, and formulation conditions. Accordingly, chitosan should be described as a conditionally antimicrobial platform whose activity can be enhanced, reduced, or redirected by polymer properties, formulation design, release behavior, and the target food environment. Despite its advantages, pure chitosan films often exhibit limitations in mechanical strength and water vapor barrier performance. Strategies to overcome these drawbacks include blending chitosan with cellulose derivatives, hemicellulose fractions, or incorporating plasticizers and nanomaterials (42, 60). Blends with cellulose, for instance, combine mechanical reinforcement with antimicrobial performance, while lipid or essential oil additions contribute to hydrophobicity and improved barrier behavior. Chitosan composite films also serve as delivery platforms for bioactive compounds. When incorporated with antioxidants or plant extracts, these matrices can release functional agents in a controlled manner, enhancing antimicrobial and antioxidant functionality without compromising structural integrity. This controlled release behavior depends on chitosan’s interaction with the embedded agents and the matrix’s morphology.

To provide a more critical comparative framework, cellulose, hemicellulose, and chitosan should not be treated as interchangeable edible film materials. Each polymer contributes a distinct functionality, but each also introduces limitations that determine its suitability for specific food systems. Cellulose derivatives and nanocellulose are particularly effective when mechanical reinforcement, transparency, dimensional stability, and barrier improvement are required. Their high crystallinity, aspect ratio, and hydrogen-bonding capacity allow them to strengthen polymer networks and create more tortuous pathways for water vapor and oxygen transfer. However, cellulose and nanocellulose mainly contribute structural and barrier functions and should not be assumed to provide the same nutritional or fermentability-related benefits as more readily fermentable hemicellulose fractions. Excessive nanocellulose loading may also increase stiffness, reduce elongation at break, promote aggregation, or alter opacity and surface roughness, which can limit its application on delicate or visually sensitive foods (19, 20, 64). Hemicellulose offers a different functionality profile. Because of its branched and chemically heterogeneous structure, hemicellulose can improve flexibility, support composite film formation, and contribute fermentability-related or prebiotic potential, particularly in xylan- or arabinoxylan-rich systems. Nevertheless, hemicellulose-rich films are often more water-sensitive and mechanically weaker than cellulose-reinforced systems. Their standalone use may therefore be limited in high-moisture foods unless they are blended with cellulose derivatives, chitosan, pectin, proteins, plasticizers, nanofillers, or crosslinkers to improve cohesion, tensile strength, and moisture resistance. Thus, hemicellulose should be viewed as a platform with promising compositional and nutrition-oriented relevance, but not as a universally robust barrier material without additional structural engineering (21, 28). Chitosan provides a third and complementary function because its cationic character enables antimicrobial interactions and compatibility with active cargoes. However, its antimicrobial performance is conditional rather than universal. As discussed above, chitosan activity depends strongly on molecular weight, degree of deacetylation, concentration, pH-dependent protonation and solubility, ionic strength, microbial species, food matrix composition, film swelling, and the presence of organic acids, essential oils, phenolics, or other additives (23, 80, 81). Under near-neutral pH or in protein-, lipid-, or salt-rich matrices, reduced solubility, weaker protonation, and binding to food components may reduce antimicrobial activity compared with simplified acidic *in vitro* assays. Chitosan also has limited water vapor barrier performance when used alone and may raise source-related or sensory concerns depending on the formulation.

Therefore, polymer selection should be guided by the dominant deterioration pathway and intended functionality of the target food. Cellulose and nanocellulose are most useful when structural reinforcement and barrier modulation are priorities; hemicellulose is valuable when flexibility, biomass valorization, and fermentability-related potential are relevant; and chitosan is most appropriate when antimicrobial activity, cationic interactions, and active cargo delivery are required. Composite systems can exploit these complementary functions, but their design should explicitly balance reinforcement, flexibility, water sensitivity, antimicrobial reliability, release behavior, sensory acceptability, and post-consumption relevance. This critical comparison is summarized within the existing comparative framework of Table 1 rather than through an additional table, to avoid overloading the manuscript with redundant tabular material.

**Table 1:** Comparative structure–function framework for cellulose-, hemicellulose-, and chitosan-based edible interfaces.

Polymer platform	Key structural features	Relative advantages	Main limitations	Most suitable food applications	Representative studies
Cellulose derivatives, such as CMC and HPMC	Film-forming, transparent, hydrophilic, chemically modifiable	Good film formation, transparency, compatibility with bioactives, useful carrier matrix	Water sensitivity, limited mechanical strength without reinforcement, moderate moisture barrier performance	Fruit coatings, active films, probiotic or polyphenol delivery systems	Ruan et al. (103); Osorio et al. (109); Ebrahimi et al. (120)
Nanocellulose, including CNC, CNF, MCC, and BCNW	High crystallinity, high aspect ratio, strong hydrogen-bonding capacity, nanoscale reinforcing network	Strong mechanical reinforcement, reduced WVP or reported WVTR, improved oxygen barrier, better thermal stability	Aggregation at high loading, possible opacity, stiffness, or brittleness	Reinforced protein films, transparent fruit packaging, barrier films, mechanically robust coatings	Rojas-Lema et al. (83); Wardak et al. (85); Bilbao-Sáinz et al. (87); Papadaki et al. (89)
Bacterial cellulose	Nanofibrillar network, high purity, high water-holding capacity, strong fibrous scaffold	Excellent structural cohesion, mechanical stability, surface protection, compatibility with CMC, glycerol, yeast, and proteins	Hydrophilicity, processing cost, moisture sensitivity if not blended or modified	Tomato, orange, sausage, high-moisture foods, antimicrobial edible films	Atta et al. (84); Liu et al. (86); Yanti et al. (107)
Hemicellulose	Branched, amorphous, renewable, chemically diverse, fermentability-related potential	Flexible matrices, sustainability, compatibility with chitosan/PVA/CNF, potential prebiotic relevance	Weak mechanical strength alone, water sensitivity, variable composition depending on source and extraction	XOS-containing films, pH-responsive films, active packaging, nutrition-oriented edible interfaces	Xu et al. (47); Wang et al. (108); Wang et al. (132)
Chitosan	Cationic polysaccharide with pH-dependent protonation of amino groups; antimicrobial potential depends on molecular weight, degree of deacetylation, solubility, concentration, and formulation environment.	Conditionally antimicrobial and antifungal platform; electrostatic interaction with microbial surfaces; carrier for organic acids, essential oils, phenolics, nanoparticles, and other active compounds; controlled or pH-responsive release.	Reduced activity under near-neutral pH or high ionic strength; possible binding to proteins, lipids, salts, or polyphenols in complex food matrices; poor water vapor barrier alone; possible sensory effects; crustacean-source and allergen-related concerns; antimicrobial efficacy may not translate from *in vitro* assays to real foods.	Acidic or mildly acidic fruit coatings, antifungal coatings, active films with organic acids, essential oils or phenolics, meat/dairy applications requiring real-food validation, and systems where release behavior and sensory impact are controlled.	Cai et al. (99); Chen et al. (106); Tokatlı and Demirdöven (111); Pavinatto et al. (112)
Protein-polysaccharide blends	Hydrogen bonding, electrostatic interactions, improved cohesion, tunable phase compatibility	Balanced strength, flexibility, barrier performance, and bioactive compatibility	Phase separation, turbidity, or inconsistent properties if polymer ratio is not optimized	Composite films for fruits, meat, cheese, and active packaging	Qi et al. (90); Su et al. (93); Bonilla et al. (135)
Hybrid cellulose/hemicellulose/chitosan systems	Combined reinforcement, flexibility, cationic activity, and release control	Multifunctionality: mechanical support, antimicrobial action, controlled release, and possible post-consumption relevance	More complex formulation, safety and sensory validation required, possible regulatory complexity	Functional edible interfaces requiring preservation plus delivery, such as probiotic/XOS films, polyphenol films, and intelligent systems	Xu et al. (73); Atta et al. (84); Chen et al. (106)

The performance of edible films is governed by intermolecular interactions such as hydrogen bonding, electrostatic interactions, crystallinity, and phase morphology. Hydrogen bonds between hydroxyl, carboxyl, or amino groups increase cohesion within the polymer network, strengthening mechanical properties and reducing free volume for water or gas transfer (60, 82). Electrostatic interactions, especially between oppositely charged polymers like cellulose derivatives and chitosan, can enhance film integrity and reduce defects that compromise barrier properties. These interactions also influence the distribution and release of bioactive compounds within the matrix. Crystallinity and morphology are additional determinants of function. Higher crystallinity generally contributes to improved mechanical strength and reduced permeability, while amorphous domains within the polymer contribute flexibility and sites for bioactive absorption or release. Nanofillers such as CNCs or CNFs can increase crystallinity and improve mechanical performance at low loading levels, enabling the design of multifunctional interfaces with tailored properties.

## 4 Engineering strategies for high performance interfaces

Polysaccharide-based edible films often require engineering strategies to optimize mechanical strength, barrier performance, and water resistance. Pure polysaccharide films, such as cellulose, hemicellulose, or chitosan, are prone to brittleness, limited tensile strength, and high water permeability. To address these limitations, composite strategies incorporating nanofillers, proteins, starches, gums, plasticizers, and crosslinking agents have been extensively explored. These modifications allow for tailored properties in accordance with the intended food application and storage conditions (83–85).

Although this section summarizes reported improvements in tensile strength, water vapor transport, oxygen barrier performance, antimicrobial activity, and release behavior, these values should be interpreted cautiously. Direct numerical comparison across studies is not always appropriate because edible film performance is strongly affected by formulation variables, including polymer source, molecular weight, degree of substitution or deacetylation, plasticizer type and concentration, filler loading, crosslinking density, film thickness, drying conditions, conditioning humidity, and testing protocol. In addition, studies differ in whether they report WVP, WVTR, water resistance, water solubility, or food-level moisture loss, and antimicrobial performance may be assessed using inhibition zones, viable cell counts, food storage trials, or different target microorganisms. Therefore, the numerical values cited in this review are used to illustrate formulation-dependent trends within each study rather than to rank materials across unrelated studies. A balanced interpretation requires considering not only the magnitude of improvement, but also the testing method, film thickness, food matrix, sensory acceptability, safety, scalability, and reproducibility of the formulation.

### 4.1 Nanocellulose reinforcement

Nanocellulose reinforcement provides a high aspect ratio, crystallinity, and stiffness, which significantly enhances mechanical and barrier properties. Faba bean protein films reinforced with 1–7 wt% CNCs demonstrated a progressive increase in tensile strength from 4.6 to 9.8 MPa and Young’s modulus, with optimal dispersion observed via FESEM and FTIR analyses. The presence of CNCs reduced the reported WVTR by ~30%, which was attributed to hydrogen bonding and polymer-nanocrystal interactions that reduced free volume for moisture diffusion (83). Bacterial cellulose (BC) is another potent reinforcing agent. Atta et al. (84) incorporated BC into CMC/yeast/glycerol films for oranges and tomatoes, improving mechanical strength and reducing water loss while maintaining sensory quality. The fibrous BC network interpenetrates the polymer matrix, enhancing tensile strength and elongation at break, and improving resistance to deformation under stress. Similarly, Konjac glucomannan films reinforced with BC nanofibers exhibited increased crystallinity, thermal stability, and tensile strength, while improving oxygen and water vapor barrier properties (86). Microcrystalline cellulose (MCC) has also been used to reinforce hydroxypropyl methylcellulose (HPMC) films. Bilbao-Sáinz et al. (87) demonstrated that 5–10% MCC incorporation increased tensile strength by 48–53% and reduced WVP by up to 50%. Lipid-coated MCC further enhanced moisture barrier performance, emphasizing the role of surface modification in optimizing functional performance.

### 4.2 Polymer blending and network formation

Blending polysaccharides with other biopolymers enhances compatibility, film flexibility, and barrier performance. Faba bean protein/CNC composites exemplify how protein matrices can synergize with nanocellulose to improve mechanical integrity (83). Agar/gelatin films modified with CNCs demonstrated higher tensile strength (>60 MPa) and elongation at break (>21%), with improved water resistance for strawberry preservation (88). Chitosan-based films blended with gelatin, whey protein, or soy protein isolate exhibit improved flexibility, hydrophobicity, and bioactive incorporation capacity. Whey protein/BC nanowhisker films increased tensile strength and Young’s modulus by 32 and 80%, respectively, while reducing WVP by ~34% (89). Soy protein isolate addition to chitosan/CNF films enhanced hydrogen bonding interactions, increased tensile strength by ~18%, reduced oxygen permeability, and improved DPPH and ABTS+ scavenging activity (90). Blending starch with CMC or CNFs is also effective. Thermoformed potato starch films reinforced with activated CNCs showed reduced solubility and WVP while increasing tensile strength (91). Citric acid-crosslinked starch/CNF composites maintained film integrity and reduced moisture transfer in tomato coatings (85). These examples illustrate that biopolymer blending is a versatile tool to simultaneously improve mechanical and barrier performance while facilitating the incorporation of bioactives, enabling the design of tailored edible interfaces.

### 4.3 Plasticization, crosslinking, and processing control

Plasticizers such as glycerol, sorbitol, and xylitol are commonly used to enhance flexibility, elongation at break, and film-forming ability. In hemicellulose/chitosan/CNF films, glycerol concentrations of 20–30% resulted in optimal tensile strength (31–38 MPa) and elongation at break (10–16%), while maintaining acceptable WVP for food packaging applications. Sorbitol and xylitol were effective in increasing tensile strength, though they slightly increased WVP compared to glycerol-plasticized films (73). Chemical crosslinking further enhances water resistance and mechanical performance. Citric acid crosslinking in CMC films improved tensile strength, WVTR, and color retention in coated bananas, effectively delaying ripening and reducing weight loss (92). Maillard reactions between CMC and soy protein isolate enhanced tensile strength and reduced water sensitivity in edible films, providing thermally stable and mechanically robust coatings (93). Dialdehyde CMC crosslinked gelatin films improved tensile strength, thermal stability, UV barrier performance, and swelling resistance, demonstrating the potential of chemical crosslinkers to reinforce polysaccharide matrices (94). Processing conditions also influence film properties. Drying temperature affects thickness, tensile strength, elongation, and water solubility. Lower temperatures generally enhance water retention and flexibility, whereas higher temperatures increase tensile strength but reduce elongation (95). Ultrasonication can improve the dispersion of nanofillers, as demonstrated in PVA-hemicellulose/tea polyphenol films, resulting in enhanced tensile strength (25.61 MPa, +54%) and reduced WVP (96).

### 4.4 Managing performance trade-offs

Although nanoreinforcement, polymer blending, plasticization, crosslinking, and active compound incorporation can improve the performance of edible interfaces, these strategies rarely enhance all properties simultaneously. In many formulations, improvement in one function is accompanied by a reduction in another. Therefore, high-performance edible films should not be evaluated only by maximum tensile strength, lowest WVP, or strongest antimicrobial activity. Instead, they should be optimized according to the required balance among mechanical integrity, flexibility, barrier performance, sensory quality, biological safety, and food matrix compatibility.

A major trade-off is observed between strength and flexibility. Nanocellulose, bacterial cellulose, and cellulose nanocrystals generally increase tensile strength, stiffness, crystallinity, and barrier resistance by forming dense hydrogen-bonded networks within the polymer matrix. However, excessive filler loading may reduce elongation at break, increase brittleness, or cause particle aggregation, leading to defects in the film structure (73, 83, 86, 97, 98). Similarly, crosslinking strategies such as citric acid or dialdehyde-mediated network formation improve water resistance and dimensional stability, but excessive crosslinking can restrict polymer-chain mobility and reduce flexibility (92, 94). This is particularly important for coatings applied to irregular or delicate food surfaces such as berries, figs, tomatoes, and meat products, where the film must deform without cracking during handling and storage. Plasticizers such as glycerol, sorbitol, or xylitol can restore flexibility, but they may also increase WVP, solubility, or moisture sensitivity (73, 94). Therefore, mechanical optimization requires identifying a formulation window rather than simply maximizing reinforcement or crosslink density.

Another important trade-off concerns hydrophobicity and sensory acceptability. Lipids, waxes, essential oils, and essential oil nanoemulsions can improve water resistance, reduce hydrophilicity, and enhance antimicrobial or antioxidant activity. However, these ingredients may also introduce strong odor, altered taste, opacity, surface greasiness, color changes, or reduced consumer acceptance (99–102). For example, essential oils can be highly effective antimicrobial agents, but their volatility and intense aroma may limit their practical loading level in edible coatings. Similarly, plant extracts and phenolic compounds can improve antioxidant activity and UV protection, but they may darken the film, decrease transparency, and modify the visual appearance of coated foods (101, 103, 104). These effects may be acceptable for meat, cheese, or nuts, but less desirable for fresh fruits where gloss, color, and natural appearance strongly influence purchase decisions. Thus, hydrophobic modification should be balanced with optical clarity, odor neutrality, taste compatibility, and the sensory expectations of the target food. A further design challenge is the balance between antimicrobial activity and cytocompatibility. Chitosan, organic acids, essential oils, plant extracts, potassium sorbate, and bioactive nanoparticles may suppress microbial growth, but stronger antimicrobial activity does not automatically indicate suitability for edible use. Because edible films are intended for oral exposure, active components should be evaluated not only against foodborne microorganisms but also for cytotoxicity, migration behavior, release dose, and compatibility with mammalian cells or gastrointestinal conditions (84, 90, 105). This distinction is especially important for formulations containing high concentrations of essential oils, phenolic extracts, antimicrobial salts, nanofillers, live microorganisms, or self-healing microcapsules. Evidence of non-toxicity, together with antimicrobial performance, provides a stronger basis for translation than inhibition-zone data alone.

Several practical limitations should also be emphasized. First, many polysaccharide-based films remain moisture-sensitive, particularly under high relative humidity or when applied to high-moisture foods, which may reduce barrier performance and accelerate swelling or dissolution. Second, mechanical reinforcement can increase stiffness and tensile strength but may also reduce elongation at break, increase brittleness, or impair film conformability on irregular food surfaces. Third, thermal stability and drying behavior are formulation-dependent; higher drying temperatures may improve strength in some systems but reduce flexibility, bioactive stability, or probiotic viability. Fourth, antimicrobial or antioxidant activity measured *in vitro* does not necessarily translate into effective food preservation because release dose, contact time, microbial load, food composition, and storage conditions differ among applications. Finally, scalability and reproducibility remain major challenges. Many edible films are still prepared by solution casting under laboratory conditions, whereas industrial translation requires control of film thickness, drying time, batch-to-batch variability, raw material heterogeneity, microbial safety, cost, and compatibility with coating, dipping, spraying, extrusion, or continuous film-forming processes. These limitations indicate that edible interface design should move beyond reporting isolated improvements and toward standardized, application-specific validation.

These trade-offs highlight the need for application-specific design. For high-moisture fruits and vegetables, flexibility, adhesion, transparency, moisture regulation, and sensory neutrality may be more important than maximum tensile strength. For meat, seafood, and cheese, oxygen barrier properties, antimicrobial action, and antioxidant release may be prioritized, provided that flavor and safety are not compromised. For nutrition-oriented edible interfaces, release behavior, digestibility, prebiotic or probiotic stability, and cytocompatibility should be considered alongside conventional packaging metrics.

Thus, the studies discussed in this section indicate that engineering strategies for polysaccharide-based edible interfaces should be interpreted through general structure–function principles rather than as isolated formulation examples. Nanocellulose, bacterial cellulose, and other nanoscale reinforcements mainly improve mechanical strength and barrier performance by increasing matrix cohesion, reducing free volume, and creating more tortuous pathways for water vapor and gas diffusion. Polymer blending broadens this functionality by combining complementary properties, such as the mechanical integrity of cellulose derivatives, the flexibility and fermentability-related potential of hemicellulose, and the cationic antimicrobial activity of chitosan. Plasticizers and crosslinkers further regulate the balance between chain mobility and network stability, thereby determining whether a film behaves as a flexible coating, a mechanically robust barrier, or a controlled-release matrix. From a mechanistic perspective, the most effective edible interfaces are therefore not those that maximize a single property, but those that coordinate polymer chemistry, network density, filler dispersion, hydrophilic-hydrophobic balance, and bioactive-polymer interactions according to the dominant deterioration pathway of the target food. For high-moisture fruits and vegetables, moisture regulation, adhesion, and respiration control are central design priorities. For meat, seafood, dairy, and lipid-rich foods, antimicrobial and antioxidant activity must be integrated with oxygen and lipid-oxidation control. For functional or post-consumption-oriented foods, matrix swelling, diffusion behavior, and gastrointestinal release become equally important. This synthesis suggests that future edible interface design should move from empirical additive screening toward mechanism-guided formulation, in which structural reinforcement, barrier regulation, active delivery, sensory acceptability, safety, and scalability are optimized together. Overall, the engineering of edible interfaces should move from single-property optimization toward multi-criteria formulation design, where mechanical, barrier, active, sensory, and biological responses are evaluated together. The main engineering strategies used to improve mechanical integrity, barrier performance, water resistance, and active functionality are organized in Table 2, together with their mechanistic basis and potential formulation trade-offs.

**Table 2:** Engineering strategies for high-performance edible interfaces and their formulation trade-offs.

Strategy	Mechanistic basis	Functional gain	Key formulation trade-off	Design implication	Example from reviewed studies	Ref.
Nanocellulose reinforcement	Hydrogen bonding, increased crystallinity, compact network formation, and tortuous diffusion pathways	Higher tensile strength, reduced WVP and OP, improved thermal stability	Excessive loading may reduce flexibility, increase stiffness or brittleness, decrease transparency, or cause aggregation	Use an optimum filler concentration rather than maximum loading; balance reinforcement with elongation and optical clarity	CNC-reinforced faba bean protein films; MCC-reinforced HPMC films; BCNW-reinforced whey protein films	Rojas-Lema et al. (83); Bilbao-Sáinz et al. (87); Papadaki et al. (89)
Bacterial cellulose incorporation	Fibrous network formation, interpenetrating matrix reinforcement, and improved structural cohesion	Improved film cohesion, mechanical stability, moisture barrier performance, food surface protection, and handling resistance	Hydrophilicity may remain high without plasticization, blending, or surface modification; rigid networks may reduce conformability	Combine BC with plasticizers or compatible polymers when coating irregular or soft food surfaces	BC/CMC/glycerol films for sausage; BC/CMC/yeast films for oranges and tomatoes	Atta et al. (84); Yanti et al. (107)
Protein and polysaccharide blending	Hydrogen bonding, electrostatic attraction, phase compatibility, and interpolymer network formation	Better cohesion, improved flexibility, enhanced moisture barrier performance, and improved bioactive compatibility	Phase separation, turbidity, reduced transparency, or inconsistent mechanical behavior may occur if the ratio is not optimized	Optimize polymer ratio and charge compatibility to obtain a continuous and homogeneous matrix	CMC/SPI films; CS/CNF/SPI films; gelatin/chitosan films	Qi et al. (90); Su et al. (93); Wang et al. (132)
Crosslinking	Esterification, Maillard reaction, aldehyde-mediated bonding, and reduced polymer chain mobility	Lower solubility, improved water resistance, reduced WVP, better thermal stability, and reduced swelling	Excessive stiffness, lower elongation, reduced digestibility or release rate, and possible residual crosslinker concerns	Select food-compatible crosslinkers and control crosslinking density according to the target food and release requirement	Citric acid-crosslinked CMC films; Maillard-crosslinked CMC/SPI films; DCMC-crosslinked gelatin films	Nongnual et al. (92); Su et al. (93); Mu et al. (94)
Plasticization	Increased polymer chain mobility, reduced intermolecular rigidity, and reduced brittleness	Higher elongation, flexibility, coating continuity, and better handling properties	Excess plasticizer may increase WVP, solubility, stickiness, and moisture sensitivity while reducing strength	Use plasticizer type and concentration to tune flexibility without compromising WVP or moisture barrier performance	Glycerol-, sorbitol-, and xylitol-plasticized hemicellulose/chitosan films	Xu et al. (73)
Essential oil nanoemulsion loading	Improved dispersion of hydrophobic active phase, reduced droplet size, and controlled release of volatile compounds	Antimicrobial activity, improved hydrophobicity, antioxidant protection, enhanced moisture barrier performance, and controlled release	Strong odor, flavor transfer, opacity, volatility, and possible sensory rejection at high loading	Use nanoemulsification and low effective doses to balance antimicrobial function, moisture barrier performance, flavor, and appearance.	HPMC films with *Thymus daenensis* EO; GG/CS films with orange EO nanoemulsion	Cai et al. (99); Moghimi et al. (102)
Polyphenol loading	Hydrogen bonding, radical scavenging, UV absorption, and interaction with polymer chains	Antioxidant activity, lipid oxidation control, UV protection, color protection, active release, and possible improvement in WVP depending on matrix compatibility	Film darkening, lower transparency, possible taste effects, reduced thermal stability at high loading, or aggregation	Match polyphenol type and dose to the food matrix; consider color compatibility and release environment	EGCG in SA/CMC films; apple polyphenols in cassava starch/CMC films; BA/TP in CS/CMC films	Lin et al. (97); Ruan et al. (103); Chen et al. (106)
Antimicrobial agent enrichment	pH-dependent cationic interactions, membrane disruption, organic acid effects, active compound release, and food-matrix-dependent microbial contact	Microbial inhibition, antifungal activity, and possible shelf-life extension when validated under real food conditions	Strong antimicrobial activity *in vitro* may not translate to real foods; efficacy depends on pH, ionic strength, food composition, swelling, release dose, sensory effects, cytotoxicity, migration, and gut compatibility	Report antimicrobial efficacy together with cytocompatibility or non-toxicity data where possible	Chitosan nanoparticles in tara gum films; yeast-incorporated BC/CMC films; chitosan-based films with plant extracts	Atta et al. (84); Kaya et al. (100); Kumar et al. (101); Antoniou et al. (105)
Probiotic or XOS incorporation	Entrapment, hydration-controlled release, protection of live cells, and prebiotic delivery	Functional delivery, synbiotic potential, and post-consumption relevance	Reduced tensile strength, higher opacity, higher WVP, lower probiotic survival at unsuitable temperatures, and uncertain *in vivo* efficacy	Optimize storage temperature, water activity, film thickness, WVP, and release behavior; avoid overclaiming human nutrition effects	CMC probiotic films; XOS-containing hemicellulose/chitosan/CNF films	Xu et al. (47); Ebrahimi et al. (120)

## 5 Active preservation before consumption

Polysaccharide-based edible interfaces offer active preservation functions that extend beyond passive physical protection. By engineering cellulose, hemicellulose, and chitosan films, it is possible to modulate moisture, gas, UV, and oxygen transfer, as well as microbial proliferation and oxidative degradation, effectively extending the shelf life and quality of diverse food matrices (84, 102, 106). The integration of nanofillers, bioactive compounds, and chemical modifications allows these interfaces to act as functional layers, interacting with the food surface to control the microenvironment in a precise and measurable manner.

### 5.1 Barrier and moisture regulation

For terminology consistency, water vapor permeability (WVP) is used in this review as the main quantitative descriptor of water vapor transport through edible films. Water vapor transmission rate (WVTR) is retained only when it was specifically reported in the original cited study, whereas moisture barrier performance is used as a broader descriptive term referring to the ability of a film or coating to reduce water vapor transfer, moisture loss, or moisture uptake in a food system.

WVP, oxygen permeability (OP), and moisture barrier performance are critical parameters for fruits, vegetables, and lipid-rich foods. Films based on CNCs or BC can reduce water vapor transport by forming dense, hydrogen-bonded networks. In faba bean protein (FBP) films reinforced with 7% CNC, tensile strength increased from 4.6 to 9.8 MPa, while the reported WVTR decreased by 30%, demonstrating a direct relationship between nanoreinforcement and improved moisture barrier performance. The homogeneous dispersion of CNCs within the protein matrix, confirmed via FESEM and FTIR analyses, contributed to compact film structure and decreased free volume for water diffusion (83). Bacterial cellulose-based films also enhance water and oxygen barrier performance. In BC/CMC/glycerol films with 2% yeast incorporation, water solubility reached 42.86%, while maintaining excellent adherence to orange and tomato surfaces, reducing moisture loss and maintaining firmness and color over 2 weeks at temperatures up to 40 °C (84). BC films derived from sago waste with 1% CMC and 1% glycerol exhibited tensile strength of 17.47 MPa and elongation at break of 25.6%, with improved water resistance, demonstrating the effect of matrix stabilization and plasticization on moisture barrier performance (107).

Hemicellulose-based films have been reinforced with CNFs to optimize barrier performance. Citric acid-crosslinked starch films incorporating 8% CNFs provided effective water vapor regulation for tomatoes, reducing weight loss while preserving color and firmness (85). Similarly, hemicellulose/PVA films with blueberry anthocyanin exhibited WVP reduction from 15.43 × 10–13 to 7.27 × 10–13 g·cm/(cm^2^·s·Pa) and oxygen permeability from 13.22 × 10–14 to 3.24 × 10–14 cm^3^·μm/(m^2^·day·Pa), demonstrating that structural crosslinking and functional additives synergistically improve barrier properties while offering pH-responsive freshness monitoring (108). UV-light barrier and oxygen protection are particularly relevant for antioxidant-sensitive foods such as berries and nuts. HPMC films with loaded polyphenols or anthocyanins reduce UV penetration and slow photodegradation, preserving internal bioactive content and delaying deterioration (109). Controlled moisture and gas regulation can reduce respiration rate, water loss, and oxidative stress in perishable foods, but the barrier effect must remain selective rather than excessive. In fresh produce, overly low oxygen permeability or excessive carbon dioxide accumulation may disturb normal metabolism and promote anaerobic respiration, ethanol or acetaldehyde accumulation, off-flavor development, tissue injury, or altered ripening (1, 3, 110). These barrier effects, combined with appropriate plasticization and reinforcement, form the foundation for active edible coatings tailored to specific food matrices.

### 5.2 Antimicrobial and antifungal activity

Chitosan-based matrices can show antimicrobial activity, mainly through pH-dependent protonation of amino groups and electrostatic interactions with negatively charged microbial surfaces. However, this activity is conditional and depends on polymer properties, formulation variables, microbial species, and the food matrix environment (22, 23). Edible coatings incorporating chitosan nanoparticles or blended with gums, proteins, and polysaccharides demonstrate enhanced bactericidal and antifungal properties. Tara gum films containing chitosan nanoparticles exhibited increased tensile strength (35.73 MPa) and decreased WVP (22.7%), alongside significant antimicrobial activity against *E. coli* and *S. aureus* (105). Practical applications on fruits confirm these effects. Sweet cherries coated with 1% chitosan maintained microbial counts below detectable levels over 25 days at 4 °C, while uncoated controls exhibited 2.74 log CFU/g (111). Strawberries coated with chitosan/glycerol composites resisted gray mold infection, retaining sensory and textural quality (112).

Incorporation of bioactive plant extracts further amplifies antimicrobial activity. Chitosan films enriched with pomegranate peel extract inhibited microbial proliferation in a concentration-dependent manner while enhancing antioxidant activity (101). Films formulated with Berberis crataegina seed oil and fruit extract improved thermal stability, UV barrier properties, and antimicrobial efficacy simultaneously, demonstrating that natural bioactives can complement the polysaccharide matrix for multifunctional protection (100). For protein-rich and fatty foods, whey protein–BCNW composites maintained low microbial counts and preserved physicochemical quality during refrigerated storage (89). Hemicellulose-based films incorporating carvacrol achieved >4 log reductions in *Listeria*, *E. coli*, and *Salmonella*, demonstrating strong antimicrobial potential under the tested conditions (113). However, such reductions should be interpreted as evidence of antimicrobial inhibition rather than direct proof of shelf-life extension unless they are confirmed in real food matrices under relevant storage conditions.

The antimicrobial role of chitosan-based films should be interpreted in relation to formulation and food-matrix conditions. Chitosan activity is commonly stronger in acidic environments, where amino groups are protonated and electrostatic interaction with microbial surfaces is favored. In contrast, near-neutral foods, high ionic strength, high protein or lipid content, and limited film swelling may reduce chitosan solubility, diffusion, and microbial contact. Therefore, inhibition zones or microbial reductions obtained in simplified *in vitro* assays should be considered preliminary indicators of antimicrobial potential rather than direct evidence of preservation efficacy in real foods. For chitosan-containing edible interfaces, antimicrobial performance should be validated using food-relevant pH, water activity, storage temperature, target microorganism, food matrix, coating contact, and release conditions (23, 25, 79). For fruit coatings specifically, chitosan performance should also be interpreted in relation to postharvest physiology, commodity type, maturity stage, and storage conditions (114, 115).

### 5.3 Antioxidant protection and shelf-life extension

Polysaccharide-based films can also act as active antioxidants, particularly when enriched with polyphenols, anthocyanins, or flavonoids. These bioactive components scavenge free radicals, prevent lipid oxidation, and maintain food color and texture. Chitosan–CMC films loaded with blackberry anthocyanins and tea polyphenols preserved chilled beef, maintaining color, firmness, and oxidative stability during two-week storage at 4 °C (106). CMC-based films with EGCG demonstrated controlled release into fatty food simulants, effectively inhibiting peroxidation (103). Thyme extract incorporated into chitosan-starch films significantly enhanced DPPH scavenging capacity and ferric reducing power, offering active protection against oxidation (104). Pomegranate peel-enriched chitosan films have been reported to improve antioxidant activity and oxygen-barrier-related performance under the tested conditions, suggesting preservation potential that should be interpreted according to the specific food matrix and storage system used in the original study (101).

Shelf-life extension should be distinguished from antimicrobial inhibition measured in simplified assays. Inhibition zones, minimum inhibitory concentrations, or *in vitro* viable-count reductions indicate antimicrobial potential, but real-food shelf-life extension requires preservation of sensory, physicochemical, and microbiological quality under realistic storage conditions. Food composition, pH, water activity, fat content, surface roughness, initial microbial load, storage temperature, coating adhesion, and release kinetics can all modify antimicrobial performance in real foods. Therefore, shelf-life extension should be discussed only when antimicrobial activity is connected to food-level outcomes such as microbial counts, oxidation indices, pH, firmness, color, texture, odor, or sensory acceptability during storage (1, 25, 110, 116). BC/CMC/yeast films maintained firmness and sensory quality in oranges and tomatoes at 6–40 °C (84). Chitosan-CS-CNF films with flavonoids extended chilled beef shelf life, maintaining color, microbial safety, and oxidative stability (117). Guar gum/chitosan films with orange essential oil nanoemulsions preserved cheese quality by controlling moisture, oxidation, and microbial proliferation (99). UV-activated self-healing hemicellulose/chitosan films containing Omega-3 microcapsules extended cashew shelf life, demonstrating the integration of active preservation and functional nutrient delivery (118). Thus, cellulose-, hemicellulose-, and chitosan-based edible films can function as active food interfaces by combining barrier, antimicrobial, and antioxidant mechanisms. However, the contribution of each mechanism to real-food preservation should be verified under food-specific conditions rather than inferred from isolated antimicrobial or antioxidant assays. The combination of polysaccharide matrices with nanofillers, plasticizers, crosslinkers, and natural bioactives can support tailored preservation strategies, provided that safety, sensory quality, release behavior, and storage performance are validated in the intended food system.

Overall, the real-food examples discussed in this section indicate that active preservation should be designed according to the dominant spoilage pathway of each food matrix rather than by simply adding antimicrobial or antioxidant agents to a film. For fresh fruits and vegetables, the main design priorities are moisture regulation, respiration control, firmness retention, color stability, and inhibition of yeasts, molds, and surface pathogens. In meat, seafood, dairy, and lipid-rich foods, the interface must more strongly address microbial proliferation, lipid oxidation, protein degradation, pH change, texture deterioration, and off-flavor formation. Therefore, polymer selection, hydrophilic-hydrophobic balance, oxygen and water vapor transport, and the release profile of active compounds should be matched to the specific deterioration mechanism of the target food. This matrix-specific interpretation shifts edible interface design from empirical preservation trials toward rational formulation based on food composition, spoilage kinetics, and storage conditions.

## 6 Bioactive delivery and release design

Polysaccharide-based edible films have evolved into multifunctional delivery systems, capable of combining mechanical reinforcement, barrier functionality, and controlled release of bioactive compounds. Beyond their traditional role as passive coatings, these films actively mediate preservation while enabling nutraceutical and prebiotic delivery, bridging food storage and post-consumption relevance (47, 84, 106). Engineering such systems requires a careful balance between polymer composition, bioactive interactions, and processing parameters, as these factors collectively determine film integrity, release kinetics, and functional efficacy.

### 6.1 Polyphenol-loaded active matrices

Phenolics and flavonoids are widely incorporated into chitosan-, cellulose-, and hemicellulose-based films to impart antioxidant and antimicrobial properties. For instance, chitosan–CMC films loaded with blackberry anthocyanins (BA) and tea polyphenols (TP) demonstrated enhanced tensile strength, reduced WVP, and improved UV–vis blocking, effectively preserving chilled beef during 14 days of storage at 4 °C (106). FTIR and ^1^H NMR analyses revealed hydrogen bonding and intermolecular interactions between TP, BA, chitosan, and CMC, which stabilized the bioactives and moderated their release. The films inhibited the growth of key pathogens, including *L. monocytogenes*, *S. typhimurium*, *E. coli*, and *S. aureus*, illustrating dual-functionality in preservation and nutraceutical delivery.

Similarly, cassava starch/CMC films incorporating apple polyphenols (AP) improved barrier properties, reducing water vapor transmission and peroxide values, while maintaining slight reductions in tensile strength. Microscopic analysis indicated enhanced crystallinity and compactness, which facilitated both mechanical stability and controlled bioactive release (97). Chitosan films incorporating flavonoids such as catechin, quercetin, or luteolin showed concentration-dependent enhancements in tensile strength (up to 240%) and elongation at break (up to 220%), with optimal bioactivity observed at 5% loading; higher concentrations caused minor decreases in mechanical properties due to aggregation (95). Hemicellulose/pectin/nanocellulose biocomposites containing mango peel polyphenols offered slow and sustained release (~0.021 mg/h) into fatty food simulants while enhancing antioxidant activity (~22.5%) over 48 h (119). These findings highlight the utility of nanocomposite architectures to regulate bioactive release and preserve activity in lipid-rich food systems.

### 6.2 Essential oil nanoemulsion systems

Essential oils (EOs) are highly effective antimicrobial and antioxidant agents, yet their volatility and hydrophobicity pose challenges for uniform incorporation. Nanoemulsion strategies address these limitations, enhancing film homogeneity, stability, and controlled release. HPMC films loaded with Thymus daenensis EO nanoemulsions exhibited significant inhibition against *S. aureus* (22–47 mm zones), maintaining flexibility and optical clarity while delivering antimicrobial activity (102). Guar gum/chitosan films reinforced with orange EO nanoemulsions (OEON) exhibited increased hydrophobicity (contact angle 113.8°), reduced water solubility (~69.8%), and improved elongation at break (~135%), demonstrating superior mechanical and barrier performance (99). Application to cheese showed reduced microbial growth, minimized lipid oxidation, and maintenance of pH, texture, and color. Likewise, partial coatings using citric acid-crosslinked starch and cellulose nanofibers preserved tomato fruits by minimizing weight loss and maintaining firmness and color, while providing a robust platform for EO incorporation (85). Even in yeast-incorporated BC/CMC films, although the primary function was microbial antimicrobial activity, the matrix compatibility suggests that EO integration could further enhance preservative effects in high-moisture foods such as oranges and tomatoes (84).

### 6.3 Probiotic and prebiotic delivery

Edible films can also serve as carriers for live microorganisms and prebiotics, enabling synbiotic functionality. CMC-based films immobilized *Lactobacillus acidophilus*, *L. rhamnosus*, and *Bifidobacterium bifidum*, maintaining viable populations above 10^7^ CFU/g over 42 days at 4 °C (120). Film thickness, polymer concentration, and WVP significantly influenced microbial survival. Hemicellulose/chitosan films reinforced with cellulose nanofibers and containing xylooligosaccharides (XOS) represent a useful example of prebiotic-loaded edible films, but the evidence should be interpreted cautiously. Xu et al. (47) reported that films containing 1.79–5.38% XOS showed tensile strength of 42.7–50.7 MPa, low oxygen permeability of 4.95–5.06 cm^3^·μm/m^2^·day·kPa, and rapid release of approximately 92.6% of XOS in simulated gastric fluid within 60 min. These results demonstrate that XOS incorporation can be combined with acceptable mechanical and barrier performance and that the film matrix permits rapid XOS release under gastric simulation. However, this finding should be considered formulation-level evidence of release potential rather than direct evidence of colonic delivery, gut microbiota modulation, SCFA production, or nutritional benefit. Because most of the XOS was released during the simulated gastric phase, further studies are required to determine how much XOS remains available for intestinal and colonic fermentation, whether the amount delivered by a realistic edible coating reaches a physiologically meaningful dose, and whether fermentation by beneficial microbiota occurs under colonic conditions. Yeast biomass in BC/CMC/glycerol films further contributes nutritional and antimicrobial functions, particularly when applied to fruits such as oranges and tomatoes (84). Such multifunctional matrices combine structural reinforcement, bioactive delivery, and microbial protection, highlighting the versatility of edible films in complex food systems.

### 6.4 Matrix-governed release behavior and kinetic validation

Controlled release from edible polysaccharide-based interfaces should be interpreted not only in terms of total bioactive release, but also through the dominant transport mechanism. These mechanisms include Fickian diffusion, swelling-controlled release, pH-responsive solubilization, polymer relaxation or erosion, and mixed diffusion–relaxation behavior. The dominant release pathway is governed by interactions among the polymer network, the loaded bioactive compound, and the surrounding food or gastrointestinal medium, as well as by polymer composition, crosslinking density, hydrophilicity, film thickness, bioactive–polymer affinity, and the pH and polarity of the release environment.

In Fickian diffusion, the bioactive compound migrates from the film matrix to the surrounding medium along a concentration gradient. This mechanism is usually favored when the polymer network remains structurally stable during release and when the active compound can diffuse through water-filled pores or free-volume regions within the film. Dense and highly crosslinked matrices generally slow diffusion by reducing chain mobility and decreasing the available diffusion pathways. For example, EGCG incorporated into SA–CMC films was gradually released into 95% ethanol food simulant, maintaining antioxidant activity while mitigating lipid oxidation in fatty food matrices (103). Similarly, polyphenol release from chitosan films containing natural extracts was reported to follow quasi-Fickian diffusion behavior, indicating that diffusion through the hydrated polymer network was an important release pathway (121).

Swelling-controlled release occurs when water penetrates the polymer matrix, hydrates the chains, increases free volume, and facilitates the outward transport of encapsulated compounds. This mechanism is particularly relevant for hydrophilic polysaccharide films, including cellulose derivatives, hemicellulose-based films, alginate, starch, and chitosan blends. Swelling can accelerate release by opening the polymer network, but excessive swelling may also weaken mechanical integrity or cause rapid loss of active compounds. Plasticization, crosslinking, and nanofiller incorporation can therefore be used to regulate swelling and tune release rate. For example, XOS release from hemicellulose/chitosan/CNF films occurred rapidly in simulated gastric fluid while the films maintained tensile strength and low oxygen permeability, showing how hydration and network structure together control prebiotic release (47). Probiotic survival and release are also influenced by hydration, film thickness, storage temperature, and water activity, emphasizing the need to match matrix swelling behavior with the stability requirements of live microorganisms (120).

pH-responsive release is especially important for chitosan-containing edible interfaces because chitosan becomes protonated under acidic conditions. Protonation increases solubility, swelling, and electrostatic repulsion within the matrix, thereby enhancing the liberation of incorporated phenolics, flavonoids, essential oils, or prebiotic compounds. In contrast, at neutral or mildly alkaline pH, reduced protonation can decrease matrix solubility and slow release. Chitosan–polyphenol films therefore, showed greater bioactive liberation in acidic environments, where chitosan protonation promoted matrix hydration and solubilization (121). This pH-responsive behavior is relevant both during food storage, where local pH changes may accompany microbial spoilage, and during gastrointestinal exposure, where oral, gastric, and intestinal environments impose different release conditions.

In practice, most edible interfaces do not follow a single release mechanism. Instead, release is often governed by coupled diffusion–swelling behavior, followed by partial matrix relaxation, erosion, or pH-triggered solubilization. EO nanoemulsions in chitosan or guar gum matrices have been associated with improved antimicrobial or preservation outcomes during storage, but these outcomes should not be interpreted as direct evidence of slower release unless migration or release kinetics are measured (99, 102). Similarly, chitosan–CMC films containing blackberry anthocyanins and tea polyphenols improved chilled beef preservation, but the contribution of release-mediated activity should be distinguished from other effects such as matrix compactness, antioxidant capacity, and barrier behavior unless release is directly quantified (106).

**Figure 2:**
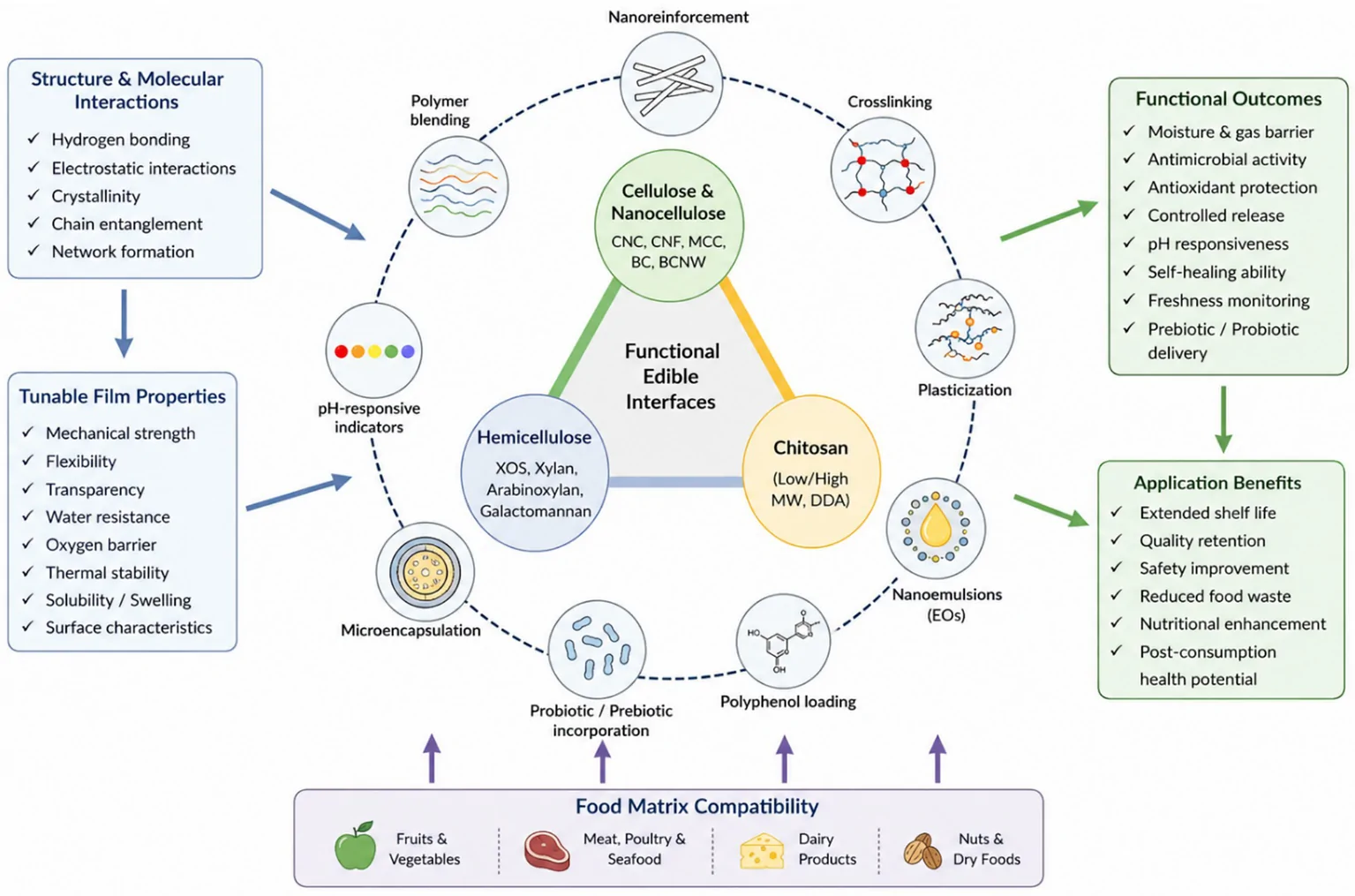
Structure function design map for cellulose, hemicellulose, and chitosan based edible interfaces.

Therefore, rational release design requires matching the polymer network and active cargo with the target food matrix or gastrointestinal environment. Rather than only reporting total release, future studies should fit release data to appropriate kinetic models, distinguish Fickian from anomalous transport, quantify swelling and erosion, and evaluate release under realistic food simulant and digestion conditions. Therefore, edible films should be described as potential mechanism-guided delivery matrices rather than as precision or programmable delivery systems unless their release profiles have been quantitatively fitted to appropriate kinetic models and validated under relevant food, simulant, or gastrointestinal conditions. The relationship among polymer selection, interpolymer interactions, engineering strategies, and active cargo loading is summarized in Figure 2, which provides a structure–function map for designing cellulose, hemicellulose, and chitosan-based edible interfaces with tailored preservation and delivery functions.

Hence, this evidence indicates that controlled delivery from edible interfaces should be evaluated as a kinetic and interaction-governed process rather than as a simple endpoint measurement of cumulative release. The key design question is not only how much bioactive compound is released, but which mechanism controls its release under a given food or gastrointestinal condition. Compact and highly crosslinked networks generally favor diffusion-limited release by increasing tortuosity and reducing chain mobility, whereas highly hydrated or weakly crosslinked matrices promote anomalous or swelling-controlled transport. Matrix erosion, enzymatic degradation, or pH-triggered solubilization may further shift the release mechanism, particularly in chitosan-containing systems exposed to acidic environments. At the same time, hydrogen bonding, electrostatic interactions, hydrophobic association, and physical entrapment determine whether the active compound remains bound within the matrix or partitions rapidly into the surrounding medium. Therefore, future studies should combine cumulative release profiles with kinetic model fitting, swelling and erosion measurements, matrix characterization, and real food or digestion-based validation. However, a critical limitation in the current edible film literature is that release mechanisms are often inferred from qualitative trends rather than demonstrated through quantitative kinetic fitting. Describing a release profile as Fickian, swelling-controlled, pH-responsive, relaxation-governed, or erosion-mediated requires more than reporting cumulative release. It requires fitting time-dependent release data to appropriate models, such as zero-order, first-order, Higuchi, Korsmeyer-Peppas, Peppas-Sahlin, Weibull, or diffusion-based models, and reporting parameters such as the release exponent, correlation coefficients, kinetic constants, and, when possible, diffusion coefficients. Recent studies have shown that model fitting can clarify whether release is governed mainly by diffusion, polymer swelling, matrix relaxation, or combined mechanisms; for example, food-packaging films have been evaluated using kinetic models such as Korsmeyer-Peppas, Peleg, and diffusion-based models in different food simulants, while alginate edible films have been studied as delivery systems for green tea polyphenols under gastrointestinal-relevant conditions (119, 122). Therefore, when kinetic modeling is absent, release behavior should be reported as an observed release trend rather than as proof of controlled, programmable, or precision delivery. This distinction is especially important for active edible interfaces because release in a food simulant, real food, or simulated digestion system may follow different mechanisms due to differences in pH, polarity, ionic strength, enzyme exposure, water activity, and matrix swelling. Accordingly, edible interfaces should be described as mechanism-guided delivery matrices rather than as precision or programmable delivery systems unless their release profiles are quantitatively modeled and validated under relevant food, simulant, or gastrointestinal conditions.

As summarized in Table 3, release validation across edible polysaccharide films remains highly uneven. Some studies quantified release under food simulant or simulated gastric conditions and fitted the data to kinetic or diffusion-based models, whereas others inferred delivery behavior from antimicrobial, antioxidant, or real-food preservation outcomes without measuring release directly. Therefore, terms such as “controlled release,” “pH-responsive release,” or “delivery system” should be used according to the level of evidence available in each study. When only cumulative release is reported, the result should be interpreted as release potential; when kinetic fitting and diffusion parameters are reported, the release mechanism can be discussed more confidently; and when only food preservation outcomes are measured, release-mediated functionality should remain a mechanistic hypothesis rather than a demonstrated result.

**Table 3:** Critical summary of release conditions and kinetic validation in representative edible polysaccharide-based delivery films.

System/matrix	Active compound and loading	Release test conditions	Release outcome	Kinetic validation	Matrix behavior/evidence level	Ref.
SA–CMC edible film	EGCG; 0.4, 0.8, 1.2, and 1.6 g added during film preparation; film thickness 0.12–0.14 mm	95% ethanol as fatty-food simulant; 30 × 20 mm film pieces in 30 mL simulant; 25 °C; dark; 0–168 h; HPLC analysis	EGCG release increased with loading. Films containing 0.8 and 1.2 g EGCG showed gradual release and stabilization, while 1.6 g showed higher and more extended release up to 168 h	No formal kinetic model fitted; release exponent and diffusion coefficient NR	Food simulant validation. Slow release was attributed to EGCG–SA/CMC interactions and compact morphology; mechanism remains qualitative without kinetic fitting	Ruan et al. (103)
Chitosan films with SOE/PBLE extracts	Polyphenols from *Sonneratia ovata* extract and *Piper betle* L. extract; chitosan:extract ratio 1:2 w/w; film thickness 0.050–0.075 mm depending on formulation	Distilled water, 3% acetic acid, 10% ethanol, and 50% ethanol; 3 × 3 cm films in 40 mL simulant; 27 °C; first 60 min and 24–72 h	At equilibrium, CS-PBLE2 released 31.74–34.94 mg GAE/g polymer; CS-SOE2 released 2.00–5.20 mg GAE/g polymer; CS-SOE1.4-PBLE0.6 released 13.32–15.32 mg GAE/g polymer. Highest release generally occurred in 3% acetic acid	Korsmeyer–Peppas and Peleg models fitted. Korsmeyer–Peppas n values were <0.5, indicating quasi-Fickian release; diffusion coefficient NR	Strong kinetic example. Release was strongly dependent on extract type, simulant, and chitosan solubility under acidic conditions; swelling/erosion not quantified	Thi Nguyen et al. (121)
Hemicellulose/pectin/nanocellulose biocomposite films	Mango peel polyphenols; experimental film thickness 40.05–66.12 μm; model input thickness 0.0064 cm	Fatty-food simulant; 30 mL simulant; model geometry 2 × 2 × 0.0064 cm; 5, 25, and 40 °C; 0–48 h	Polyphenol release increased with temperature and time, reaching up to 98.54%; release rate ranged from 0.014 ± 0.0004 to 0.021 ± 0.0017 mg/h; released amount ranged from 17.45 ± 0.451 to 25.61 ± 2.71 mg/kg simulant	Migratest EXP model based on Fick’s law; diffusion and partition coefficient framework used; exact numerical D values NR in available text	Strong model-based migration example. Film solubility in fatty simulant was reported; pectin and nanocellulose reduced solubility. Model accuracy was better in early release periods	Mugwagwa and Chimphango (119)
Hemicellulose/chitosan/CNF film	XOS, DP 2–6; XOS content: 1.79–7.18%; film thickness 31–34 μm depending on formulation	Simulated gastric fluid: 0.064 M HCl and 0.05 M KCl, pH 1.2; 10 mL medium; 37 °C; release assessed up to 60 min by HPAEC	For the F15% film, XOS content was 5.38 and 4.98% was released within 60 min, corresponding to approximately 92.6% release	No kinetic model fitted; release exponent and diffusion coefficient NR	Simulated gastric validation. Film began dissolving after 5 min and completely dissolved after 60 min. Demonstrates gastric release feasibility, not colonic delivery or proven prebiotic efficacy	Xu et al. (47)
CMC-based probiotic edible films	*L. acidophilus*, *L. rhamnosus*, and *B. bifidum* incorporated into CMC-based films; exact loading NR	Refrigerated storage system rather than release medium; 4 °C; 42 days	Viable counts remained above 10^7^ CFU/g over 42 days	Not a release kinetic study	Storage validation only. Film thickness, WVP, polymer concentration, and storage temperature affected probiotic survival, but digestion survival and release behavior were not evaluated	Ebrahimi et al. (120)
HPMC films with *Thymus daenensis* EO nanoemulsions	Wild or cultivated *T. daenensis* EO nanoemulsions; optimized film contained 2 mL nanoemulsion, 6 mL 20% HPMC, and 40 mg PEG 6000	No release medium; antimicrobial activity assessed by disk diffusion; incubation at 37 °C for 24 h	No cumulative release measured; antimicrobial activity reported as inhibition zones	No kinetic model fitted	In vitro antimicrobial validation, not release validation. Should not be used as evidence of controlled release unless EO migration is measured	Moghimi et al. (102)
Guar gum/chitosan film with orange EO nanoemulsion	Orange EO nanoemulsion; GG/CS film thickness 0.0550 ± 0.00453 mm; OEON films 0.0630–0.0713 mm; 4% OEON droplet size 380 ± 44.07 nm	Real cheese preservation; 4 °C; up to 12 days; no direct release medium	No cumulative release measured; preservation endpoints included pH, color, texture, microbial growth, and TBARS	No kinetic model fitted	Real-food preservation validation. Supports cheese preservation, but EO release was not quantified; should be interpreted as preservation evidence rather than kinetic release evidence	Cai et al. (99)
Chitosan–CMC film with BA/TP	Blackberry anthocyanins and tea polyphenols in CS/CMC films; thickness approximately 0.069–0.072 mm	Chilled beef preservation; 4 °C; 14 days; no independent release medium clearly reported	No cumulative release quantified; improved beef preservation by reducing pH, TVC, TBARS, and TVB-N and maintaining color/texture	No kinetic model fitted	Real-food preservation validation. Matrix compactness, ester bonds, amide bonds, and hydrogen bonding were discussed, but migration/release of BA or TP was not quantified	Chen et al. (106)

NR, not reported.

## 7 Matrix specific performance validation

The effectiveness of edible polysaccharide-based films is highly dependent on the specific characteristics of the food matrix. Structural, compositional, and physicochemical differences between fruits, vegetables, meats, dairy, and lipid-rich foods dictate the type of coating required for optimal preservation. While general film properties such as tensile strength, WVP, oxygen barrier performance, and antimicrobial activity are important, the match between film function and dominant spoilage pathways ultimately determines performance (84, 99, 111). To improve readability and avoid repetitive listing of individual applications, this section is organized according to the dominant deterioration pathways of each food category rather than by study-by-study description. The examples are therefore discussed as representative evidence for three major application logics: moisture and respiration control in fruits and vegetables, microbial and oxidative stabilization in animal-derived foods, and oxygen-barrier and controlled antioxidant release in dairy and lipid-rich matrices. This thematic organization highlights why a given edible interface works in a specific food system and how polymer selection, barrier regulation, active compound release, and sensory compatibility should be matched to matrix-specific preservation needs (see Table 4).

**Table 4:** Matching food matrices with dominant deterioration mechanisms and interface design priorities.

Food matrix	Dominant deterioration mechanisms	Required interface functions	Suitable polymer or active system	Representative validation endpoints	Ref.
Banana	Ripening, starch degradation, firmness loss, weight loss	Moisture regulation, delayed ripening, firmness retention	Citric acid crosslinked CMC films	Weight loss, firmness, color, ripening progression	Nongnual et al. (92)
Tomato	Respiration, weight loss, softening, color change	Selective barrier, partial coating, moisture control	Crosslinked starch plus CNF, BC/CMC/yeast films	Weight loss, firmness, color, sensory quality	Wardak et al. (85)
Strawberry	Fungal growth, moisture loss, texture deterioration	Antifungal action, transparency, barrier function	Chitosan/glycerol films, agar based CNC films, pH responsive hemicellulose films	Mold growth, firmness, color, freshness signal	Zhao et al. (88); Wang et al. (108); Pavinatto et al. (112)
Blueberry	Ripening, mold, yeast growth, moisture loss	Moisture barrier, microbial control, surface compatibility	HPMC based films, quinoa protein/chitosan/sunflower oil coating	Mold and yeast count, firmness, pH, color	Osorio et al. (109); Abugoch et al. (124)
Sweet cherry	Microbial growth, weight loss, acidity change	Antimicrobial coating, moisture control	Chitosan coatings	Weight loss, microbial count, acidity, soluble solids	Tokatlı and Demirdöven (111)
Fig	Softening, fungal contamination, bioactive loss	Gas modulation, firmness retention, bioactive preservation	Alginate/chitosan bilayer coating	CO2, ethylene, firmness, color, fungal contamination	Reyes-Avalos et al. (123); Reyes-Avalos et al. (134)
Beef	Lipid oxidation, microbial growth, color deterioration	Antioxidant delivery, antimicrobial protection, UV barrier	Chitosan/CMC with anthocyanins and tea polyphenols; chitosan flavonoid films	Lipid oxidation, microbial load, color, freshness	Chen et al. (106); Sutharsan et al. (117)
Chicken	Lipid oxidation, peroxide formation, microbial growth	Antioxidant release and barrier support	Cassava starch/CMC with apple polyphenols	Peroxide value, color, microbial stability	Lin et al. (97)
Sausage	Moisture loss, pH change, microbial growth, protein change	Moisture retention and microbial control	Modified BC/CMC/glycerol films	Moisture, pH, protein, microbial count	Yanti et al. (107)
Cheese	Lipid oxidation, microbial contamination, texture change	Oxygen barrier, hydrophobicity, antimicrobial release	Guar gum/chitosan with orange EO nanoemulsion	pH, color, texture, microbial growth, oxidation	Cai et al. (99)
Nuts and fatty foods	Rancidity, oxygen sensitivity, package damage	Antioxidant release, oxygen barrier, self-repair	Hemicellulose/pectin/nanocellulose with polyphenols; self-healing hemicellulose/chitosan microcapsule films	Oxidation, sensory quality, package integrity	Halloub et al. (118); Mugwagwa and Chimphango (119)

### 7.1 Fruits and vegetables

Fruits and vegetables require coatings that balance moisture and respiration control with antifungal protection and sensory preservation. Representative studies show that BC/CMC/yeast/glycerol films reduced weight loss and maintained sensory quality in oranges and tomatoes under refrigerated, room, and elevated temperatures, while citric acid-crosslinked CMC coatings delayed banana ripening by improving water resistance and oxygen-barrier behavior (84, 92). pH-responsive hemicellulose/PVA films with blueberry anthocyanin further illustrate how preservation and freshness monitoring can be integrated for strawberries (108). Chitosan-based systems add surface antimicrobial protection to this barrier function. Chitosan/glycerol coatings protected strawberries from gray mold, 1% chitosan reduced weight loss and suppressed yeast and mold growth in sweet cherries, and alginate-chitosan bilayers decreased CO2 production, improved firmness, and limited fungal contamination in figs (111, 112, 123). Together with protein/chitosan/oil coatings for blueberries, these examples indicate that fruit and vegetable interfaces should be selected according to respiration rate, surface moisture, susceptibility to fungal infection, and visual quality rather than as generic antimicrobial films (124).

Importantly, the physiological effects of edible coatings on fresh produce are not always beneficial in a linear manner. Although coatings can reduce water loss and slow gas exchange, excessively restrictive oxygen and carbon dioxide transfer may disturb normal respiration and ripening. If oxygen diffusion becomes too limited, fresh produce may shift toward anaerobic respiration, leading to ethanol or acetaldehyde accumulation, off-flavor formation, abnormal softening, tissue injury, or uneven ripening. Conversely, insufficient moisture control can accelerate wilting, shriveling, loss of turgor, and microbial susceptibility. Therefore, “better barrier performance” should not be treated as a universal design goal for fruits and vegetables. The optimal coating must provide selective gas and moisture regulation that matches the respiration rate, ethylene sensitivity, surface morphology, maturity stage, and dominant spoilage pathway of each commodity. This is particularly important for high-respiring or minimally processed produce, where coating thickness, lipid content, hydrophilic–hydrophobic balance, and storage temperature can strongly influence the internal atmosphere and sensory quality (3, 8, 125, 126).

### 7.2 Animal derived foods

Animal-derived foods require edible films that target microbial growth, lipid oxidation, moisture retention, and color stability. Chitosan/CMC films containing BA/TP maintained chilled beef quality for 14 days at 4 °C by inhibiting microbial growth and lipid oxidation while improving tensile strength and WVP, and cassava starch/CMC films with apple polyphenols preserved chicken fillets by lowering peroxide values and maintaining color (97, 106). These examples show that antioxidant and antimicrobial delivery is more important for meat and poultry than simple mechanical reinforcement. BC/CMC/glycerol films used for meat sausages maintained moisture, pH, protein content, and lower microbial counts, while chitosan films reinforced with flavonoids or pomegranate peel extract inhibited *E. coli*, *Listeria monocytogenes*, and *S. aureus* in beef and poultry systems (101, 107, 117). Collectively, these systems show that microbial suppression and oxidation control should be evaluated together because both determine color, texture, and shelf-life stability in animal-derived foods. For seafood and other high-lipid products, oxidative stability becomes especially critical. Hemicellulose/PVA/tea polyphenol films reduced lipid peroxidation and microbial growth in fish and shellfish, while chitosan-protein films with nanocrystalline cellulose and anthocyanins improved strength, water vapor barrier performance, and ammonia sensitivity for fresh fish (96, 127). Thus, films for meat and seafood should be tuned around oxygen control, antimicrobial release, antioxidant capacity, and matrix compatibility rather than described only by individual performance gains.

### 7.3 Dairy and high lipid matrices

Dairy products and lipid-rich foods require coatings that combine oxygen-barrier performance, low WVP, antioxidant release, and flavor compatibility. Guar gum/chitosan films with orange essential oil nanoemulsions improved hydrophobicity, water resistance, and elongation while reducing microbial growth and lipid oxidation in cheese (99). Hemicellulose/pectin/nanocellulose films containing mango peel polyphenols provide a complementary example, showing sustained antioxidant release into fatty simulants and tunable strength, solubility, and flexibility through control of the hemicellulose/pectin ratio (119). Canola residue-derived cellulose/hemicellulose/lignin films further demonstrate how residue-based systems can combine strength, low WVP, opacity, and biodegradation for oxygen-sensitive lipid-rich foods (128). Overall, dairy and lipid-rich matrices require less emphasis on broad antimicrobial listing and more focus on oxygen transfer, controlled antioxidant availability, moisture balance, and sensory compatibility.

### 7.4 Matching function to spoilage pathway

A fundamental principle in edible film design is that a single film formulation cannot address all food types equally. The primary spoilage pathways vary depending on the food matrix, necessitating targeted film engineering. For fruits and vegetables, preservation strategies must focus on respiration control, weight retention, firmness, and fungal inhibition, as demonstrated by BC/CMC/yeast/glycerol coatings on oranges and tomatoes (84) and citric acid-crosslinked CMC coatings on bananas (92). In these systems, the balance between barrier properties, polymer flexibility, and active antimicrobial agents ensures that moisture loss is minimized while spoilage organisms are suppressed. For meats and seafood, microbial growth, lipid oxidation, color retention, and protein degradation dominate. Chitosan/CMC films with polyphenols maintained beef quality, reduced lipid peroxidation, and inhibited pathogenic growth, while hemicellulose/PVA/tea polyphenol composites prevented oxidation in fish. Key film properties such as oxygen and WVP, mechanical integrity, and controlled release of bioactives directly influence the extent of microbial suppression and oxidative stability (96, 106).

In dairy and lipid-rich foods, oxygen and moisture barrier performance combined with antioxidant activity are crucial. GG/CS-OEON films effectively reduced moisture content and improved moisture barrier performance while providing antimicrobial protection in cheese (99). Hemicellulose/pectin/nanocellulose composites released polyphenols gradually, preserving lipid-rich foods against oxidative rancidity (119). Ultimately, the integration of structural reinforcement, barrier properties, gas and moisture permeability, and bioactive release must be tuned to the predominant spoilage mechanism and physiological behavior of each food type. In fresh produce, this means that coatings should reduce water loss and excessive respiration without creating an overly restrictive barrier that induces anaerobic metabolism, off-flavor formation, tissue damage, or abnormal ripening. At the polymer-design level, cellulose and nanocellulose mainly provide mechanical reinforcement and barrier modulation, hemicellulose and pectin can improve flexibility and release behavior in composite matrices, and chitosan contributes cationic and antimicrobial functionality (23, 25–27). In food applications, these functions must then be validated in the relevant matrix and storage condition, as illustrated by studies on fruits, vegetables, and cheese (84, 99, 111). This matrix-specific approach ensures maximal performance and demonstrates that edible films function not only as passive barriers but as precision-tailored active interfaces for each food category. To translate these food-specific findings into a practical design framework, Table 3 links representative food matrices with their dominant deterioration pathways, required interface functions, and suitable edible film strategies.

### 7.5 Condition-aware quantitative summary of reported performance

The quantitative performance of edible films and coatings is inherently context-dependent. Mechanical strength, WVP/WVTR, antimicrobial activity, release behavior, and shelf-life outcomes are influenced not only by polymer composition and active loading, but also by film thickness, conditioning relative humidity, test temperature, measurement units, control formulation, microbial strain, assay design, food matrix, storage conditions, and statistical treatment. Table 5 therefore summarizes representative systems alongside the experimental details reported in the original studies. This condition-aware format allows performance trends to be interpreted reliably while highlighting that values obtained under different protocols should not be directly compared. For release studies, the medium, temperature, film geometry, active loading, and kinetic modeling determine whether cumulative release can be mechanistically interpreted. Likewise, shelf-life extension depends on the dominant deterioration pathway, including moisture loss, respiration, microbial growth, lipid oxidation, enzymatic browning, or texture loss. These data should thus be viewed as formulation- and condition-dependent trends rather than a universal hierarchy of performance. Reporting complete experimental conditions and control values in future studies will enable more robust cross-study comparisons, meta-analyses, or standardized benchmarking.

**Table 5:** Illustrative quantitative summary of representative edible interfaces with reported test conditions for barrier performance, antimicrobial activity, release behavior, and shelf-life outcomes.

Performance category	Representative system	Experimental conditions	Control/reference	Reported outcome	Improvement/significance	Matrix/assay context	Ref.
Barrier/mechanical	Faba bean protein films reinforced with 1–7 wt% CNCs	Films prepared by casting; dried at 40 °C for 24 h; conditioned at 52% RH and 25 °C for 48 h; OTR measured at 23 °C and 50% RH; mechanical testing by ASTM D882-02	Control FBP film; TS ≈ 4.6 MPa	TS increased to ≈9.8 MPa; WVTR decreased by ≈30%; OTR also decreased	TS increased by ≈113%; WVTR decreased by ≈30%; statistical analysis reported in original study	Model edible film system; no food matrix; demonstrates reinforcement/barrier improvement under defined conditioning conditions	Rojas-Lema et al. (83)
Barrier/mechanical	HPMC films reinforced with MCC or lipid-coated MCC	HPMC films with 5–10% MCC; film thickness included in original WVP calculations; WVP reported as g·mm/kPa·h·m^2^	HPMC control; TS 35.6 ± 3.3 MPa; WVP 0.47 ± 0.04 g·mm/kPa·h·m^2^	MCC increased TS by 36–53%; WVP decreased up to ≈50% depending on formulation	Improvement reported relative to control; statistical grouping/CI reported in original study	Model film system; comparison should remain within same HPMC/MCC protocol because WVP depends on thickness and RH	Bilbao-Sáinz et al. (87)
Barrier/intelligent film	Hemicellulose/PVA films with blueberry anthocyanin and oxalic acid	Film thickness and RH/temperature not clearly extractable from provided file; WVP and OP reported with units	PVA or KHC/PVA control; WVP 15.48–17.92 × 10^−13^ g·cm/(cm^2^·s·Pa); OP baseline 13.22 × 10^−14^ cm^3^·μm/(m^2^·day·Pa)	WVP decreased to 7.27 × 10^−13^ g·cm/(cm^2^·s·Pa); OP decreased to 3.24 ± 0.98 × 10^−14^ cm^3^·μm/(m^2^·day·Pa); TS 36.64–41.24 MPa	WVP decreased by ≈53–59%; OP markedly reduced; statistical reporting present but exact *p*-values not fully extractable	Model intelligent film and strawberry freshness indication; barrier and colorimetric functions should be interpreted together	Wang et al. (108)
Antimicrobial/barrier	Tara gum films containing chitosan nanoparticles	Film thickness and RH/temperature not clearly extractable; antimicrobial tests against *E. coli* and *S. aureus*	Tara gum control; exact control values not fully extractable	Chitosan nanoparticles increased TS by 35.73 MPa, reduced elongation by 7.21%, reduced water solubility by 74.3%, and reduced WVP by 22.7%	Relative improvements reported; significance not clearly extractable from provided file	Model film system; antimicrobial performance depended on chitosan form and concentration; bulk chitosan showed stronger antimicrobial activity than nanoparticles	Antoniou et al. (105)
Antimicrobial/fruit preservation	1% chitosan coating on sweet cherries	Storage at 4 °C and 20 °C; 25-day refrigerated trial highlighted; coating thickness NR	Uncoated control; weight loss 16.18%; microbial count 2.74 log CFU/g	Coated cherries showed 8.85% weight loss; microbial counts remained below detectable level during 25 days at 4 °C	Weight loss reduced by ≈45%; microbial growth suppressed below detection; significance not clearly extractable	Real-food validation on sweet cherries; target microorganisms mainly yeasts/molds or natural spoilage flora	Tokatlı and Demirdöven (111)
Antimicrobial/active film	Hemicellulose-based films containing carvacrol; conventional and microemulsified forms	Contact angle and antimicrobial performance measured; RH/temperature not clearly extractable	Control contact angle 79.6°	Contact angle increased to 84.1° and 90.1° for 1 and 2% carvacrol C-films, and to 87.1° and 93.4° for M-films; >4 log reductions reported	Contact angle differences reported as significant; antimicrobial reduction >4 log	Model antimicrobial film/food-contact context; target microorganisms included *Listeria*, *E. coli*, and *Salmonella*	Hussain et al. (113)
Antioxidant/meat preservation	Chitosan–CMC films with blackberry anthocyanins and tea polyphenols	Chilled beef stored at 4°C for 14 days; film thickness ≈0.069–0.072 mm	CS/CMC film used as control	CS/CMC/BA/TP film showed higher TS and EAB, lower moisture content and WVP, and reduced pH, TVC, TBARS, and TVB-N in beef	Improvement reported relative to CS/CMC; statistical details not fully extractable	Real-food validation on chilled beef; antimicrobial, antioxidant, crosslinking, and barrier effects are coupled	Chen et al. (106)
Release/antioxidant	SA–CMC films containing EGCG	95% ethanol fatty-food simulant; 30 × 20 mm films in 30 mL simulant; 25 °C; dark; 0–168 h; HPLC analysis; film thickness 0.12–0.14 mm	EGCG-free or lower-loading films; control values NR	EGCG release increased with loading; 0.8 and 1.2 g films showed gradual release and stabilization; 1.6 g showed higher and extended release	Kinetic model not fitted; significance for release not fully extractable	Food simulant validation; supports gradual release potential but not full kinetic mechanism	Ruan et al. (103)
Release/antioxidant	Hemicellulose/pectin/nanocellulose films with mango peel polyphenols	Fatty-food simulant; 30 mL; 5, 25, and 40 °C; 0–48 h; model geometry 2 × 2 × 0.0064 cm; film thickness 40.05–66.12 μm	Control values NR	Polyphenol release reached up to 98.54%; release rate 0.014 ± 0.0004 to 0.021 ± 0.0017 mg/h; released amount 17.45 ± 0.451 to 25.61 ± 2.71 mg/kg simulant	ANOVA and Duncan test reported; diffusion/partition modeling applied	Strong food-simulant migration example; release modeled using Fickian diffusion-based Migratest EXP framework	Mugwagwa and Chimphango (119)
Release/prebiotic-loaded film	Hemicellulose/chitosan/CNF films containing XOS	Simulated gastric fluid, pH 1.2; 37 °C; 10 mL medium; release up to 60 min; film thickness 31–34 μm	Control without XOS; exact control release not applicable	XOS loading 1.79–7.18%; F15% film contained 5.38% XOS and released 4.98% within 60 min, equivalent to ≈92.6% release	No kinetic model fitted; significance not clearly extractable	Simulated gastric release only; demonstrates release feasibility, not colonic delivery or prebiotic efficacy	Xu et al. (47)
Probiotic carrier/storage	CMC-based probiotic films with *L. acidophilus*, *L. rhamnosus*, and *B. bifidum*	Refrigerated storage at 4 °C for 42 days; thickness varied with formulation but exact values not clearly extractable	Probiotic-free or formulation controls; exact values NR	Viable counts remained above 10^7^ CFU/g over 42 days	Statistical details not fully extractable	Storage validation; demonstrates probiotic survival during storage, not digestion survival or microbiota modulation	Ebrahimi et al. (120)
Antimicrobial/fruit coating	BC/CMC/Gly/yeast films	Films prepared with 30 wt% CMC, 30 wt% Gly, 2 wt% yeast, and 2 wt% BC; film thickness 0.17 ± 0.021 cm for yeast film; disc diffusion 24 h at 37 °C; fruit storage at 6 °C, 20–25 °C, 30 °C, and 40 °C	Negative control: pure BC or BC/CMC/Gly without yeast; positive control: yeast extract with inhibition zones of 14, 12, and 25 mm	BC/CMC/Gly/yeast produced inhibition zones of 16, 10, and 15 mm against *E. coli*, *P. aeruginosa*, and *S. aureus*; oranges acceptable up to 63 days at 6 °C and 28 days at room temperature/30 °C; tomatoes shelf life increased by 14 days at room temperature	Antimicrobial activity significantly higher than negative control; *p* < 0.05 and *p* < 0.01 reported	Combined antimicrobial, cytocompatibility, and real-food storage validation; sensory scoring/visual acceptability differs from microbial-count-based shelf-life studies	Atta et al. (84)
Shelf-life/cheese preservation	Guar gum/chitosan films with orange essential oil nanoemulsion	Cheese stored at 4°C up to 12 days; GG/CS film thickness 0.0550 ± 0.00453 mm; OEON films ≈0.0630–0.0713 mm; 4% OEON droplet size 380 ± 44.07 nm	GG/CS control; moisture content 96.86%; water solubility 72.27%; contact angle 59.9°	OEON films reduced moisture content to 34.69%, reduced water solubility to 69.76%, increased contact angle to 113.8°, and reached EAB of 135.12%; cheese pH, color, texture, microbial growth, and lipid oxidation better controlled	Moisture content decreased by ≈64%; contact angle increased by ≈90%; statistical details not fully extractable	Real-food cheese validation; essential-oil dose, odor, sensory acceptance, and release behavior remain important	Cai et al. (99)
Shelf-life/self-healing active film	UV-activated hemicellulose/chitosan film containing omega-3 microcapsules	Swelling tested in water at pH 7.04; other storage conditions not fully extractable	WOM film without microcapsules; swelling 444.76 ± 3 wt%; contact angle 98.6°	WM film with microcapsules showed swelling 443.67 ± 5 wt%; contact angle decreased to 70.1°; cashew preservation/shelf-life improvement reported	Swelling essentially unchanged; contact angle decreased by ≈29%	Lipid-rich food context; self-healing and microcapsule incorporation promising, but migration, oral exposure, and sensory effects need validation	Halloub et al. (118)

## 8 Consumption fate and microbiota relevance

Polysaccharide-based edible films are not limited to their preservative and barrier roles before consumption. If ingested, their fate should be evaluated under oral, gastric, intestinal, and, when relevant, colonic conditions, because hydration, swelling, dissolution, release behavior, enzymatic susceptibility, and microbial fermentability may differ substantially from storage behavior. Existing film studies mainly provide formulation-level or simulated gastric release evidence, such as the release of XOS from hemicellulose/chitosan/CNF films in simulated gastric fluid (73), whereas broader digestion and fermentation outcomes require dedicated gastrointestinal and colonic models. Therefore, this section distinguishes three levels of evidence: experimentally demonstrated outcomes, plausible mechanistic hypotheses, and unvalidated post-consumption claims. Experimentally demonstrated outcomes refer only to endpoints directly measured in the cited studies, such as release percentage, probiotic viability during storage, antioxidant retention, swelling, dissolution, degradation, antimicrobial activity, cytocompatibility, or food preservation performance. Plausible hypotheses include potential colonic exposure, fermentability, or microbiota interaction inferred from polymer chemistry or release behavior. In contrast, claims of prebiotic efficacy, synbiotic action, SCFA enhancement, microbiota modulation, or human nutritional benefit require dedicated validation using colonic fermentation models, microbiota sequencing, metabolomics, animal studies, or controlled human intervention trials.

### 8.1 Gastrointestinal exposure

Upon ingestion, edible films composed of cellulose, hemicellulose, or chitosan are exposed to sequential environments, including the oral cavity, stomach, and small intestine, where pH changes, enzymatic activity, and ionic composition influence hydration, swelling, and partial degradation. For example, XOS-containing hemicellulose/chitosan/CNF films released approximately 92.6% of XOS in simulated gastric fluid within 60 min, which demonstrates rapid release under acidic simulated gastric conditions rather than colonic delivery or prebiotic efficacy (47). Because most of the XOS was released during the gastric phase, this result does not by itself establish that a meaningful fraction of XOS remains protected until the colon, nor does it demonstrate that the dose delivered by an edible coating would be sufficient to induce measurable microbiota changes or SCFA production. Therefore, this system should be interpreted as a formulation with demonstrated XOS-loading and gastric-release capacity, but its colonic availability, fermentability, dose adequacy, and microbiota-modulating effects remain to be validated using sequential digestion–colonic fermentation models, microbiota profiling, metabolite/SCFA analysis, and eventually *in vivo* or human studies. Similarly, probiotics immobilized in CMC matrices, such as *Lactobacillus acidophilus* and *L. rhamnosus*, maintained viability during storage at 4 °C, indicating a validated carrier function during refrigerated storage, but not confirming probiotic efficacy after human consumption (120). The matrix composition governs swelling, solubility, and mechanical disintegration, which in turn modulate the release kinetics of bioactives. Crosslinked CMC films using citric acid exhibited enhanced water resistance and slower dissolution, enabling a more controlled release of incorporated antioxidants and oligosaccharides during digestion (92). In contrast, lightly reinforced hemicellulose films or films with lower crosslink density dissolved more rapidly, exposing prebiotics and bioactives to the gastrointestinal environment sooner.

### 8.2 Fermentability and SCFA potential

Cellulose and many hemicellulosic polysaccharides are not digested by human enzymes in the upper gastrointestinal tract, but fermentable dietary fiber fractions can be metabolized by colonic microbiota to produce short-chain fatty acids such as acetate, propionate, and butyrate (29, 30, 35). However, such dietary-fiber evidence should not be treated as direct proof that a specific edible film formulation delivers sufficient fermentable substrate to the colon. In the context of edible films and coatings, this possibility should be considered a mechanistic hypothesis rather than a demonstrated human outcome. The rate and extent of fermentation are expected to depend on polymer solubility, molecular weight, branching, crystallinity, chemical modification, and crosslinking density. Hemicellulose fractions, including xylans and arabinoxylans, are particularly relevant because their structural features can influence microbial accessibility and fermentability; for example, arabinoxylan structure and feruloylation have been shown to affect *in vitro* fermentability and gut microbiota modulation (28). However, because most of the XOS was released under simulated gastric conditions, this finding should be interpreted as evidence of gastric release potential rather than proof of colonic delivery or prebiotic efficacy. It remains unclear whether a sufficient fraction of XOS would reach the colon and whether the dose delivered by an edible coating would be high enough to induce measurable fermentation, SCFA production, or microbiota changes (47). Therefore, such release data do not directly demonstrate microbiota modulation in humans. Similarly, although cellulose nanofiber reinforcement can modify film structure, degradation, and release behavior, its specific effect on colonic fermentation kinetics remains insufficiently validated. Future studies should therefore combine simulated digestion with colonic fermentation models, SCFA quantification, microbiota sequencing, and ultimately human intervention studies before strong nutrition or microbiota-related claims are made.

### 8.3 Synbiotic interface concepts

Incorporating probiotics and prebiotic substrates into edible films may support the development of potential synbiotic interface concepts, but this classification should be used cautiously. Probiotic survival during refrigerated storage demonstrates carrier stability, not necessarily probiotic efficacy after ingestion. Similarly, probiotic survival during refrigerated storage and XOS release from edible films demonstrate carrier stability or prebiotic-substrate availability, but not confirmed synbiotic action after ingestion (47, 84, 120). A true synbiotic claim would require evidence that the probiotic remains viable through relevant gastrointestinal conditions, that the prebiotic fraction reaches the appropriate intestinal site at an effective dose, and that their combination produces measurable effects on microbial composition, metabolic activity, or SCFA production. Therefore, current probiotic- and XOS-containing edible films should be described as promising carrier systems for future synbiotic validation rather than as established synbiotic interfaces.

### 8.4 Chitosan and microbial selectivity

Chitosan, due to its cationic nature, can interact electrostatically with negatively charged bacterial membranes. While this imparts antimicrobial functionality in food preservation, its effects on gut microbiota are complex. Available evidence suggests that chitosan can inhibit several foodborne or pathogenic microorganisms, but its selectivity toward gut commensals remains insufficiently established in edible film systems (117, 129). Film processing parameters, including polymer molecular weight and drying temperature, further influence solubility, swelling, and enzymatic susceptibility, which collectively determine the degree and location of microbial interaction along the gastrointestinal tract (130, 131). The inclusion of crosslinkers, plasticizers, and reinforcing fibers may modulate chitosan solubility, charge exposure, and release behavior, but whether these changes selectively support probiotic colonization remains to be experimentally validated.

### 8.5 Evidence gaps in nutrition translation

The post-consumption relevance of edible films remains promising but insufficiently validated. Bioactive compounds such as polyphenols, anthocyanins, essential oils, probiotics, and prebiotic oligosaccharides can be incorporated into edible films and released under simulated food or gastrointestinal conditions (47, 97, 103). These studies are valuable because they demonstrate release potential, antioxidant retention, probiotic survival, or prebiotic availability. Accordingly, this review separates demonstrated formulation-level outcomes from post-consumption hypotheses. Demonstrated outcomes include bioactive release in food simulants or simulated gastrointestinal fluids, antioxidant retention, probiotic viability, swelling, degradation, dissolution, and food preservation endpoints. By contrast, gut microbiota restructuring, increased beneficial taxa, enhanced SCFA production, synbiotic efficacy, and human nutritional benefits remain provisional unless supported by dedicated colonic fermentation studies, microbiota sequencing, metabolomics, animal studies, or controlled human intervention trials. However, they should not be interpreted as direct evidence of human gut microbiota modulation or clinical nutritional benefit. Most available studies rely on indirect endpoints, including simulated gastric or intestinal release, food simulant behavior, *in vitro* antioxidant activity, antimicrobial assays, or survival of probiotic cells during refrigerated storage (47, 84, 106, 120). These endpoints are useful for screening formulation performance, but they cannot confirm whether edible films alter microbial diversity, increase beneficial taxa, enhance SCFA production, or improve host metabolic or immune outcomes after consumption. Human intervention studies are still largely absent, and the actual dose of film-forming polysaccharides, active compounds, probiotics, or prebiotics ingested through coated foods may be lower and more variable than doses used in conventional functional food or supplement studies.

To avoid overinterpretation, the evidence discussed in this section is distinguished according to its level of validation. Experimentally validated outcomes refer to endpoints directly measured in the reviewed studies, such as simulated gastrointestinal release, swelling or dissolution behavior, antioxidant activity in food simulants, probiotic survival during storage, antimicrobial activity, food quality preservation, or cytocompatibility. In contrast, gut microbiota modulation, SCFA enhancement, synbiotic efficacy, and human nutritional benefits are discussed as emerging or potential outcomes unless they were directly tested using colonic fermentation models, microbiota profiling, metabolomics, animal studies, or human intervention trials. This distinction is important because bioactive release, probiotic survival, or prebiotic availability under simulated conditions does not by itself demonstrate a measurable effect on the human gut microbiota or host nutrition.

Therefore, future research should adopt a staged validation framework. First, film digestion, swelling, degradation, and release behavior should be tested under standardized oral, gastric, intestinal, and colonic simulation conditions. Second, colonic fermentation models should be used to evaluate microbiota shifts, SCFA production, and metabolite profiles. Third, animal studies or controlled human trials should be conducted where justified, particularly for films designed to deliver probiotics, XOS, polyphenols, or fermentable hemicellulose fractions. Until such evidence is available, edible cellulose-, hemicellulose-, and chitosan-based interfaces should be described as systems with potential post-consumption relevance, rather than as proven modulators of the human gut microbiota.

## 9 Smart and adaptive edible interfaces

The evolution of edible films and coatings has progressed far beyond passive protection of food surfaces. Recent research highlights the development of intelligent and responsive interfaces, capable of sensing environmental changes, signaling freshness, or autonomously repairing minor mechanical damage. Such innovations expand the scope of edible films from passive packaging to multifunctional smart interfaces, integrating preservation, monitoring, and self-healing capabilities (108, 113, 118).

### 9.1 pH-responsive freshness indicators

pH-responsive films exploit the chemical interactions between polysaccharides, crosslinkers, and natural indicators to monitor food quality in real time. Hemicellulose-based PVA films, incorporating blueberry anthocyanins and oxalic acid crosslinkers, demonstrated reversible color changes corresponding to pH fluctuations, which were directly linked to fruit ripening and spoilage processes (108, 113, 118). In this system, esterification between hemicellulose and PVA modulates surface hydrophobicity and contact angle, enhancing barrier properties while allowing visual indication of freshness. Similarly, chitosan-based matrices can be combined with natural pH-sensitive pigments to produce films that signal microbial activity or spoilage via colorimetric changes (106). These indicators provide non-invasive monitoring, offering consumers and retailers an immediate assessment of product quality. By adjusting the concentration of anthocyanins, crosslinkers, and polymer ratios, the sensitivity and dynamic range of these responsive films can be tailored for different foods, including strawberries, blueberries, and beef.

### 9.2 Anthocyanin and phenolic colorimetric systems

Anthocyanins and other phenolic compounds act as both bioactive agents and visual indicators when incorporated into edible films. Films enriched with blackberry anthocyanins and tea polyphenols exhibited dual functionality: maintaining antioxidant activity while enabling colorimetric response to pH changes during beef storage (106). The interaction between anthocyanins and chitosan/CMC matrices forms hydrogen bonds and stabilizes the color response, allowing for sustained monitoring throughout the product’s shelf life. Additionally, these phenolic-based indicators have been successfully applied in hemicellulose/pectin/nanocellulose composites for fatty foods, where polyphenols not only enhanced antioxidant protection but also offered visual cues for oxidation progress (119). This multifunctionality represents a significant advance over conventional packaging, which only provides static protection without feedback on the food’s condition.

### 9.3 Self-healing and microcapsule repair

Mechanical damage to edible films, such as micro-tears or punctures, can compromise barrier performance and shelf-life. Self-healing strategies, inspired by natural repair mechanisms, have been implemented using microencapsulation of active agents. UV-activated microcapsules containing omega-3 oils embedded in hemicellulose/chitosan films can autonomously repair minor breaches in the polymer matrix when exposed to light, maintaining structural integrity and protecting cashew nuts from oxidation (118). The mechanism relies on microcapsule rupture, followed by diffusion and polymerization of the encapsulated oil under UV irradiation. This concept demonstrates that edible films can transition from static protective coatings to dynamic repair systems, enhancing food safety and nutrient retention. Similar approaches can be applied using lipid-based or protein-based microcapsules, providing a generalizable strategy for different food matrices.

### 9.4 Integrating active and intelligent functions

Intelligent edible interfaces aim to combine traditional preservation functions with monitoring or repair capabilities, but most reported systems remain proof-of-concept. For example, hemicellulose-based PVA films combining barrier performance, pH responsiveness, and colorimetric signaling have been evaluated for strawberry preservation and freshness monitoring, while UV-activated self-healing hemicellulose/chitosan films containing omega-3 microcapsules have been explored for lipid-rich foods (108, 118). The design of such systems requires careful consideration of polymer compatibility, crosslinking chemistry, bioactive incorporation, and environmental responsiveness. In selected studies, combining hemicellulose- or chitosan-based matrices with PVA, anthocyanins, or microcapsules has improved specific properties such as barrier performance, colorimetric responsiveness, or self-healing behavior, but these effects remain formulation-specific and should not be generalized across all smart edible films (108, 118). These systems illustrate a possible next step in edible film design by combining monitoring, responsiveness, and preservation functions, but their practical use still requires critical validation beyond (113). These systems illustrate a possible next step in edible film design by combining monitoring, responsiveness, and preservation functions, but their practical use still requires critical validation beyond laboratory-scale performance. However, these systems should still be regarded as emerging proof-of-concept technologies rather than mature edible packaging solutions. This distinction is particularly important for formulations containing PVA, anthocyanin indicators, crosslinkers, or self-healing microcapsules, because the edibility and regulatory acceptability of the final composite cannot be inferred from the polysaccharide component alone. For pH-responsive colorimetric systems, indicator performance must be calibrated against the specific pH range, buffering capacity, microbial ecology, and spoilage chemistry of each target food. Otherwise, color changes may generate false freshness signals, especially when pH shifts do not correlate directly with microbial load, lipid oxidation, texture loss, or sensory deterioration.

For self-healing microcapsule-based systems, additional safety and translation questions arise. Microcapsule rupture, migration of encapsulated oils or shell materials, UV-triggered reactions, residual crosslinkers, degradation products, and repeated oral exposure should be evaluated before these films are classified as edible in the practical sense. Sensory effects, including changes in color, gloss, odor, taste, mouthfeel, or consumer perception, are also critical because intelligent or self-repairing behavior may be technically successful but unacceptable for direct food contact or consumption. Therefore, smart and self-healing edible interfaces should be described as promising concept-driven systems whose practical application requires food-specific calibration, migration testing, oral-exposure assessment, microcapsule safety evaluation, sensory validation, and regulatory classification of the full formulation. Overall, smart and adaptive edible interfaces expand the role of polysaccharide-based films from passive protection to active quality management. pH responsive indicators, phenolic colorimetric systems, and self-healing microcapsule-based films demonstrate how preservation, monitoring, and structural recovery can be integrated within edible matrices. These issues define the main translational barriers for this emerging class of edible interfaces. These translational challenges are further discussed in the future perspectives section.

Despite these promising advances, the practical translation of smart and adaptive edible interfaces remains at an early stage and requires critical evaluation beyond proof-of-concept performance. Smart and adaptive edible interfaces represent a promising frontier in food preservation and bioactive delivery, integrating responsive functionalities such as pH-, temperature-, or enzyme-triggered release, self-healing, and visual quality indicators. While laboratory-scale studies demonstrate the potential of such systems to dynamically interact with food matrices and environmental cues, practical feasibility in real-world applications remains a significant challenge. Key limitations include the scalability of fabrication methods (e.g., layer-by-layer assembly, nanoparticle integration), variability in food surface properties, and the stability of functional additives under commercial storage and handling conditions.

Regulatory acceptance is another critical consideration. Intelligent edible systems often incorporate bioactive compounds, nanomaterials, or stimuli-responsive polymers. Compliance with food contact regulations (e.g., EFSA, FDA), assessment of potential migration, and long-term safety evaluation must be established before commercialization. Consumer safety also requires careful attention; this includes allergenicity of incorporated materials, cytotoxicity of nano-components, and potential interactions with existing dietary components.

Finally, commercial scalability depends not only on technical feasibility but also on economic viability. Cost-effective raw materials, reproducible processing, shelf-life consistency, and integration into existing food processing lines must be addressed. Current literature provides proof-of-concept demonstrations, but systematic evaluation of performance, safety, regulatory compliance, and cost is necessary to translate smart edible interfaces from research to market.

In summary, while smart and adaptive edible interfaces hold considerable promise, a holistic approach considering feasibility, regulatory frameworks, consumer safety, and industrial translation is essential. Future research should focus on integrating mechanistic design with scalable manufacturing, comprehensive toxicological evaluation, and real-food validation to ensure these intelligent systems can be adopted in practical applications.

## 10 Safety, acceptability, and translation

The transition of cellulose, hemicellulose, and chitosan based edible interfaces from laboratory formulations to food contact or directly consumable systems requires more than mechanical strength, barrier efficiency, or antimicrobial activity. For a film to be considered suitable for edible food applications, its components must be safe for oral exposure, compatible with food quality, acceptable to consumers, and feasible for industrial scale production. This requirement becomes more important when the matrix contains bioactive extracts, essential oils, probiotics, nanocellulose, crosslinkers, colorimetric indicators, or self-healing microcapsules. These ingredients may improve preservation performance, but they also introduce new questions related to cytotoxicity, sensory change, migration, degradation, release kinetics, and regulatory translation. Therefore, safety and acceptability should not be treated as final validation steps. They should be integrated into the design stage of edible interface development.

### 10.1 Edible exposure and cytocompatibility

Edibility is often assumed when polysaccharides such as cellulose derivatives, hemicellulose, chitosan, starch, gelatin, alginate, agar, carrageenan, or pectin are used. However, the safety profile of the final film depends on the full formulation rather than the base polymer alone. Plasticizers, crosslinkers, plant extracts, essential oils, nanoparticles, probiotic cells, yeast biomass, and encapsulated lipids can all alter the biological response of the material. For this reason, direct cytotoxicity and biocompatibility evaluation are particularly valuable in edible film studies.

Atta et al. (84) provided one of the clearest safety relevant examples by reporting that bacterial cellulose/carboxymethyl cellulose/glycerol films containing yeast biomass were non-toxic toward NIH-3 T3 fibroblast cells. This is important because the film was not only used as a surface coating for oranges and tomatoes but also designed as an edible bioactive system containing microbial biomass. In such cases, cytotoxicity testing helps distinguish between antimicrobial activity against foodborne microorganisms and undesirable toxicity toward mammalian cells. Similarly, Qi et al. (90) reported that chitosan/cellulose nanofiber films containing soybean protein isolate were non-toxic, while also improving antioxidant performance and water vapor barrier properties. These examples show that the inclusion of bioactive or reinforcing ingredients does not automatically compromise safety, provided that compatibility and dosage are properly optimized.

The food grade status of raw materials is equally important. Wang et al. (132) emphasized that chitosan and gelatin are attractive for edible packaging because food grade forms of these biopolymers are increasingly available and can be processed using casting, electrospinning, or thermoplastic methods. This point is central for regulatory translation because the same polymer may have different acceptability depending on purity, source, residual contaminants, degree of processing, and intended use. For example, chitosan obtained from crustacean waste may raise allergen related or source labeling considerations, whereas plant based cellulose and hemicellulose derivatives may be more broadly accepted in certain markets. Likewise, films derived from agricultural residues, such as corn husk cellulose nanocrystals, canola residue cellulose/hemicellulose/lignin, orange peel bacterial cellulose, or sugarcane bagasse hemicellulose, offer sustainability benefits but require careful control of extraction residues, batch variability, and compositional purity before food use (89, 91, 128, 133).

Nanocellulose also deserves specific attention. It is frequently used to reinforce edible films because of its high aspect ratio, crystallinity, and ability to reduce water vapor and oxygen transfer. Yet its safety evaluation should consider particle size, dispersion state, aggregation, and potential release from the matrix. Studies using cellulose nanocrystals or bacterial cellulose nanofibers generally focused on mechanical and barrier improvements, but their translation to edible use would benefit from additional digestion, migration, and cytocompatibility data (83, 86–88). The key point is not that nanocellulose is unsuitable, but that nanoscale reinforcement requires a clearer safety framework than conventional bulk polysaccharides.

Beyond nanocellulose, edible coatings containing nanofillers, nanoemulsions, concentrated plant extracts, essential oils, antimicrobial agents, colorimetric indicators, or encapsulated bioactives require formulation-specific regulatory and toxicological assessment. Their safety should not be inferred only from the food-grade status of the individual ingredients, because nanoscale dispersion, encapsulation, crosslinking, or controlled release can modify migration behavior, exposure dose, gastrointestinal availability, and biological response. Regulatory evaluation should therefore consider the intended food category, expected intake, compliance with food-contact and food-additive requirements, residual solvents or processing aids, degradation products, and possible migration of particles or active compounds into the food matrix during storage. Migration safety is particularly important for nanofiller-reinforced and nanoemulsion-loaded coatings because particle size, surface charge, hydrophobicity, interfacial stabilizers, and matrix binding strength can influence release and oral exposure. Allergenicity and source labeling also require attention, especially for chitosan derived from crustacean sources, protein-containing films, microbial biomass, and plant-derived extracts with strong bioactivity or aroma. Toxicological assessment should therefore combine cytocompatibility assays with standardized migration tests, digestion simulation, dose-dependent exposure studies, and, where necessary, evaluation of oxidative stress, inflammatory response, microbiota disturbance, or gastrointestinal irritation. This safety framework is essential for translating nanofiller-reinforced, nanoemulsion-loaded, and bioactive-rich edible coatings from laboratory formulations to consumer-ready food applications.

### 10.2 Sensory and visual acceptability

Consumer acceptance is strongly influenced by how an edible coating changes the appearance, odor, flavor, and mouthfeel of the food. For transparent fresh produce coatings, excessive opacity, discoloration, or surface roughness can reduce marketability even when preservation performance improves. For meat, cheese, or lipid rich foods, color stability and odor control are especially important because consumers often use these sensory cues to judge freshness.

Several studies in the dataset show that sensory acceptability must be balanced against functional performance. Atta et al. (84) reported that oranges and tomatoes coated with bacterial cellulose/carboxymethyl cellulose/glycerol/yeast films maintained acceptable odor and color under refrigeration, room temperature, and even elevated storage temperatures. This finding is valuable because yeast incorporation could potentially alter odor or appearance, yet the composite film preserved sensory quality for up to 2 weeks. In fresh figs, alginate/chitosan bilayer coatings maintained high sensory acceptability after storage, while also preserving firmness, external color, and bioactive compounds (123, 124, 134). Similarly, chitosan/glycerol films applied to strawberries protected against gray mold without producing meaningful negative changes in flavor, aroma, appearance, or texture (112).

Optical properties are another recurring issue. Rojas-Lema et al. (83) developed transparent faba bean protein films reinforced with cellulose nanocrystals, but increasing nanocrystal content can increase stiffness and alter optical clarity. Zhao et al. (88) reported agar-based composite films with light transmittance above 80% at 660 nm, which is favorable for strawberry packaging because consumers can still visually inspect the product. In contrast, some active films intentionally reduce light transmittance to protect foods against UV-induced oxidation. For example, Chen et al. (106) showed that chitosan/carboxymethyl cellulose films loaded with blackberry anthocyanins and tea polyphenols had lower UV–visible light transmittance, which improved beef preservation. Ruan et al. (103) also observed that epigallocatechin gallate deepened film color and decreased transmittance in sodium alginate/carboxymethyl cellulose films. These changes may be beneficial for oxidative protection, but they must be aligned with the food type. A darker antioxidant film may be acceptable for meat or fatty foods, but less desirable for fresh berries or minimally processed produce where visual clarity is critical.

Essential oils and plant extracts introduce additional sensory considerations. Moghimi et al. (102) showed that Thymus daenensis essential oil nanoemulsions provided strong antibacterial activity in hydroxypropyl methylcellulose films, but essential oils can also impart strong aroma or flavor. Cai et al. (99) incorporated orange essential oil nanoemulsions into guar gum/chitosan films for cheese preservation. In this case, the citrus derived active phase may be more acceptable in some dairy contexts than more pungent oils, but odor compatibility still requires product specific testing. Similarly, chitosan films containing pomegranate peel extract, Berberis crataegina extract, maqui berry extract, thyme extract, or plant ethanolic extracts improved antioxidant and antimicrobial functions, while also changing color, opacity, transparency, and sometimes thermal behavior (100, 101, 104, 135, 136). Therefore, sensory acceptability should be evaluated alongside active function rather than after formulation optimization.

### 10.3 Migration and release safety

Controlled release is one of the major strengths of edible interfaces, but it is also a safety consideration. When a film releases antimicrobial agents, antioxidants, essential oils, polyphenols, probiotics, XOS, or microencapsulated lipids, the release rate and total exposure level must be compatible with the food matrix and expected consumption pattern. Excessive release may alter taste, odor, color, or biological activity. Insufficient release may reduce functional efficacy. Ruan et al. (103) demonstrated slow release of epigallocatechin gallate from sodium alginate/carboxymethyl cellulose films into a fatty food simulant, together with strong antioxidant activity. This type of controlled release is desirable because it limits abrupt exposure while sustaining functionality. Thi Nguyen et al. (121) showed that polyphenol release from chitosan films containing *Piper betle* and Sonneratia ovata extracts was highest in acidic media and followed quasi Fickian diffusion behavior. This indicates that release is not fixed, but depends on pH, solvent environment, polymer solubility, and film swelling. Such behavior is highly relevant for foods with acidic surfaces and also for post-consumption exposure in gastric conditions.

Prebiotic release raises a different safety and functionality issue. Xu et al. (47) reported that hemicellulose/chitosan/cellulose nanofiber films containing xylooligosaccharides released about 92.6% of XOS in simulated gastric fluid within 60 min. This rapid release supports prebiotic availability, but it also means that dose control should be considered if films are consumed repeatedly. Likewise, probiotic loaded carboxymethyl cellulose films maintained viable populations of selected strains during refrigerated storage, but the films also showed higher WVP and opacity with reduced tensile strength compared with controls (120). The functional delivery of live microorganisms therefore has to be evaluated together with film stability, storage temperature, and intended intake.

For antimicrobial films, migration of active agents must be carefully optimized. Sayanjali et al. (137) showed that carboxymethyl cellulose films containing potassium sorbate inhibited mycotoxigenic fungi on pistachios, but higher sorbate levels reduced tensile strength and substantially increased WVP. This suggests that antimicrobial migration cannot be separated from the physical stability of the film. Similarly, carvacrol containing hemicellulose films showed strong antimicrobial reductions when microemulsification improved dispersion, but release related sensory and exposure profiles would still need to be evaluated for specific foods (113).

Degradation is also relevant for safety. Rajeswari et al. (138) and Arif et al. (128) highlighted biodegradability of polysaccharide based packaging films, which is positive from an environmental perspective. However, edible applications require an additional question: what degradation products are formed during storage, digestion, or thermal processing? Crosslinked systems, such as citric acid crosslinked carboxymethyl cellulose films or dialdehyde carboxymethyl cellulose crosslinked gelatin films, improve water resistance and barrier performance, but the residual crosslinking agent, reaction efficiency, and possible byproducts should be considered when moving toward edible exposure (92, 94). Self-healing hemicellulose/chitosan films containing omega 3 microcapsules add another layer of complexity because microcapsule rupture and UV triggered repair may change local release patterns and lipid oxidation behavior (118).

### 10.4 Scale up and regulatory translation

Industrial translation requires reproducible raw materials, scalable fabrication, food compatible processing, regulatory clarity, and cost effective production. Many studies rely on solution casting, which is suitable for laboratory development but may be difficult to scale without controlling drying time, film thickness, humidity, microbial contamination, and batch uniformity. Wang et al. (132) noted that chitosan and gelatin based films can be produced by casting, electrospinning, and thermoplastic methods, which suggests that multiple processing routes are available. However, each route introduces distinct regulatory and industrial challenges. Casting is simple but slow. Electrospinning enables high surface area structures but may require solvent control. Thermoplastic processing is more compatible with existing packaging infrastructure but may expose bioactives and probiotics to heat stress. Raw material standardization is another challenge. Agricultural residue derived materials support circular economy goals, as shown by bacterial cellulose from orange peels, cellulose nanocrystals from corn husks, hemicellulose from sugarcane bagasse, and cellulose/hemicellulose/lignin from canola residue (89, 91, 128, 133). Yet these sources can vary in composition depending on cultivar, season, extraction conditions, and purification efficiency. Regulatory translation will require specifications for purity, residual solvents, ash, protein residues, microbial load, heavy metals where relevant, and consistency in molecular weight or particle size.

Industrial translation also requires evaluation of storage stability, reproducibility, coating uniformity, processing-line compatibility, and consumer acceptance under realistic production conditions. Storage stability is particularly important because edible coatings may contain hydrophilic polymers, plasticizers, essential oils, probiotics, enzymes, anthocyanins, pH indicators, nanoemulsions, or microcapsules that can be sensitive to humidity, temperature, light, oxidation, microbial contamination, and time-dependent phase separation. Therefore, shelf-life studies should monitor not only coated food quality, but also the stability of the coating formulation before application, including viscosity, particle or droplet size, bioactive retention, microbial load, color stability, odor, and release behavior during storage. Reproducibility is another major challenge because natural polysaccharides and residue-derived polymers may vary in molecular weight, degree of deacetylation or substitution, crystallinity, ash content, protein residues, and impurity profile. These variations can affect film thickness, adhesion, drying behavior, mechanical strength, barrier performance, and active compound release. Coating uniformity must also be controlled during dipping, spraying, brushing, casting, extrusion, or continuous coating, because non-uniform layers may create local defects, uneven migration, inconsistent antimicrobial or antioxidant activity, and variable sensory perception. Compatibility with existing food-processing systems is equally critical; edible interfaces should tolerate commercial washing, cutting, drying, cooling, packaging, transport, and retail display without cracking, delamination, excessive tackiness, or loss of active function. Finally, consumer acceptance should be assessed early because edible coatings may modify gloss, transparency, color, odor, taste, mouthfeel, and perceptions of naturalness or safety. Therefore, successful commercialization requires moving beyond laboratory film preparation toward pilot-scale validation that integrates process reproducibility, coating uniformity, formulation stability, food-line compatibility, sensory testing, and consumer communication.

Regulation also differs depending on whether the film is considered packaging, a coating, a food ingredient, a carrier of active compounds, or an intelligent indicator. A simple chitosan or cellulose derivative coating may fit more easily into existing food contact or food ingredient categories. In contrast, films containing essential oil nanoemulsions, probiotic cells, XOS, anthocyanin indicators, potassium sorbate, carvacrol, or omega 3 microcapsules may require a more detailed safety dossier. The regulatory question is not only whether each ingredient is individually acceptable, but whether the final composite is safe under the intended conditions of use. This includes dose, migration, degradation, intended food, storage temperature, and consumption frequency. Another important translational issue is the boundary between edible and active or intelligent packaging. pH responsive hemicellulose/PVA films containing blueberry anthocyanin provide freshness monitoring, while self-healing hemicellulose/chitosan films with omega 3 microcapsules provide adaptive repair and preservation (108, 118). These systems are technologically advanced, but their regulatory pathway may be more complex than conventional edible films because they combine sensing, active release, and direct food contact. For consumer acceptance, the film must also remain understandable and trustworthy. A color changing edible film must avoid false freshness signals, and a self-healing film must not mask spoilage or delay visible deterioration beyond safe consumption limits. Because edible-interface formulations differ widely in composition and intended exposure, safety evaluation should be organized at the formulation-class level rather than only at the polymer level. Table 6 summarizes key regulatory and safety considerations for the major formulation classes discussed in this review. The matrix is not intended to assign regulatory approval status; rather, it identifies the evidence that should be verified for each class, including oral exposure, food-contact relevance, migration, allergenicity, cytotoxicity, expected dose, sensory limits, and remaining evidence gaps. This matrix highlights that the term “edible” should be applied to the final formulation under its intended conditions of use, not only to the dominant polymer. A cellulose-, hemicellulose-, or chitosan-based film may be built from broadly acceptable biopolymers, but the addition of crosslinkers, plasticizers, essential oils, phenolic extracts, nanoemulsions, nanofillers, probiotics, color indicators, or microcapsules can change migration behavior, oral exposure, sensory acceptability, and regulatory classification. Therefore, future studies should report not only film performance but also food-grade status of all components, residual processing aids, migration or release under relevant food simulants, estimated intake per serving, cytocompatibility, sensory thresholds, and formulation-specific evidence gaps.

**Table 6:** Regulatory and safety matrix for major edible-interface formulation classes.

Formulation class	Oral exposure/food-contact status	Main migration or exposure concerns	Allergenicity/cytotoxicity considerations	Dose and sensory limits	Current evidence gaps
Simple polysaccharide films and coatings, including cellulose derivatives, hemicellulose, chitosan, starch, alginate, pectin, agar, carrageenan, and gelatin	Often compatible with edible or food-contact use when food-grade materials are used; however, acceptability depends on purity, source, degree of processing, and intended application	Migration of low-molecular-weight fractions, plasticizer, residual salts, extraction residues, or processing aids; possible swelling or dissolution during storage or digestion	Source-related issues should be considered, especially crustacean-derived chitosan and animal-derived gelatin; cytotoxicity is usually low but should be verified for modified or composite systems	Dose is generally limited by coating thickness, texture, mouthfeel, gloss, and possible changes in taste or appearance	Need for clearer reporting of food-grade status, purity, residual contaminants, realistic intake, and digestion behavior
Plasticized and crosslinked films	Plasticizers such as glycerol or sorbitol may be acceptable when food-grade, but the full formulation must be assessed after crosslinking	Migration of plasticizers; residual crosslinkers; reaction byproducts; altered degradation products; changes in swelling and release behavior	Crosslinkers and reaction products may modify cytocompatibility; dose-dependent exposure should be considered	Excess plasticizer may increase tackiness, moisture uptake, off-texture, and WVP; crosslinking may increase stiffness or reduce edible mouthfeel	Limited data on residual crosslinker level, reaction efficiency, degradation products, and repeated oral exposure
Essential-oil-, phenolic-extract-, and organic-acid-loaded films	Some compounds may have food-use history or food-additive status, but approval cannot be assumed for the final film and dose	Migration or burst release of volatile oils, phenolics, organic acids, or antimicrobial agents; possible interaction with food components	Strong bioactivity may be associated with cytotoxicity at high dose; plant extracts may contain allergens, irritants, or compositionally variable compounds	Sensory limits are often decisive because odor, taste, bitterness, color, opacity, or astringency may restrict loading before toxicological limits are reached	Need for dose-per-serving estimation, extract standardization, migration testing, sensory thresholds, and real-food exposure assessment
Nano-reinforced films, including nanocellulose, chitosan nanoparticles, and other nanofillers	Food-contact suitability depends on particle type, size, surface chemistry, dispersion state, and regulatory jurisdiction; nanoscale status requires additional scrutiny	Potential release of particles or fragments from the matrix; aggregation/disaggregation during storage or digestion; altered gastrointestinal exposure	Cytotoxicity, oxidative stress, inflammatory response, and gut interaction should be evaluated, especially for free or weakly bound nanoparticles	Usually used at low loading, but sensory and texture effects may arise through opacity, roughness, stiffness, or altered mouthfeel	Need for migration studies, digestion simulation, particle-size tracking, chronic exposure assessment, and standardized nano-safety protocols
Nanoemulsion-loaded films	Food-contact status depends on the active oil, emulsifier, stabilizer, droplet size, and final use level	Migration of essential oil, surfactant, co-solvent, or interfacial stabilizer; enhanced bioavailability may increase exposure relative to bulk oil	Essential oils and emulsifiers may cause irritation, allergenicity, or cytotoxicity at high dose; droplet size may affect absorption	Sensory limits include strong aroma, flavor transfer, surface greasiness, opacity, or incompatibility with the target food	Need for droplet stability, release kinetics, migration, sensory threshold, and dose-per-serving data
Probiotic-, yeast-, or prebiotic-loaded films	Live microorganisms and prebiotics may be acceptable in foods, but strain identity, viability, dose, and intended use must be defined	Migration or release of cells, metabolites, nutrients, or prebiotic compounds; viability changes during storage; possible uncontrolled growth	Strain safety, absence of pathogenicity, antibiotic resistance, and host suitability should be considered; prebiotic dose should be physiologically meaningful	Dose per serving may be lower and more variable than conventional functional foods; sensory effects may include odor, turbidity, texture change, or fermentation notes	Need for strain-level safety, digestion survival, colonic fermentation, SCFA analysis, microbiota profiling, and human relevance
Colorimetric and intelligent indicator films, including anthocyanin-based pH-responsive systems	Edibility depends on the full indicator formulation, not only the pigment; PVA, crosslinkers, stabilizers, or solvents may complicate classification	Migration of pigments, stabilizers, solvents, or pH-sensitive components; possible false freshness signals if indicator response is not food-specific	Anthocyanins are often food-derived, but extract composition, stabilizers, and polymer matrix require evaluation	Color, staining, transparency, consumer interpretation, and possible taste or odor changes may limit use	Need for calibration against food-specific pH range, microbial load, volatile amines, oxidation markers, sensory spoilage, and migration behavior
Self-healing and microcapsule-based films	These should be treated as emerging proof-of-concept systems until oral exposure and food-contact classification of the full microcapsule formulation are established	Migration or rupture release of capsule core, shell materials, residual crosslinkers, UV-triggered reaction products, encapsulated oils, or degradation products	Microcapsule shell, core, and triggered release products may require cytotoxicity and digestion testing; repeated intake should be considered	Sensory limits include visible particles, gloss change, texture, mouthfeel, oil release, odor, or consumer concern about “self-healing” materials	Need for microcapsule safety, rupture behavior, digestion fate, migration, residual chemistry, sensory response, and regulatory classification
Agricultural-residue-derived films and extracts	Sustainability advantage is strong, but food-contact suitability depends on extraction method, purification, and compositional consistency	Residual solvents, acids, alkalis, lignin fragments, proteins, phenolics, pesticides, metals, or batch-variable impurities may migrate	Allergenicity and cytotoxicity depend on source material and purification; agricultural residues may vary by season and origin	Color, odor, opacity, and taste may be affected by residual lignin, phenolics, or pigments	Need for compositional standardization, contaminant screening, batch-to-batch reproducibility, and food-grade extraction validation

Overall, regulatory translation requires a design framework that integrates safety testing, migration assessment, sensory evaluation, scalability, and food specific performance. Future studies should report not only mechanical and antimicrobial outcomes, but also cytotoxicity, sensory panels, release profiles in relevant simulants, digestion behavior, residual processing aids, and stability during realistic storage. Edible interfaces will be more likely to reach practical use when their safety and acceptability are evaluated with the same rigor as their preservation performance.

**Figure 3:**
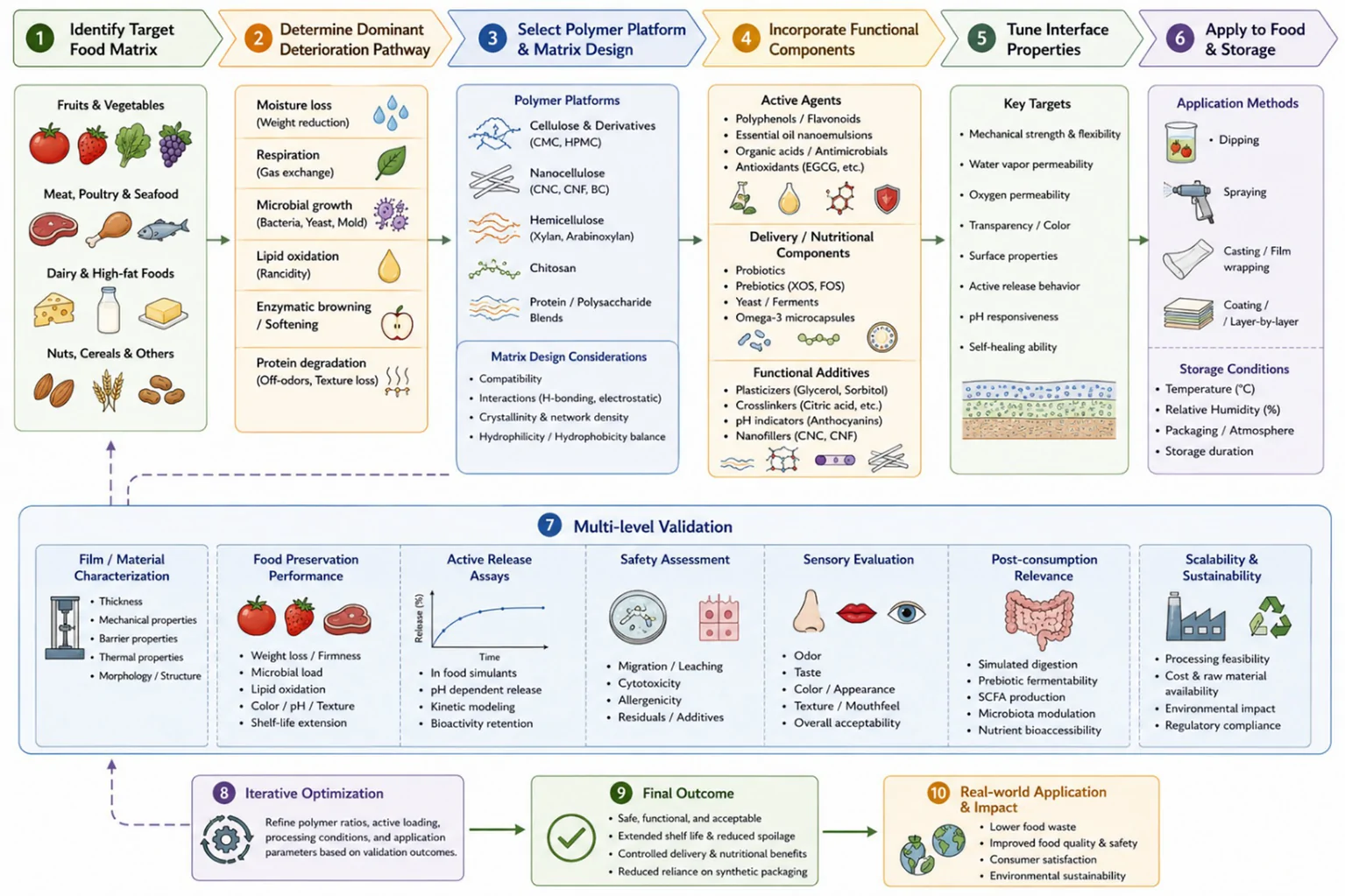
Food matrix specific design framework for next generation edible interfaces.

Building on the material, food matrix, delivery, safety, and translation considerations discussed above, Figure 3 proposes an integrated design framework in which edible interface development begins with the target food and its dominant deterioration pathway, followed by polymer selection, functional engineering, active cargo design, and multi-level validation.

## 11 Design rules for next generation interfaces

The studies reviewed in the previous sections show that edible films and coatings should no longer be designed as simple surface barriers. Their performance depends on a coordinated relationship among polymer chemistry, interpolymer interactions, bioactive loading, release behavior, target food matrix, sensory acceptability, and safety. A next generation edible food interface should therefore be designed through an integrated framework rather than by adding individual functional components in an empirical manner. The most effective systems are those in which the polymer platform, reinforcement strategy, active cargo, and food application are selected according to a defined preservation or nutrition-oriented objective.

### 11.1 Match polymer to function

The first design decision concerns the selection of the polymer platform. Cellulose derivatives, bacterial cellulose, hemicellulose, chitosan, starch, alginate, gelatin, proteins, and gums each provide different advantages, and their suitability depends on the dominant function expected from the interface. Cellulose based systems are particularly useful when mechanical strength, transparency, and barrier reinforcement are required. For example, hydroxypropyl methylcellulose films reinforced with microcrystalline cellulose nanoparticles showed improved tensile strength and moisture barrier performance, indicating that cellulose derivatives can be strengthened without losing their basic film forming properties (87). Similarly, faba bean protein films reinforced with cellulose nanocrystals gained improved tensile strength, thermal stability, hydrophobicity, and reduced water vapor and oxygen transmission, making them suitable for packaging applications requiring structural stability and barrier function (83).

Bacterial cellulose provides another route when a nanofibrillar scaffold is needed. Bacterial cellulose based films containing carboxymethyl cellulose and glycerol maintained sausage quality during storage, while bacterial cellulose/carboxymethyl cellulose/glycerol films containing yeast biomass preserved oranges and tomatoes under different temperature conditions (84, 107). These examples suggest that bacterial cellulose is valuable when a hydrated yet mechanically coherent network is required for food surface protection.

Hemicellulose based films are more relevant when flexibility, renewability, biodegradability, and prebiotic potential are desired. Hemicellulose/chitosan films reinforced with cellulose nanofibers showed improved tensile strength and oxygen barrier behavior, while XOS containing hemicellulose/chitosan films introduced a direct prebiotic dimension (47). Chitosan should be prioritized when antimicrobial activity, cationic interactions, and bioactive compatibility are central to the design. Chitosan coatings protected strawberries, sweet cherries, beef, and composite fruit systems through microbial inhibition and barrier regulation (112, 117, 131). Therefore, polymer selection should follow the intended function. Mechanical reinforcement, gas control, antimicrobial activity, prebiotic delivery, and intelligent monitoring require different starting matrices.

### 11.2 Engineering the network

A recurring lesson from the reviewed studies is that performance improvement is not simply a matter of adding more filler, extract, or nanocellulose. The most successful films are those in which molecular interactions among the components are deliberately engineered. Hydrogen bonding, electrostatic attraction, esterification, Maillard reactions, and physical entanglement determine whether the film becomes compact and functional or heterogeneous and unstable. Protein and polysaccharide combinations illustrate this principle clearly. In whey protein films reinforced with bacterial cellulose nanowhiskers, the nanowhiskers were homogeneously distributed in the protein matrix and increased tensile strength, Young’s modulus, and water vapor resistance (89). Chitosan/cellulose nanofiber films containing soybean protein isolate performed best at 1% SPI, where electrostatic attraction and hydrogen bonding improved compatibility, reduced WVP, increased contact angle, and enhanced antioxidant capacity (90). This shows that an optimal concentration can outperform higher loadings if it promotes better network organization.

Crosslinking strategies also demonstrate the importance of controlled interactions. Citric acid crosslinked carboxymethyl cellulose films improved water resistance and preserved banana quality, while Maillard reaction based carboxymethyl cellulose/soy protein isolate films showed improved mechanical properties and reduced water sensitivity (92, 93). Dialdehyde carboxymethyl cellulose crosslinked gelatin films increased tensile strength, thermal stability, UV barrier capacity, and reduced swelling and WVP (94). In these cases, the crosslinker did not merely act as an additive. It reorganized the polymer network. The same principle applies to nanofillers. In *Althaea rosea* gum films, bacterial nanocrystalline cellulose improved properties at optimal loading, but excessive addition caused agglomeration and impaired some functions (98). In konjac glucomannan films, bacterial cellulose nanofibers improved crystallinity, thermal stability, and barrier properties, but the balance between tensile strength and elongation depended on nanofiber content (86). Thus, rational design should focus on interfacial compatibility, dispersion quality, and network architecture rather than filler concentration alone.

### 11.3 Match cargo to release environment

Bioactive loading should be guided by the environment in which release is expected to occur. Antioxidants, antimicrobials, probiotics, prebiotics, essential oils, and freshness indicators do not require the same release profile. Some compounds must act on the food surface during storage, while others should remain protected until exposure to food simulants or gastrointestinal fluids.

Phenolic compounds are appropriate for lipid rich foods and meats when sustained antioxidant release is required. Sodium alginate/carboxymethyl cellulose films containing epigallocatechin gallate released EGCG slowly in a fatty food simulant and showed strong radical scavenging activity, supporting their use in oxidative protection of fatty foods (103). Hemicellulose/pectin/nanocellulose biocomposites containing mango peel polyphenols also provided slow release into fatty simulants and increased antioxidant activity, showing that matrix composition can regulate release into lipid like environments (119). Chitosan/carboxymethyl cellulose films containing blackberry anthocyanins and tea polyphenols were more suitable for chilled beef because they combined antioxidant, UV blocking, and antimicrobial functions with improved film strength (106).

Essential oils require a different strategy. Their volatility and hydrophobicity make nanoemulsion systems more appropriate than direct addition. Hydroxypropyl methylcellulose films containing Thymus daenensis essential oil nanoemulsions showed strong antimicrobial activity, while guar gum/chitosan films containing orange essential oil nanoemulsions improved cheese preservation by controlling microbial growth and lipid oxidation (99, 102). In these cases, nanoemulsion loading helped disperse the active phase and reduce uncontrolled release. For nutrition-oriented interfaces, the release environment is even more important. Xylooligosaccharides in hemicellulose/chitosan/cellulose nanofiber films were released rapidly in simulated gastric fluid, supporting their role as prebiotic cargo (47). Probiotic loaded carboxymethyl cellulose films preserved selected strains at refrigerated temperature but lost viability more rapidly at 25 °C, indicating that storage temperature and matrix water activity must be considered for microbial delivery (120). Therefore, the bioactive cargo should be selected together with its intended release site, release rate, and stability requirements.

### 11.4 Match food to spoilage pathway

A central design rule is that edible interfaces must be matched to the dominant deterioration mechanism of the food. Fruits and vegetables usually require control of respiration, water loss, firmness, color, and fungal growth. Animal derived foods require stronger attention to microbial load, lipid oxidation, protein deterioration, and color stability. Dairy and nuts require oxygen control, moisture regulation, and protection against rancidity. For fruits, coatings must be flexible, transparent, and able to regulate moisture and gas exchange. Citric acid crosslinked carboxymethyl cellulose films preserved bananas by reducing weight loss, delaying ripening, maintaining firmness, and retaining color (92). Partial coatings based on crosslinked starch and cellulose nanofibers reduced weight loss and maintained tomato firmness and color, while requiring less total coating coverage than full coatings (85). Chitosan coatings protected strawberries against gray mold and sweet cherries against microbial proliferation and weight loss (111, 112). Alginate/chitosan bilayer films preserved figs by maintaining firmness, reducing fungal contamination, and preserving bioactive compounds (123, 134).

For meat and poultry, antioxidant and antimicrobial functions must be prioritized. Apple polyphenol containing cassava starch/carboxymethyl cellulose films reduced peroxide values and supported chicken preservation (97). Chitosan films containing flavonoids improved chilled beef quality by maintaining color and suppressing microbial growth (117). Chitosan/carboxymethyl cellulose films with blackberry anthocyanins and tea polyphenols also maintained chilled beef quality through combined barrier, antioxidant, and antimicrobial effects (106). For cheese, guar gum/chitosan films containing orange essential oil nanoemulsions were more suitable because they addressed hydrolysis, microbial contamination, lipid oxidation, moisture change, and texture variation (99). For cashew nuts, self-healing hemicellulose/chitosan films with omega 3 microcapsules were relevant because lipid oxidation and packaging damage are key risks during storage and handling (118).

These examples confirm that there is no universal edible film formulation. A coating optimized for strawberries may not be appropriate for beef, and a film suitable for fatty foods may not be acceptable for transparent fruit packaging. The design should begin with the food matrix and its deterioration pathway, not with the material alone.

### 11.5 Validate as an integrated system

The final guideline is to validate edible interfaces as integrated systems. A film that shows high tensile strength but poor sensory acceptability, strong antimicrobial activity but excessive migration, or good antioxidant release but weak safety evidence is not ready for translation. Future studies should therefore combine preservation tests with release studies, sensory analysis, cytotoxicity, digestion simulation, and application in real foods. Some studies already move in this direction. Atta et al. (84) combined antimicrobial testing, cytotoxicity evaluation, and real food application on oranges and tomatoes. Qi et al. (90) integrated structural analysis, antioxidant assays, barrier testing, and toxicity assessment in chitosan/cellulose nanofiber/soy protein films. Chen et al. (106) linked chemical interactions, mechanical and barrier properties, active compound incorporation, and chilled beef preservation. Xu et al. (47) connected mechanical reinforcement with XOS release, introducing a bridge between packaging performance and prebiotic functionality.

Nevertheless, many studies still focus heavily on film characterization while giving limited attention to digestion behavior, sensory response, migration safety, or human nutrition relevance. For next generation edible interfaces, validation should include four linked levels. First, the film must perform technically, with adequate strength, flexibility, barrier function, and stability. Second, it must preserve the target food under realistic storage conditions. Third, it must be safe and acceptable, with controlled release, limited undesirable sensory effects, and evidence of cytocompatibility when new bioactive or nanoscale components are used. Fourth, if nutrition claims are implied, digestion, bioaccessibility, prebiotic activity, probiotic survival, or microbiota related outcomes should be examined. In this framework, edible food interfaces become more than biodegradable coatings. They become designed systems that connect material science, food preservation, active delivery, safety, and nutrition. This integrated approach is essential for moving the field from promising laboratory formulations toward practical, regulatorily acceptable, and consumer-relevant food technologies.

## 12 Limitations and unresolved evidence gaps

Despite rapid progress in cellulose-, hemicellulose-, and chitosan-based edible interfaces, several unresolved limitations still restrict direct comparison, mechanistic interpretation, and practical translation of the reported systems. A major challenge is the lack of standardized testing protocols. Mechanical strength, elongation, WVP, WVTR, oxygen permeability, antimicrobial activity, release behavior, and shelf-life outcomes are frequently measured under different film thicknesses, conditioning relative humidities, temperatures, units, microbial strains, food matrices, active loadings, and storage conditions. Consequently, numerical values reported across studies should be interpreted as condition-dependent trends rather than directly comparable performance rankings. The translation of formulation-level results into real-food efficacy also remains limited. Many edible films are evaluated mainly through material characterization, inhibition-zone assays, antioxidant assays, release in food simulants, or short-term storage tests. These endpoints are useful for screening, but they do not necessarily demonstrate shelf-life extension in complex foods. Real-food performance depends on food composition, pH, water activity, fat content, surface roughness, respiration rate, microbial load, storage temperature, coating adhesion, and release kinetics. Stronger validation should therefore connect film composition and mechanism with food-level outcomes such as microbial counts, lipid oxidation, pH, firmness, color, texture, odor, sensory acceptability, and consumer-relevant shelf life. Sensory and consumer-related evidence is another underdeveloped area. Edible films may modify gloss, opacity, color, odor, taste, texture, tackiness, and mouthfeel, yet many studies prioritize mechanical, barrier, or antimicrobial performance without defining sensory thresholds or consumer perception. This limitation is particularly important for systems containing essential oils, phenolic extracts, nanoemulsions, probiotics, color indicators, or microcapsules, where technical functionality may be achieved at concentrations that are not acceptable for direct food contact or consumption.

Oral-exposure safety and regulatory readiness also require more formulation-specific evaluation. Although cellulose, hemicellulose, chitosan, starch, alginate, pectin, gelatin, and other biopolymers may have food-contact or food-use relevance, the safety of the final formulation cannot be inferred from the base polymer alone. Plasticizers, crosslinkers, essential oils, phenolic extracts, nanoemulsions, nanocellulose, nanoparticles, probiotics, color indicators, microcapsules, residual solvents, degradation products, and processing aids may alter migration behavior, cytotoxicity, allergenicity, sensory limits, dose per serving, and regulatory classification. More complete reporting of food-grade status, residual chemistry, migration into relevant simulants or foods, repeated-exposure estimates, cytocompatibility, digestion behavior, and sensory limits is therefore needed. Evidence for gut microbiota modulation and post-consumption benefit remains especially weak and mostly indirect. Release in simulated gastric fluid, antioxidant activity, probiotic survival during storage, or prebiotic incorporation demonstrates formulation feasibility, but it does not prove colonic delivery, fermentation, SCFA production, microbiota restructuring, or human nutritional benefit. Claims related to prebiotic, synbiotic, microbiota-oriented, or post-consumption functionality should be supported by dose-relevance analysis, simulated intestinal and colonic digestion, fermentation models, microbiota profiling, metabolomics, animal studies, and ultimately controlled human intervention trials. Industrial translation is further constrained by the limited number of pilot-scale and process-oriented studies. Most edible films are still prepared by laboratory-scale casting, whereas commercial application requires reproducible coating, spraying, dipping, extrusion, or continuous film-forming processes. Scale-up may change film thickness, drying kinetics, coating uniformity, active-compound stability, microbial safety, cost, and compatibility with existing washing, cutting, drying, cooling, packaging, transport, and retail systems. Future work should therefore address pilot-scale validation, batch-to-batch reproducibility, raw-material variability, process energy demand, coating uniformity, storage stability, and techno-economic feasibility. Addressing these limitations is essential for moving edible interfaces from promising laboratory prototypes toward safe, standardized, scalable, and consumer-acceptable food technologies.

## 13 Future perspectives

The development of cellulose-, hemicellulose-, and chitosan-based edible food interfaces is shifting from simple preservation coatings toward integrated systems that combine material performance, active delivery, food safety, sustainability, and cautiously defined nutrition-oriented potential. The studies discussed throughout this review show clear progress in mechanical reinforcement, antimicrobial protection, antioxidant release, shelf life extension, freshness monitoring, and self-healing behavior. However, the field still requires a more coordinated design framework to move from promising laboratory formulations to reliable, scalable, and acceptable food technologies. In light of these unresolved limitations, future research should move from empirical formulation development toward mechanism-guided, standardized, and application-specific edible interface design. The next stage of the field should prioritize integrated validation frameworks that connect polymer structure, food matrix behavior, active release, safety, sensory acceptability, regulatory readiness, and scalable processing.

Emerging technologies for next-generation edible interfaces should be considered as tools for solving these unresolved gaps rather than as isolated technological trends. AI-assisted material design can support the prediction of polymer compatibility, nanofiller dispersion, barrier performance, release kinetics, sensory attributes, and shelf-life outcomes by integrating formulation descriptors with experimental datasets. However, its application will require standardized reporting, sufficiently large and reliable datasets, interpretable models, and experimental validation to avoid overfitting or purely data-driven conclusions without food-matrix relevance. Stimuli-responsive delivery systems represent another important direction, particularly for coatings that release antimicrobials, antioxidants, probiotics, or prebiotics in response to pH, enzymatic activity, humidity, oxidation, or microbial metabolites. Their promise lies in matching release to spoilage or digestion conditions, but critical challenges remain in calibration, dose control, migration safety, regulatory acceptance, and sensory neutrality. Bioinspired interfaces, including multilayer structures inspired by plant cuticles, mucosal adhesion, shell-like barrier architectures, or antimicrobial peptides, may provide new strategies for combining adhesion, flexibility, selective permeability, and active protection. Nevertheless, these systems must be simplified into food-grade, scalable, and cost-effective formulations. Additive manufacturing and 3D-printed edible coatings may further enable spatial control over layer thickness, porosity, texture, and localized bioactive distribution, but their translation depends on printable food-grade inks, drying behavior, processing speed, equipment compatibility, and consumer acceptance. Finally, precision nutrition-oriented edible packaging could link preservation with individualized delivery of fibers, polyphenols, probiotics, or prebiotics. At present, this concept remains largely prospective because robust evidence connecting edible interface ingestion with microbiota changes, metabolite production, and human outcomes is still limited. Therefore, future progress should combine AI-guided formulation, stimuli-responsive release, bioinspired design, advanced manufacturing, and nutrition-oriented validation within a cautious framework that prioritizes safety, regulatory readiness, sensory quality, scalability, and evidence-based claims.

A key future direction is the transition from empirical formulation to mechanism-guided interface design. Many edible films are still optimized by adjusting filler concentration, polymer ratio, plasticizer level, or bioactive loading through trial and error. Although this approach can improve performance, it does not always explain why a formulation works or why it fails. Future research should place greater emphasis on polymer compatibility, hydrogen bonding, electrostatic interactions, crosslinking density, crystallinity, swelling behavior, surface charge, and network morphology. These parameters directly influence tensile strength, flexibility, WVP, oxygen transfer, transparency, release behavior, and sensory quality. A stronger mechanistic understanding would make it easier to design films for specific foods rather than repeatedly screening broad formulation libraries.

Food specific validation is another priority. Edible interfaces cannot be evaluated only through general film properties such as tensile strength, elongation at break, WVP, or antioxidant activity. These parameters are useful, but they do not fully predict performance on real food surfaces. Fruits, vegetables, meats, dairy products, nuts, and fatty foods deteriorate through different pathways. Fruits generally require control of respiration, water loss, firmness, color, and fungal growth. Meat, poultry, and seafood require stronger control of microbial load, lipid oxidation, protein degradation, and color stability. Dairy and lipid rich foods need oxygen barrier performance, moisture regulation, and sustained antioxidant action. Future studies should therefore test coatings under realistic storage conditions and use food relevant endpoints, including weight loss, firmness, microbial count, lipid oxidation, color, pH, texture, sensory quality, and consumer visible changes.

Sustainability claims also require more rigorous life-cycle and techno-economic evaluation. Although cellulose, hemicellulose, and chitosan are renewable, biodegradable, and often obtainable from agricultural or food-processing residues, their large-scale implementation is not automatically environmentally superior to conventional packaging. Life-cycle assessment should consider raw material sourcing, extraction yield, purification requirements, solvent and water consumption, drying energy, coating application method, transportation, storage stability, end-of-life fate, and the effect of the coating on food waste reduction. Economic feasibility should also be evaluated beyond laboratory material cost, including polymer recovery or purchase price, batch-to-batch variability, food-grade purification, drying time, process throughput, equipment compatibility, quality control, and regulatory testing. Industrial processing may introduce additional constraints because solution casting, which is widely used at laboratory scale, may not translate directly to continuous coating, spraying, dipping, extrusion, or roll-to-roll processing. Environmental trade-offs should therefore be explicitly assessed: for example, a biodegradable edible coating may reduce plastic waste but require high water or energy input during extraction and drying; a highly active formulation may extend shelf life but increase cost, sensory impact, or regulatory complexity; and residue-derived polymers may support circular bioeconomy goals but require purification to remove contaminants and ensure compositional consistency. Therefore, future research should combine performance testing with life-cycle assessment, preliminary techno-economic analysis, and scalable processing studies to identify formulations that are not only functional and biodegradable, but also economically realistic and environmentally justified at industrial scale.

Controlled release also requires deeper investigation. Polyphenols, flavonoids, anthocyanins, essential oils, probiotics, yeast biomass, prebiotic oligosaccharides, antimicrobial agents, and lipid microcapsules behave differently inside polysaccharide matrices. Their release depends on pH, water activity, solvent polarity, film swelling, polymer solubility, crosslinking, and matrix compactness. Future studies should connect release kinetics to actual food quality or biological outcomes. For example, antioxidant release should be linked to lipid oxidation control, antimicrobial release to microbial reduction, indicator response to spoilage markers, and prebiotic release to digestion or fermentation behavior. Future studies should also report release medium composition, pH, temperature, film thickness, active loading, cumulative release, kinetic model, release exponent, diffusion coefficient when available, and swelling or erosion behavior, so that release mechanisms can be compared across studies without overstating precision or programmability. The next phase of this field will also involve intelligent and adaptive edible interfaces. Colorimetric films, pH responsive systems, freshness indicators, and self-healing materials show that edible coatings can evolve into active quality management tools. These systems can potentially inform consumers, reduce unnecessary food waste, and preserve food quality after minor mechanical damage. However, intelligent systems must be designed carefully. Freshness indicators should be validated against microbial, chemical, and sensory spoilage markers. Color changes must be clear, reproducible, and easy to interpret. Self-healing systems should maintain barrier function without masking unsafe deterioration. Future work should therefore evaluate intelligent edible films not only as materials but also as communication tools between the packaged food and the consumer.

Safety and sensory acceptability should become central design criteria rather than final confirmatory tests. Edible films may contain polymers, plasticizers, crosslinkers, nanofillers, plant extracts, essential oils, probiotics, antimicrobials, pH indicators, or microcapsules. Each component may be acceptable individually, but the final composite must be evaluated under the intended conditions of use. Important issues include cytotoxicity, allergenicity, migration, degradation products, residual processing aids, release dose, sensory changes, odor, color, taste, texture, and repeated dietary exposure. This is particularly important for nanoscale cellulose, essential oil nanoemulsions, probiotic loaded films, active indicators, and self-healing systems. A future validation framework should combine food grade verification, migration analysis, digestion simulation, cytocompatibility, sensory testing, and realistic shelf-life assessment. Scalability is also essential. Most edible films are produced by solution casting at laboratory scale, but industrial translation may require continuous coating, spraying, dipping, extrusion, electrospinning, or thermoplastic processing. Each route has different implications for film thickness, drying time, energy consumption, bioactive stability, microbial safety, and cost. Future studies should report process conditions in a way that supports scale-up, including solids content, drying temperature, drying time, coating thickness, film yield, raw material variability, and compatibility with existing food processing lines. Agricultural residue-derived cellulose and hemicellulose sources offer strong sustainability advantages, but their industrial use will require consistent extraction, purification, and quality control.

Intelligent and adaptive edible interfaces also require more realistic translational evaluation. pH-responsive and colorimetric films are promising for high-value fruits, meat products, and freshness-sensitive foods, while self-healing films may be particularly useful for nuts, lipid-rich foods, and products exposed to mechanical damage during handling. However, these systems must be calibrated against microbial, chemical, and sensory spoilage markers to avoid misleading freshness signals. Future work should optimize polymer ratios, indicator concentration, microcapsule loading, and processing conditions while also considering transparency, odor, taste, consumer interpretation, food safety compliance, and compatibility with scalable manufacturing. Shelf-life modeling and environmentally friendly processing will be essential for moving smart edible interfaces from laboratory prototypes toward practical food packaging applications. Finally, nutrition-oriented claims should be developed cautiously. Edible interfaces containing prebiotics, probiotics, yeast biomass, fermentable hemicellulose structures, or bioactive polyphenols have potential relevance for gut microbiota and functional nutrition. However, most current evidence remains indirect. Simulated gastric release, probiotic survival during storage, or *in vitro* antioxidant activity does not automatically demonstrate human nutritional benefit. Future work should include digestion models, colonic fermentation, short-chain fatty acid analysis, microbiota profiling, metabolomics, and eventually human studies. This would help clarify whether edible films can move beyond preservation and become meaningful contributors to functional food design.

## 14 Conclusion

This review presents cellulose-, hemicellulose-, and chitosan-based films and coatings as multifunctional edible food interfaces whose performance depends on the coordinated relationship between polymer structure, food matrix properties, active cargo behavior, safety, and intended use. The reviewed evidence supports the value of these materials as sustainable and functional alternatives to conventional packaging, but it also shows that their claims should be interpreted according to the level of validation available. Real-food studies provide the strongest support for preservation-oriented applications. In fruits and vegetables, coatings based on chitosan, cellulose derivatives, bacterial cellulose, CMC, and composite polysaccharide systems have been shown to reduce moisture loss, maintain firmness and color, suppress fungal or microbial growth, and delay visible deterioration under defined storage conditions. In meat, seafood, dairy, and lipid-rich foods, polysaccharide films containing polyphenols, essential oils, nanoemulsions, or other active compounds have demonstrated potential to reduce microbial growth, lipid oxidation, pH changes, color deterioration, and texture loss. These findings support the use of edible interfaces for food-specific preservation when performance is validated directly in the target food matrix. A second level of evidence comes from material characterization, food simulants, *in vitro* antimicrobial assays, antioxidant assays, and simulated gastrointestinal release tests. These studies are valuable for understanding structure–function relationships, screening formulations, and identifying release or antimicrobial potential. For example, improvements in tensile strength, WVP/WVTR, oxygen barrier performance, release percentage, inhibition zones, or radical-scavenging activity indicate useful formulation-level performance. However, these endpoints should not be treated as equivalent to real-food shelf-life extension, consumer acceptability, or nutritional efficacy. Inhibition-zone assays or in vitro microbial reductions require confirmation in complex foods, where pH, water activity, fat content, surface roughness, microbial load, storage temperature, coating adhesion, and release kinetics can change performance. Similarly, release in food simulants or simulated gastric/intestinal fluids demonstrates release feasibility, but does not by itself confirm bioaccessibility, colonic delivery, fermentation, microbiota modulation, or health benefit. The most speculative claims concern post-consumption relevance, prebiotic or synbiotic functionality, SCFA production, gut microbiota modulation, and human nutritional benefit. Although cellulose-, hemicellulose-, and chitosan-based films can incorporate probiotics, prebiotic oligosaccharides, polyphenols, essential oils, and other bioactives, most current evidence remains formulation-level or mechanistic. Strong claims related to gut microbiota effects or post-consumption functionality require dose-per-serving estimation, standardized digestion assays, intestinal and colonic fermentation models, microbiota sequencing, metabolomics, animal studies, and ultimately controlled human intervention trials. Until such evidence is available, microbiota-oriented and nutrition-related outcomes should be presented as plausible future directions rather than demonstrated functions.

Several broader limitations also remain unresolved. Quantitative comparison across studies is still restricted by differences in film thickness, conditioning humidity, test temperature, units, active loading, food matrix, microbial strain, storage condition, and statistical reporting. Sensory acceptability, cytotoxicity, migration, degradation products, oral exposure, food-contact status, and regulatory classification are often underexplored, particularly in formulations containing crosslinkers, essential oils, phenolic extracts, nanoemulsions, nanocellulose, probiotics, color indicators, or microcapsules. Intelligent, responsive, and self-healing edible interfaces are promising, but most should still be regarded as proof-of-concept technologies until food-specific calibration, false-signal risks, migration, microcapsule safety, sensory effects, and regulatory status are clarified. Industrial translation also requires pilot-scale validation, reproducible coating or film-forming processes, storage stability, raw-material consistency, and compatibility with existing food-processing systems. Overall, cellulose-, hemicellulose-, and chitosan-based edible interfaces offer a promising route toward sustainable food preservation and functional food innovation, provided that their functions are claimed according to the evidence available. Their near-term value is best supported in food preservation, where real-food validation demonstrates improvements in quality and shelf-life-related parameters. Their delivery and intelligent functions are supported mainly by formulation, simulant, *in vitro*, or proof-of-concept evidence. Their post-consumption, microbiota-related, and human health benefits remain future possibilities requiring stronger biological validation. Future progress should therefore focus on evidence-aware design: selecting the polymer platform according to the target food and deterioration pathway, validating performance under realistic conditions, quantifying release and exposure, ensuring safety and sensory acceptability, and separating demonstrated outcomes from plausible but still unvalidated benefits.

## References

[1] Dhall RK. Advances in edible coatings for fresh fruits and vegetables: a review. Crit Rev Food Sci Nutr. (2013) 53:435–50. doi: 10.1080/10408398.2010.541568, 23391012

[2] Hailu W Bekele T. Quality and phsio-chemical changes associated with processing and properties of fruits and vegetables. Cogent Food Agric. (2024) 10:2419957. doi: 10.1080/23311932.2024.2419957

[3] Olivas GI Barbosa-Cánovas GV. Edible coatings for fresh-cut fruits. Crit Rev Food Sci Nutr. (2005) 45:657–70. doi: 10.1080/10408690490911837, 16371333

[4] Iturralde-García RD Cinco-Moroyoqui FJ Martínez-Cruz O Ruiz-Cruz S Wong-Corral FJ Borboa-Flores J . Emerging technologies for prolonging fresh-cut fruits’ quality and safety during storage. Horticulturae (2022) 8:731. doi: 10.3390/horticulturae8080731

[5] Kaveh S Hashemi SM Abedi E Amiri MJ Conte FL. Bio-preservation of meat and fermented meat products by lactic acid bacteria strains and their antibacterial metabolites. Sustainability (2023) 15:10154. doi: 10.3390/su151310154

[6] Umaraw P Munekata PES Verma AK Barba FJ Singh VP Kumar P . Edible films/coating with tailored properties for active packaging of meat, fish and derived products. Trends Food Sci Technol. (2020) 98:10–24. doi: 10.1016/j.tifs.2020.01.032

[7] Sridhar A Ponnuchamy M Kumar PS Kapoor A. Food preservation techniques and nanotechnology for increased shelf life of fruits, vegetables, beverages and spices: a review. Environ Chem Lett. (2021) 19:1715–35. doi: 10.1007/s10311-020-01126-2, 33192209 PMC7651826

[8] Guerreiro AC Gago CML Faleiro ML Miguel MGC Antunes MDC. The use of polysaccharide-based edible coatings enriched with essential oils to improve shelf-life of strawberries. Postharvest Biol Technol. (2015) 110:51–60. doi: 10.1016/j.postharvbio.2015.06.019

[9] Chen Y Awasthi AK Wei F Tan Q Li J. Single-use plastics: production, usage, disposal, and adverse impacts. Sci Total Environ. (2021) 752:141772. doi: 10.1016/j.scitotenv.2020.141772, 32892042

[10] Asgher M Qamar SA Bilal M Iqbal HMN. Bio-based active food packaging materials: sustainable alternative to conventional petrochemical-based packaging materials. Food Res Int. (2020) 137:109625. doi: 10.1016/j.foodres.2020.109625, 33233213

[11] Salgado PR Ortiz CM Musso YS Di Giorgio L Mauri AN. Edible films and coatings containing bioactives. Curr Opin Food Sci. (2015) 5:86–92. doi: 10.1016/j.cofs.2015.09.004

[12] Galus S Arik Kibar EA Gniewosz M Kraśniewska K. Novel materials in the preparation of edible films and coatings—a review. Coatings (2020) 10:674. doi: 10.3390/coatings10070674

[13] Martins B. A. Albuquerque P. B. S. de Souza M. P. (2022). Bio-based films and coatings: sustainable polysaccharide packaging alternatives for the food industry. J Polym Environ, 30:4023–4039. doi: 10.1007/s10924-022-02442-0

[14] Marotta A Borriello A Khan MR Cavella S Ambrogi V Torrieri E. Boosting food packaging sustainability through the valorization of Agri-food waste and by-products. Polymers (2025) 17:735. doi: 10.3390/polym17060735 40292599 PMC11946487

[15] Mohammadi L Karim A Aghajanzadeh S Kashiri M Khalloufi S. Incorporating dietary fibers in edible coatings and films to enhance the shelf life of food products: formulations, applications, challenges, and perspectives. Food Bioprocess Technol. (2025) 18:6997–7031. doi: 10.1007/s11947-025-03916-4

[16] Chaudhary V Thakur N Kajla P Thakur S Punia S. Application of encapsulation technology in edible films: carrier of bioactive compounds. Front Sustain Food Syst. (2021) 5:734921. doi: 10.3389/fsufs.2021.734921

[17] Yousuf B Wu S Siddiqui MW. Incorporating essential oils or compounds derived thereof into edible coatings: effect on quality and shelf life of fresh/fresh-cut produce. Trends Food Sci Technol. (2021) 108:245–57. doi: 10.1016/j.tifs.2021.01.016

[18] Xie C Wang Y Yang D Zhong Y Fan K. Polysaccharide-based edible film/coating incorporated with nano-antimicrobial agent for improving quality of fruits and vegetables: a review. Food Rev Int. (2026) 42:1624–56. doi: 10.1080/87559129.2025.2524401

[19] Wan X Li N Qiu J Wang Q Feng Y Xiong R. Hierarchical nanocellulose-based functional films: a sustainable and high-performance alternative to conventional plastic films. Biomacromolecules. (2025) 26:5514–46. doi: 10.1021/acs.biomac.5c00783, 40748329

[20] Shaghaleh H Xu X Wang S. Current progress in production of biopolymeric materials based on cellulose, cellulose nanofibers, and cellulose derivatives. RSC Adv. (2018) 8:825–42. doi: 10.1039/C7RA11157F, 35538958 PMC9076966

[21] Mathura SR Landázuri AC Mathura F Andrade Sosa AG Orejuela-Escobar LM. Hemicelluloses from bioresidues and their applications in the food industry – towards an advanced bioeconomy and a sustainable global value chain of chemicals and materials. Sustainable Food Technol. (2024) 2:1183–205. doi: 10.1039/d4fb00035h

[22] Ardean C Davidescu CM Nemeş NS Negrea A Ciopec M Duteanu N . Factors influencing the antibacterial activity of chitosan and chitosan modified by functionalization. Int. J. Mol. Sci. (2021) 22:7449. doi: 10.3390/ijms22147449 34299068 PMC8303267

[23] Ke C-L Deng F-S Chuang C-Y Lin C-H. Antimicrobial actions and applications of chitosan. Polymers (2021) 13:904. doi: 10.3390/polym13060904 33804268 PMC7998239

[24] Priyadarshi R Rhim J-W. Chitosan-based biodegradable functional films for food packaging applications. Innov Food Sci Emerg Technol. (2020) 62:102346. doi: 10.1016/j.ifset.2020.102346

[25] Muñoz-Tebar N Pérez-Álvarez JA Fernández-López J Viuda-Martos M. Chitosan edible films and coatings with added bioactive compounds: antibacterial and antioxidant properties and their application to food products: a review. Polymers (2023) 15:904. doi: 10.3390/polym15020396 36679276 PMC9864592

[26] Xu Y Wu Z Li A Chen N Rao J Zeng Q. Nanocellulose composite films in food packaging materials: a review. Polymers (2024) 16:423. doi: 10.3390/polym16030423 38337312 PMC10856991

[27] Zhao Y Sun H Yang B Weng Y. Hemicellulose-based film: potential green films for food packaging. Polymers (2020) 12:1775. doi: 10.3390/polym12081775 32784786 PMC7465936

[28] Li Z Zhang H He L Hou Y Che Y Liu T . Influence of structural features and feruloylation on fermentability and ability to modulate gut microbiota of arabinoxylan in in vitro fermentation. Front Microbiol. (2023) 13:1113601. doi: 10.3389/fmicb.2022.1113601 36713199 PMC9874102

[29] Álvarez-Mercado AI Plaza-Diaz J. Dietary polysaccharides as modulators of the gut microbiota ecosystem: an update on their impact on health. Nutrients. (2022) 14:4116. doi: 10.3390/nu14194116 36235768 PMC9573424

[30] Portincasa P Bonfrate L Vacca M De Angelis M Farella I Lanza E . Gut microbiota and short chain fatty acids: implications in glucose homeostasis. Int. J. Mol. Sci. (2022) 23:1105. doi: 10.3390/ijms23031105, 35163038 PMC8835596

[31] Deshmukh RK Gaikwad KK. Natural antimicrobial and antioxidant compounds for active food packaging applications. Biomass Convers Biorefinery. (2024) 14:4419–40. doi: 10.1007/s13399-022-02623-w

[32] Bajić M Ročnik T Oberlintner A Scognamiglio F Novak U Likozar B. Natural plant extracts as active components in chitosan-based films: a comparative study. Food Packag Shelf Life. (2019) 21:100365. doi: 10.1016/j.fpsl.2019.100365

[33] Hashemi SM Kaveh S Abedi E Phimolsiripol Y. Polysaccharide-based edible films/coatings for the preservation of meat and fish products: emphasis on incorporation of lipid-based nanosystems loaded with bioactive compounds. Foods. (2023) 12:3268. doi: 10.3390/foods12173268 37685201 PMC10487091

[34] Jamwal V Mittal A. Recent progresses in nanocomposite films for food-packaging applications: synthesis strategies, technological advancements, potential risks and challenges. Food Rev Int. (2024) 40:3634–65. doi: 10.1080/87559129.2024.2368065

[35] Wang M Wichienchot S He X Fu X Huang Q Zhang B. In vitro colonic fermentation of dietary fibers: fermentation rate, short-chain fatty acid production and changes in microbiota. Trends Food Sci Technol. (2019) 88:1–9. doi: 10.1016/j.tifs.2019.03.005

[36] Wijesekara T Xu B. New insights into sources, bioavailability, health-promoting effects, and applications of chitin and chitosan. J Agric Food Chem. (2024) 72:17138–52. doi: 10.1021/acs.jafc.4c02162, 39042786

[37] Wu Y Wu H Hu L. Recent advances of proteins, polysaccharides and lipids-based edible films/coatings for food packaging applications: a review. Food Biophys. (2024) 19:29–45. doi: 10.1007/s11483-023-09794-7

[38] Gupta V Biswas D Roy S. A comprehensive review of biodegradable polymer-based films and coatings and their food packaging applications. Materials (2022) 15:5899. doi: 10.3390/ma15175899 36079280 PMC9457097

[39] Yu C Sun H Yao L Weng Y. Polysaccharide-based food packaging materials: structural engineering, functional modifications, and sustainable applications. Macromol Mater Eng. (2026) 311:e00363. doi: 10.1002/mame.202500363

[40] Guo J Ding K Li S Li S Jin P Zheng Y . Polysaccharide-based high barrier food packaging film: design and application. Crit Rev Food Sci Nutr. (2025) 65:7651–70. doi: 10.1080/10408398.2025.2476118, 40057960

[41] Anis A Pal K Al-Zahrani SM. Essential oil-containing polysaccharide-based edible films and coatings for food security applications. Materials (2021) 13:575. doi: 10.3390/polym13040575 PMC7917627 33672974

[42] Kong I Degraeve P Pui LP. Polysaccharide-based edible films incorporated with essential oil nanoemulsions: physico-chemical, mechanical properties and its application in food preservation—a review. Foods (2022) 11:555. doi: 10.3390/foods11040555 35206032 PMC8871330

[43] Cruz-Monterrosa RG Rayas-Amor AA González-Reza RM Zambrano-Zaragoza ML Aguilar-Toalá JE Liceaga AM. Application of polysaccharide-based edible coatings on fruits and vegetables: improvement of food quality and bioactivities. Polysaccharides (2023) 4:99–115. doi: 10.3390/polysaccharides4020008

[44] Quirós-Sauceda AE Ayala-Zavala JF Olivas GI González-Aguilar GA. Edible coatings as encapsulating matrices for bioactive compounds: a review. J Food Sci Technol. (2014) 51:1674–85. doi: 10.1007/s13197-013-1246-x, 25190824 PMC4152518

[45] Shen Y Seidi F Ahmad M Liu Y Saeb MR Akbari A . Recent advances in functional cellulose-based films with antimicrobial and antioxidant properties for food packaging. J Agric Food Chem. (2023) 71:16469–87. doi: 10.1021/acs.jafc.3c06004, 37877425

[46] He C Yuan L Bi S Zhou C Yang Q Gu J . Modified chitosan-based coating/packaging composites with enhanced antibacterial, antioxidant, and UV-resistant properties for fresh food preservation. ACS Appl Mater Interfaces. (2024) 16:48352–62. doi: 10.1021/acsami.4c10643, 39221854

[47] Xu J Xia R Yuan T Sun R. Use of xylooligosaccharides (XOS) in hemicelluloses/chitosan-based films reinforced by cellulose nanofiber: effect on physicochemical properties. Food Chem. (2019) 298:125041. doi: 10.1016/j.foodchem.2019.125041, 31261000

[48] Nwankwo JA Liu W Guo X Lin Y Hussain M Khan I . Microemulsion gel systems: formulation, stability studies, biopolymer interactions, and functionality in food product development. Compr Rev Food Sci Food Saf. (2025) 24:e70110. doi: 10.1111/1541-4337.70110, 39898912

[49] Vasile C Baican M. Progresses in food packaging, food quality, and safety—controlled-release antioxidant and/or antimicrobial packaging. Molecules (2021) 26:1263. doi: 10.3390/molecules26051263 33652755 PMC7956554

[50] Xie F. Biopolymer-based multilayer films and coatings for food preservation: an update of the recent development. Curr Food Sci Technol Rep. (2023) 1:1–12. doi: 10.1007/s43555-023-00002-8

[51] Bayer IS. Biopolymers in multilayer films for long-lasting protective food packaging: a review. Sustainable Food Packag Technol. Weinheim, Germany: Wiley-VCH (2021) 395–426. doi: 10.1002/9783527820078.ch15,

[52] Avila LB Schnorr C Silva LFO Morais MM Moraes CC da Rosa GS . Trends in bioactive multilayer films: perspectives in the use of polysaccharides, proteins, and carbohydrates with natural additives for application in food packaging. Foods (2023) 12:1692. doi: 10.3390/foods12081692 37107487 PMC10137676

[53] Wang Q Chen W Zhu W McClements DJ Liu X Liu F. A review of multilayer and composite films and coatings for active biodegradable packaging. NPJ Sci Food. (2022) 6:18. doi: 10.1038/s41538-022-00132-8, 35277514 PMC8917176

[54] Mkandawire M Aryee ANA. Resurfacing and modernization of edible packaging material technology. Curr Opin Food Sci. (2018) 19:104–12. doi: 10.1016/j.cofs.2018.03.010

[55] Wu D Lin Y Xu X Shi B Wang Y Wang L . A multifunctional zein/tannic acid/stearic acid edible coating with lemongrass essential oil for enhanced preservation of cherry tomatoes. Int J Biol Macromol. (2026) 345:150627. doi: 10.1016/j.ijbiomac.2026.150627, 41619882

[56] Pires J Paula CD Souza VG Fernando AL Coelhoso I. Understanding the barrier and mechanical behavior of different nanofillers in chitosan films for food packaging. Polymers (2021) 13:721. doi: 10.3390/polym13050721 33653012 PMC7956210

[57] Garavand F Cacciotti I Vahedikia N Rehman A Tarhan Ö Akbari-Alavijeh S . A comprehensive review on the nanocomposites loaded with chitosan nanoparticles for food packaging. Crit Rev Food Sci Nutr. (2022) 62:1383–416. doi: 10.1080/10408398.2020.1843133, 33153290

[58] Wu W Wu Y Lin Y Shao P. Facile fabrication of multifunctional citrus pectin aerogel fortified with cellulose nanofiber as controlled packaging of edible fungi. Food Chem. (2022) 374:131763. doi: 10.1016/j.foodchem.2021.131763, 34896953

[59] Jung J Deng Z Zhao Y. A review of cellulose nanomaterials incorporated fruit coatings with improved barrier property and stability: principles and applications. J Food Process Eng. (2020) 43:e13344. doi: 10.1111/jfpe.13344

[60] Bizymis A-P Tzia C. Edible films and coatings: properties for the selection of the components, evolution through composites and nanomaterials, and safety issues. Crit Rev Food Sci Nutr. (2022) 62:8777–92. doi: 10.1080/10408398.2021.1934652, 34098828

[61] Zhao Y Li B Li C Xu Y Luo Y Liang D . Comprehensive review of polysaccharide-based materials in edible packaging: a sustainable approach. Polymers (2021) 10:1845. doi: 10.3390/foods10081845 PMC8392450 34441621

[62] Antony Jose S Cowan N Davidson M Godina G Smith I Xin J . A comprehensive review on cellulose nanofibers, nanomaterials, and composites: manufacturing, properties, and applications. Polymers (2025) 15:356. doi: 10.3390/nano15050356 PMC11901645 40072159

[63] Qi Y Guo Y Liza AA Yang G Sipponen MH Guo J . Nanocellulose: a review on preparation routes and applications in functional materials. Cellulose. (2023) 30:4115–47. doi: 10.1007/s10570-023-05169-w

[64] Ilyas RA Azmi A Nurazzi NM Atiqah A Atikah MSN Ibrahim R . Oxygen permeability properties of nanocellulose reinforced biopolymer nanocomposites. Mater Today Proc. (2022) 52:2414–9. doi: 10.1016/j.matpr.2021.10.420, 38826717

[65] de Lima GG Zakaluk ICB Artner MA Pedro AC Gonzalez de Cademartori PH Muniz GIB d . Enhancing barrier and antioxidant properties of nanocellulose films for coatings and active packaging: a review. ACS Appl Nano Mater. (2025) 8:4397–421. doi: 10.1021/acsanm.4c04805

[66] Yu H-Y Yan C-F. "Mechanical properties of cellulose nanofibril (CNF)- and cellulose nanocrystal (CNC)-based nanocomposites". In: Kargarzadeh H Ahmad I Thomas S Dufresne A, editors. Handbook of Nanocellulose and Cellulose Nanocomposites. Germany: Wiley-VCH (2017). doi: 10.1002/9783527689972.ch12

[67] Zhang H Su S Liu S Qiao C Wang E Chen H . Effects of chitosan and cellulose derivatives on sodium carboxymethyl cellulose-based films: a study of rheological properties of film-forming solutions. Molecules (2023) 28:5211. doi: 10.3390/molecules28135211 37446873 PMC10343429

[68] Kasle P Bains A Goksen G Dhull SB Ali N Nagarik R . Exploring the properties of carboxymethyl cellulose blended with other polymeric compounds for the formulation of biodegradable packaging films and edible coatings: a review. Compr Rev Food Sci Food Saf. (2025) 24:e70215. doi: 10.1111/1541-4337.70215, 40556258

[69] Xu Y Deng Q Ruan C Xu D Zeng K. Application of carboxymethyl cellulose and its edible composite coating in fruit preservation. Packag Technol Sci. (2024) 37:781–92. doi: 10.1002/pts.2822

[70] Lu Y He Q Fan G Cheng Q Song G. Extraction and modification of hemicellulose from lignocellulosic biomass: a review. Green Processing and Synthesis (2021) 10:779–804. doi: 10.1515/gps-2021-0065

[71] Gautam D Rana V Sharma S Kumar Walia Y Kumar K Umar A . Hemicelluloses: a review on extraction and modification for various applications. ChemistrySelect. (2025) 10:e06050. doi: 10.1002/slct.202406050

[72] Hansen NML Plackett D. Sustainable films and coatings from hemicelluloses: a review. Biomacromolecules. (2008) 9:1493–505. doi: 10.1021/bm800053z, 18457452

[73] Xu J Xia R Zheng L Yuan T Sun R. Plasticized hemicelluloses/chitosan-based edible films reinforced by cellulose nanofiber with enhanced mechanical properties. Carbohydr Polym. (2019) 224:115164. doi: 10.1016/j.carbpol.2019.115164, 31472866

[74] Zhang Y Zhao Y Yang W Song G Zhong P Ren Y . Structural complexity of konjac glucomannan and its derivatives governs the diversity and outputs of gut microbiota. Carbohydr Polym. (2022) 292:119639. doi: 10.1016/j.carbpol.2022.119639, 35725199

[75] Fenice M Gorrasi S. Advances in chitin and chitosan science. Molecules (2021) 26:1805. doi: 10.3390/molecules26061805 33806913 PMC8005133

[76] Gomes LC Faria SI Valcarcel J Vázquez JA Cerqueira MA Pastrana L . The effect of molecular weight on the antimicrobial activity of chitosan from *Loligo opalescens* for food packaging applications. Marine Drugs (2021) 19:384. doi: 10.3390/md19070384 34356809 PMC8303414

[77] Chen JL Zhao Y. Effect of molecular weight, acid, and plasticizer on the physicochemical and antibacterial properties of β-chitosan based films. J Food Sci. (2012) 77:E127–36. doi: 10.1111/j.1750-3841.2012.02686.x, 23163939

[78] Aranaz I Alcántara AR Civera MC Arias C Elorza B Heras Caballero A . Chitosan: an overview of its properties and applications. Polymers (2021) 13:3256. doi: 10.3390/polym13193256 34641071 PMC8512059

[79] Wang L Dekker M Heising J Zhao L Fogliano V. Food matrix design can influence the antimicrobial activity in the food systems: a narrative review. Crit Rev Food Sci Nutr. (2024) 64:8963–89. doi: 10.1080/10408398.2023.2205937, 37154045

[80] Cao J Gao M Wang J Liu Y Zhang X Ping Y . Construction of nano slow-release systems for antibacterial active substances and its applications: a comprehensive review. Front Nutr. (2023) 10:1109204. doi: 10.3389/fnut.2023.1109204 36819707 PMC9928761

[81] Ma Z Garrido-Maestu A Jeong KC. Application, mode of action, and in vivo activity of chitosan and its micro- and nanoparticles as antimicrobial agents: a review. Carbohydr Polym. (2017) 176:257–65. doi: 10.1016/j.carbpol.2017.08.082, 28927606

[82] Pawase PA Rout S Tripathy S Pathare AM Srivastav PP Bashir O . Recent advances in cellulose, chitosan, and protein-based edible films for sustainable food packaging: a comprehensive review. Int J Biol Macromol. (2025) 321:146172. doi: 10.1016/j.ijbiomac.2025.146172, 40701483

[83] Rojas-Lema S Nilsson K Trifol J Langton M Gomez-Caturla J Balart R . Faba bean protein films reinforced with cellulose nanocrystals as edible food packaging material. Food Hydrocoll. (2021) 121:107019. doi: 10.1016/j.foodhyd.2021.107019

[84] Atta OM Manan S Ahmed AA Awad MF Ul-Islam M Subhan F . Development and characterization of yeast-incorporated antimicrobial cellulose biofilms for edible food packaging application. Polymers (2021) 13:2310. doi: 10.3390/polym13142310 34301067 PMC8309339

[85] Wardak MH Nkede FN Van TT Meng F Tanaka F Tanaka F. Development of edible films and partial coating, a novel coating technique for tomato fruits, using citric acid-crosslinked starch and cellulose nanofiber. Prog Org Coat. (2024) 187:108127. doi: 10.1016/j.porgcoat.2023.108127

[86] Liu Z Lin D Lopez-Sanchez P Yang X. Characterizations of bacterial cellulose nanofibers reinforced edible films based on konjac glucomannan. Int J Biol Macromol. (2020) 145:634–45. doi: 10.1016/j.ijbiomac.2019.12.109, 31857167

[87] Bilbao-Sáinz C Avena-Bustillos RJ Wood DF Williams TG McHugh TH. Composite edible films based on hydroxypropyl methylcellulose reinforced with microcrystalline cellulose nanoparticles. J Agric Food Chem. (2010) 58:3753–60. doi: 10.1021/jf9033128, 20187652

[88] Zhao J Liu T Xia K Liu X Zhang X. Preparation and application of edible agar-based composite films modified by cellulose nanocrystals. Food Packag Shelf Life. (2022) 34:100936. doi: 10.1016/j.fpsl.2022.100936

[89] Papadaki A Manikas AC Papazoglou E Kachrimanidou V Lappa I Galiotis C . Whey protein films reinforced with bacterial cellulose nanowhiskers: improving edible film properties via a circular economy approach. Food Chem. (2022) 385:132604. doi: 10.1016/j.foodchem.2022.132604, 35303655

[90] Qi W Tong X Wang M Liu S Cheng J Wang H. Impact of soybean protein isolate concentration on chitosan-cellulose nanofiber edible films: focus on structure and properties. Int J Biol Macromol. (2024) 255:128185. doi: 10.1016/j.ijbiomac.2023.128185, 37977456

[91] Choque-Quispe D Choque-Quispe Y Ligarda-Samanez CA Peralta-Guevara DE Solano-Reynoso AM Ramos-Pacheco BS . Effect of the addition of corn husk cellulose nanocrystals in the development of a novel edible film. Nanomaterials (2022) 12:3421. doi: 10.3390/nano12193421 36234547 PMC9565820

[92] Nongnual T Butprom N Boonsang S Kaewpirom S. Citric acid crosslinked carboxymethyl cellulose edible films: a case study on preserving freshness in bananas. Int J Biol Macromol. (2024) 267:131135. doi: 10.1016/j.ijbiomac.2024.131135, 38574914

[93] Su J-F Huang Z Yuan X-Y Wang X-Y Li M. Structure and properties of carboxymethyl cellulose/soy protein isolate blend edible films crosslinked by Maillard reactions. Carbohydr Polym. (2010) 79:145–53. doi: 10.1016/j.carbpol.2009.07.035

[94] Mu C Guo J Li X Lin W Li D. Preparation and properties of dialdehyde carboxymethyl cellulose crosslinked gelatin edible films. Food Hydrocoll. (2012) 27:22–9. doi: 10.1016/j.foodhyd.2011.09.005

[95] Sutharsan J Boyer CA Zhao J. Physicochemical properties of chitosan edible films incorporated with different classes of flavonoids. Carbohydr Polym Technol Appl. (2022) 4:100232. doi: 10.1016/j.carpta.2022.100232

[96] Wang Y Li J Guo X Wang H Qian F Lv Y. Active biodegradable polyvinyl alcohol–hemicellulose/tea polyphenol films with excellent moisture resistance prepared via ultrasound assistance for food packaging. Coatings (2021) 11:219. doi: 10.3390/coatings11020219

[97] Lin L Peng S Shi C Li C Hua Z Cui H. Preparation and characterization of cassava starch/sodium carboxymethyl cellulose edible film incorporating apple polyphenols. Int J Biol Macromol. (2022) 212:155–64. doi: 10.1016/j.ijbiomac.2022.05.121, 35609834

[98] Yekta R Mirmoghtadaie L Hosseini H Norouzbeigi S Hosseini SM Shojaee-Aliabadi S. Development and characterization of a novel edible film based on *Althaea rosea* flower gum: investigating the reinforcing effects of bacterial nanocrystalline cellulose. Int J Biol Macromol. (2020) 158:327–37. doi: 10.1016/j.ijbiomac.2020.04.021, 32278602

[99] Cai R Jia L Yang R Tao H Cui H Lin L . Fabrication of guar gum/chitosan edible films reinforced with orange essential oil nanoemulsion for cheese preservation. Int J Biol Macromol. (2025) 285:138285. doi: 10.1016/j.ijbiomac.2024.138285, 39631598

[100] Kaya M Ravikumar P Ilk S Mujtaba M Akyuz L Labidi J . Production and characterization of chitosan based edible films from *Berberis crataegina*'s fruit extract and seed oil. Innov Food Sci Emerg Technol. (2018) 45:287–97. doi: 10.1016/j.ifset.2017.11.013

[101] Kumar N Pratibha Trajkovska Petkoska A Khojah E Sami R Al-Mushhin AAM. Chitosan edible films enhanced with pomegranate peel extract: study on physical, biological, thermal, and barrier properties. Materials (2021) 14:3305. doi: 10.3390/ma14123305 34203852 PMC8232757

[102] Moghimi R Aliahmadi A Rafati H. Antibacterial hydroxypropyl methyl cellulose edible films containing nanoemulsions of Thymus daenensis essential oil for food packaging. Carbohydr Polym. (2017) 175:241–8. doi: 10.1016/j.carbpol.2017.07.086, 28917862

[103] Ruan C Zhang Y Wang J Sun Y Gao X Xiong G . Preparation and antioxidant activity of sodium alginate and carboxymethyl cellulose edible films with epigallocatechin gallate. Int J Biol Macromol. (2019) 134:1038–44. doi: 10.1016/j.ijbiomac.2019.05.143, 31128181

[104] Talón E Trifkovic KT Nedovic VA Bugarski BM Vargas M Chiralt A . Antioxidant edible films based on chitosan and starch containing polyphenols from thyme extracts. Carbohydr Polym. (2017) 157:1153–61. doi: 10.1016/j.carbpol.2016.10.080, 27987818

[105] Antoniou J Liu F Majeed H Zhong F. Characterization of tara gum edible films incorporated with bulk chitosan and chitosan nanoparticles: a comparative study. Food Hydrocoll. (2015) 44:309–19. doi: 10.1016/j.foodhyd.2014.09.023

[106] Chen H Meng X Zhang F Chen J Ding X Jian T . Development of chitosan-carboxymethyl cellulose edible films loaded with blackberry anthocyanins and tea polyphenols and their application in beef preservation. Food Hydrocoll. (2025) 164:111198. doi: 10.1016/j.foodhyd.2025.111198

[107] Yanti NA Ahmad SW Ramadhan LO Jamili Muzuni Walhidayah T . Properties and application of edible modified bacterial cellulose film based sago liquid waste as food packaging. Polymers (2021) 13:3570. doi: 10.3390/polym13203570 34685329 PMC8538080

[108] Wang J Liu J Li Z Li Q. Mechanical robust, pH responsive hemicellulose-based PVA film with barrier performance for fruit preservation and monitoring. Food Chem. (2025) 481:144028. doi: 10.1016/j.foodchem.2025.144028, 40184924

[109] Osorio FA Molina P Matiacevich S Enrione J Skurtys O. Characteristics of hydroxy propyl methyl cellulose (HPMC) based edible film developed for blueberry coatings. Procedia Food Sci. (2011) 1:287–93. doi: 10.1016/j.profoo.2011.09.045

[110] Firdous N Moradinezhad F Farooq F Dorostkar M. Advances in formulation, functionality, and application of edible coatings on fresh produce and fresh-cut products: a review. Food Chem. (2023) 407:135186. doi: 10.1016/j.foodchem.2022.135186, 36525802

[111] Tokatlı K Demirdöven A. Effects of chitosan edible film coatings on the physicochemical and microbiological qualities of sweet cherry (*Prunus avium* L.). Sci Hortic. (2020) 259:108656. doi: 10.1016/j.scienta.2019.108656

[112] Pavinatto A de Almeida Mattos AV Malpass ACG Okura MH Balogh DT Sanfelice RC. Coating with chitosan-based edible films for mechanical/biological protection of strawberries. Int J Biol Macromol. (2020) 151:1004–11. doi: 10.1016/j.ijbiomac.2019.11.076, 31726134

[113] Hussain SA Sharma BK Qi PX Yadav MP Jin TZ. Antimicrobial and physicochemical properties of hemicellulose-based films incorporating carvacrol (2025) 17:2073. doi: 10.3390/polym17152073 PMC12349191 40808121

[114] Dai L Wang X Zhang J Li C. Application of chitosan and its derivatives in postharvest coating preservation of fruits. Foods (2025) 14:1318. doi: 10.3390/foods14081318 40282720 PMC12025909

[115] Wang J Yuan Y Liu Y Li X Wu S. Application of chitosan in fruit preservation: a review. Food Chemistry: X. (2024) 23:101589. doi: 10.1016/j.fochx.2024.101589, 39036472 PMC11260026

[116] Miteluț AC Popa EE Drăghici MC Popescu PA Popa VI Bujor O-C . Latest developments in edible coatings on minimally processed fruits and vegetables: a review. Foods (2021) 10:2821. doi: 10.3390/foods10112821 34829101 PMC8620870

[117] Sutharsan J Boyer CA Zhao J. Biological properties of chitosan edible films incorporated with different classes of flavonoids and their role in preserving the quality of chilled beef. Food Hydrocoll. (2023) 139:108508. doi: 10.1016/j.foodhyd.2023.108508

[118] Halloub A Nekhlaoui S Raji M Essabir H Bensalah M-o Bouhfid R . UV-activated self-healing food packaging: ω-3 based microcapsules in hemicellulose/chitosan blend film for cashew nut preservation. Food Biosci. (2024) 60:104313. doi: 10.1016/j.fbio.2024.104313

[119] Mugwagwa LR Chimphango AFA. Physicochemical properties and potential application of hemicellulose/pectin/nanocellulose biocomposites as active packaging for fatty foods. Food Packag Shelf Life. (2022) 31:100795. doi: 10.1016/j.fpsl.2021.100795

[120] Ebrahimi B Mohammadi R Rouhi M Mortazavian AM Shojaee-Aliabadi S Koushki MR. Survival of probiotic bacteria in carboxymethyl cellulose-based edible film and assessment of quality parameters. LWT. (2018) 87:54–60. doi: 10.1016/j.lwt.2017.08.066

[121] Thi Nguyen T Pham B-TT Nhien Le H Bach LG Thuc CNH. Comparative characterization and release study of edible films of chitosan and natural extracts. Food Packag Shelf Life. (2022) 32:100830. doi: 10.1016/j.fpsl.2022.100830

[122] Pham B-TT Nguyen HTD Nguyen TT. Release study of polyphenols from polyvinyl alcohol/chitosan active films supplemented with *Sonneratia ovata* extract and *Piper betel* (L.) essential oil. Food Packag Shelf Life. (2023) 40:101174. doi: 10.1016/j.fpsl.2023.101174

[123] Reyes-Avalos MC Minjares-Fuentes R Femenia A Contreras-Esquivel JC Quintero-Ramos A Esparza-Rivera JR . Application of an alginate–chitosan edible film on figs (*Ficus carica*): effect on bioactive compounds and antioxidant capacity. Food Bioprocess Technol. (2019) 12:499–511. doi: 10.1007/s11947-018-2226-y

[124] Abugoch L Tapia C Plasencia D Pastor A Castro-Mandujano O López L . Shelf-life of fresh blueberries coated with quinoa protein/chitosan/sunflower oil edible film. J Sci Food Agric. (2016) 96:619–26. doi: 10.1002/jsfa.7132, 25678380

[125] Nunes C Silva M Farinha D Sales H Pontes R Nunes J. Edible coatings and future trends in active food packaging–fruits’ and traditional sausages’ shelf life increasing. Foods (2023) 12:3308. doi: 10.3390/foods12173308 37685240 PMC10486622

[126] Alemu TT Intipunya P Gebeyo BA. A comprehensive review of edible coatings for postharvest management of fruits and vegetables: enhancing food and nutrition security. Discover Agric. (2025) 3:190. doi: 10.1007/s44279-025-00348-8

[127] Chen J Chai J Chen X Huang M Zeng X Xu X. Development of edible films by incorporating nanocrystalline cellulose and anthocyanins into modified myofibrillar proteins. Food Chem. (2023) 417:135820. doi: 10.1016/j.foodchem.2023.135820, 36940514

[128] Arif S Maan AA Aadil RM Khan MKI Nazir A. Fabrication of biodegradable composite packaging films from cellulose, hemicellulose and lignin extracted from canola residue. J Food Meas Charact. (2026) 20:2894–909. doi: 10.1007/s11694-025-03906-2

[129] Homez-Jara A Daza LD Aguirre DM Muñoz JA Solanilla JF Váquiro HA. Characterization of chitosan edible films obtained with various polymer concentrations and drying temperatures. Int J Biol Macromol. (2018) 113:1233–40. doi: 10.1016/j.ijbiomac.2018.03.057, 29548921

[130] Pinzon MI Garcia OR Villa CC. The influence of *Aloe vera* gel incorporation on the physicochemical and mechanical properties of banana starch-chitosan edible films. J Sci Food Agric. (2018) 98:4042–9. doi: 10.1002/jsfa.8915, 29377147

[131] Sutharsan J Boyer CA Zhao J. Effect of molecular weight and drying temperature on the physicochemical properties of chitosan edible film. JSFA Rep. (2023) 3:387–96. doi: 10.1002/jsf2.142

[132] Wang H Ding F Ma L Zhang Y. Edible films from chitosan-gelatin: physical properties and food packaging application. Food Biosci. (2021) 40:100871. doi: 10.1016/j.fbio.2020.100871

[133] da Silva Braga R Poletto M. Preparation and characterization of hemicellulose films from sugarcane bagasse. Materials (2020) 13:941. doi: 10.3390/ma13040941 32093209 PMC7079596

[134] Reyes-Avalos MC Femenia A Minjares-Fuentes R Contreras-Esquivel JC Aguilar-González CN Esparza-Rivera JR . Improvement of the quality and the shelf life of figs (*Ficus carica*) using an alginate–chitosan edible film. Food Bioprocess Technol. (2016) 9:2114–24. doi: 10.1007/s11947-016-1796-9

[135] Bonilla J Bittante AMQB Sobral PJA. Thermal analysis of gelatin–chitosan edible film mixed with plant ethanolic extracts. J Therm Anal Calorim. (2017) 130:1221–7. doi: 10.1007/s10973-017-6472-4

[136] Genskowsky E Puente LA Pérez-Álvarez JA Fernandez-Lopez J Muñoz LA Viuda-Martos M. Assessment of antibacterial and antioxidant properties of chitosan edible films incorporated with maqui berry (*Aristotelia chilensis*). LWT-Food Sci Technol. (2015) 64:1057–62. doi: 10.1016/j.lwt.2015.07.026

[137] Sayanjali S Ghanbarzadeh B Ghiassifar S. Evaluation of antimicrobial and physical properties of edible film based on carboxymethyl cellulose containing potassium sorbate on some mycotoxigenic *aspergillus* species in fresh pistachios. LWT-Food Sci Technol. (2011) 44:1133–8. doi: 10.1016/j.lwt.2010.12.017

[138] Rajeswari A Christy EJS Swathi E Pius A. Fabrication of improved cellulose acetate-based biodegradable films for food packaging applications. Environ Chem Ecotoxicol. (2020) 2:107–14. doi: 10.1016/j.enceco.2020.07.003

